# Kekulé diradicaloids derived from a classical N-heterocyclic carbene†
†Electronic supplementary information (ESI) available. CCDC 1826567–1826570. For ESI and crystallographic data in CIF or other electronic format see DOI: 10.1039/c8sc01209a


**DOI:** 10.1039/c8sc01209a

**Published:** 2018-04-24

**Authors:** Dennis Rottschäfer, Beate Neumann, Hans-Georg Stammler, Diego M. Andrada, Rajendra S. Ghadwal

**Affiliations:** a Anorganische Molekülchemie und Katalyse , Lehrstuhl für Anorganische Chemie und Strukturchemie , Centrum für Molekulare Materialien , Fakultät für Chemie , Universität Bielefeld , Universitätsstr. 25 , D-33615 Bielefeld , Germany . Email: rghadwal@uni-bielefeld.de ; http://www.ghadwalgroup.de ; Fax: +49 521 106 6026 ; Tel: +49 521 106 6167; b Allgemeine und Anorganische Chemie , Universität des Saarlandes , Campus C4.1 , D-66123 Saarbrücken , Germany

## Abstract

The direct double carbenylation of 1,4-diiodobenzene and 4,4′-dibromobiphenyl with a classical N-heterocyclic carbene, SIPr (**1**) (SIPr = :C{*N*(2,6-iPr_2_C_6_H_3_)}_2_CH_2_CH_2_), by means of nickel catalysis gives rise to 1,3-imidazolinium salts [(SIPr)(C_6_H_4_)(SIPr)](I)_2_ (**2**) and [(SIPr)(C_6_H_4_)_2_(SIPr)](Br)_2_ (**3**) as off-white solids. Two-electron reduction of **2** and **3** with KC_8_ cleanly yields Kekulé diradicaloid compounds [(SIPr)(C_6_H_4_)(SIPr)] (**4**) and [(SIPr)(C_6_H_4_)_2_(SIPr)] (**5**), respectively, as crystalline solids. Structural parameters and DFT as well as CASSCF calculations suggest the closed-shell singlet ground state for **4** and **5**. Calculations reveal a very low singlet–triplet energy gap Δ*E*_S–T_ for **5** (10.7 kcal mol^–1^), while Δ*E*_S–T_ for **4** (29.1 kcal mol^–1^) is rather large.

## Abstract 2

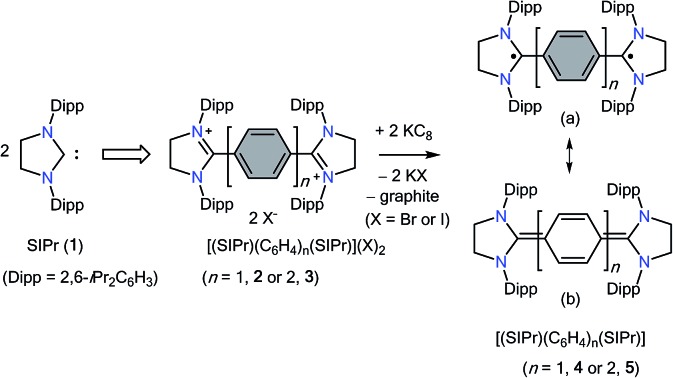
Two-electron reduction of bis(1,3-imidazolinium) salts **2** and **3** with KC_8_ gives rise to stable diradicaloids **4** and **5**, respectively. Calculations reveal a very low singlet–triplet energy gap Δ*E*_S–T_ for **5** (10.7 kcal mol^–1^), while Δ*E*_S–T_ for **4** (29.1 kcal mol^–1^) is rather large.

## Introduction

**Fig. 1:**
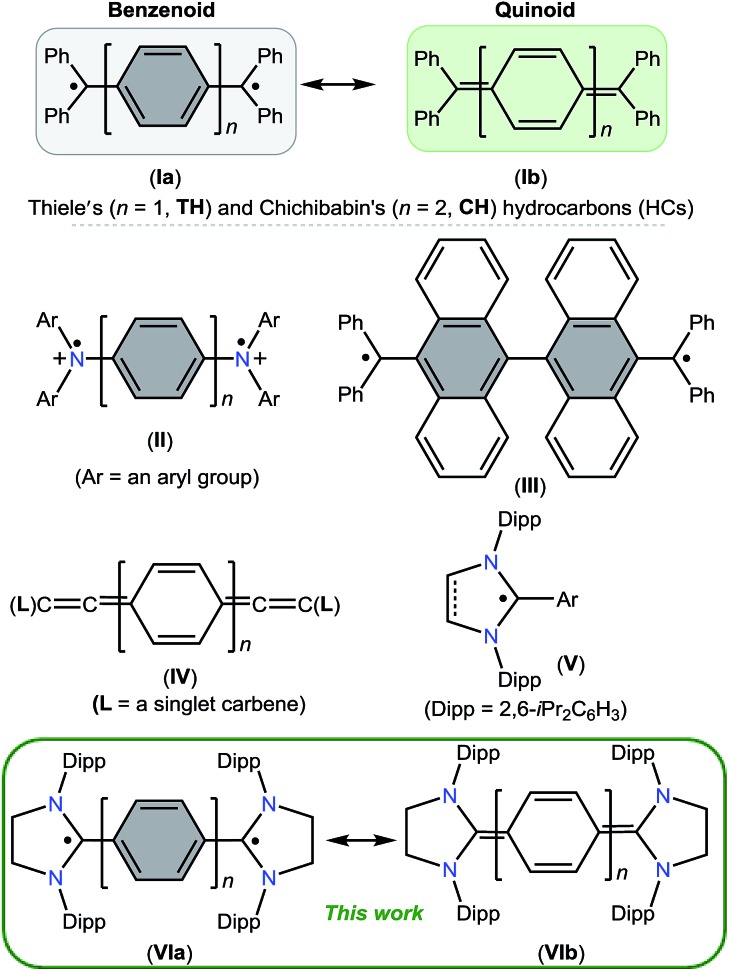
Benzenoid (**Ia**) and quinoid (**Ib**) resonance forms of Thiele's (*n* = 1) and Chichibabin's (*n* = 2) HCs and analogues dicationic bis(triarylamino) (**II**) and benzannulated (**III**) derivatives. Singlet carbenes derived cumulene/diradicaloid compounds (**IV**) and radicals (**V**). NHC derived diradicaloids **VI**.

Molecules containing two unpaired electrons in two nearly degenerate molecular orbitals are called diradicals, which may have parallel (triplet) or antiparallel (singlet) spins.^1^ Depending on the interaction between unpaired electrons, singlet diradicals are further classified as open-shell (OS) and closed-shell (CS) singlets. Diradicaloids are molecules with partial singlet diradical nature in their ground state.^2^ In this context, Thiele's (**TH**)^3^ and Chichibabin's (**CH**)^4^ hydrocarbons **I** (Fig. 1), reported shortly after Gomberg's discovery^5^ of the Ph_3_C˙ radical in 1900, are noteworthy examples. They can be described either as an OS diradical (**Ia**) featuring a phenylene or diphenylene linker or a CS *p*-quinodimethane (**Ib**). Since their first isolation, the ground state spin state of **I** has been a subject of intense experimental and theoretical investigations,^6^ leading to a very controversial discussion, the so-called “diradical paradox”.^7^ In 1986, Montgomery *et al.*^8^ determined the solid-state molecular structures of these highly reactive compounds by single crystal X-ray diffraction. Currently, **CH** is generally described as a diradicaloid (the resonance hybrid of **1a** and **1b**) with a significant diradical character, whereas **TH** is considered as a *p*-quinodimethane.

Stable diradicaloids delocalized over π-conjugated systems are appealing synthetic targets because of their unique physical properties and potential applications in nonlinear optics, molecular electronics, and organic spintronics.^9^ Therefore, different synthetic strategies have been developed over the past few years to thermodynamically and/or kinetically stabilize various analogues of **TH** and **CH**. Bis(triarylamine) dications **II** are isoelectronic to **I**, in which the Ph_2_C˙ moiety of **I** is replaced by an Ar_2_N^+^˙ unit.^10^ The benzannulation approach has also been successfully employed to isolate stable polycyclic hydrocarbons such as **III**.^11^

In recent years, various paramagnetic compounds featuring main-group elements or transition metals have been stabilized by the use of Bertrand's cyclic alkyl amino carbenes (CAACs).^12^ Remarkably, during the preparation of this manuscript for submission, we came across three consecutive reports dealing with the synthesis of extended cumulenes **IV** supported by CAACs^13^ or a classical N-heterocyclic carbene (NHC).^14^ Very recently, we reported stable crystalline radicals **V** derived from classical NHCs.^15^ While these reports nicely emphasize the relevance of singlet carbenes in the synthesis of carbon-centered π-conjugated systems and radicals, synthetic access to diradicaloids **VI**, which are NHC-analogues of **I**, remained a challenge. Herein, we report the synthesis and characterization of two crystalline Kekulé diradicaloid compounds [(SIPr)(C_6_H_4_)(SIPr)] (**4**) and [(SIPr)(C_6_H_4_)_2_(SIPr)] (**5**) derived from a classical NHC, SIPr (**1**) (SIPr = :C{*N*(2,6-iPr_2_C_6_H_3_)}_2_CH_2_CH_2_).

## Results and discussion

**Scheme 1:**
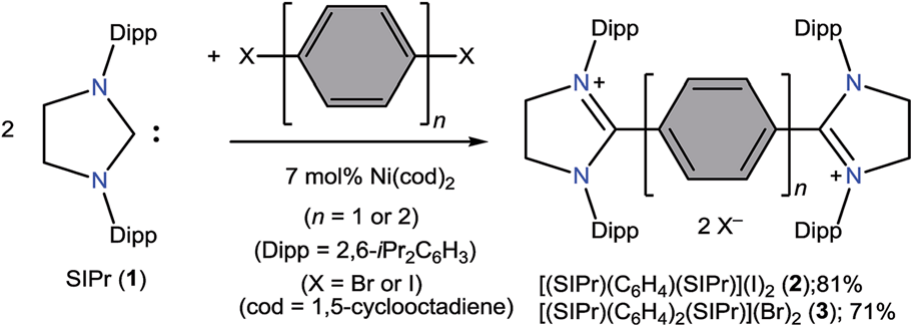
Synthesis of C2-arylated bis(1,3-imidazolinium) salts **2** and **3**.

**Fig. 2:**
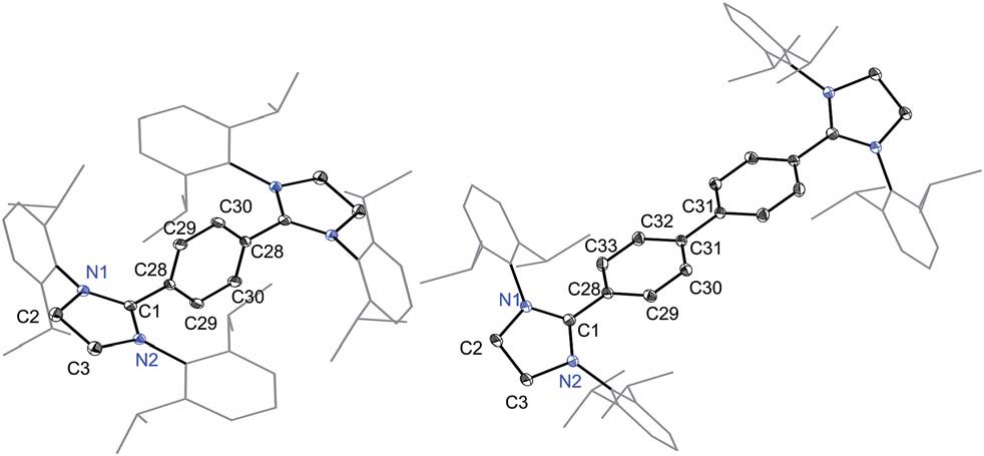
Solid-state molecular structures of **2** (left) and **3**. Hydrogen atoms and counter anions have been omitted for clarity. Selected bond lengths and angles for **2** and **3** are given in Table 1.

The direct two-fold carbenylation of 1,4-diiodobenzene and 4,4′-dibromobiphenyl with SIPr (**1**) under Ni-catalysis gave the starting materials **2** and **3**, respectively (Scheme 1).^16^ Compounds **2** and **3** are off-white solids and have been characterized by NMR spectroscopy and mass spectrometry. They exhibited characteristic ^1^H and ^13^C NMR resonances expected for C2-arylated 1,3-imidazolinium salts.^15^,^16^ Molecular structures of **2** and **3** have been determined by single crystal X-ray diffraction studies (Fig. 2). The metric parameters of **2** and **3** (Table 1) are fully consistent with those of reported mono-C2-arylated 1,3-imidazolinium species.^15^

**Table 1:** Selected bond lengths (Å) and angles (°) determined by X-ray diffraction^*a*^ for **2–5**, **TH** and **CH**;^8^ and calculated (B3LYP/def2-SVP) for **4** and **5** in closed-shell (CS) and triplet (T) ground-state.^*b*^ Compound **4** crystallized with two molecules in the asymmetric unit, given is the average of both molecules

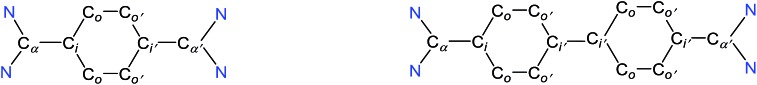								
Compound	N–C_α_	C_α_–C_i_	C_i_–C_o_	C_o_–C_o′_	N–C–N	C_i′_– C_i′_	ΣN	BLA^*c*^
**2**	1.326	1.478(2)	1.397	1.390(2)	113.2(1)	—	356.4	—
**TH**	—	1.381	1.449	1.346	—	—	—	0.10
**4**	1.404	1.371	1.451	1.349	108.2	—	349.4	0.10
CS	1.413	1.390	1.461	1.362	105.8	—	355.1	0.10
T	1.407	1.445	1.421	1.391	109.0		353.6	0.03
**3**	1.331	1.466(3)	1.395	1.383	111.5(2)	1.494(4)	355.5	—
**CH**	—	1.415	1.424	1.372	—	1.448	—	0.05
**5**	1.390	1.386(2)	1.443	1.359	107.9(1)	1.408(2)	347.3	0.08
CS	1.403	1.398	1.450	1.367	107.0	1.415	352.9	0.08
T	1.409	1.424	1.433	1.387	107.6	1.481	351.4	0.04

^*a*^The values for N–C_α_, C_α_–C_i_, C_i_–C_o_, C_o_–C_o′_, and N–C–N (except C_o_–C_o′_ of **2**, C_α_–C_i_ and N–C–N of **2**, **3**, and **5**) are averaged and given without estimated standard deviations (esd's).

^*b*^Optimized structures and further detail are provided in the ESI (see Fig. S6–S9).

^*c*^BLA = C_i_–C_o_ – C_o_–C_o′_.

**Fig. 3:**
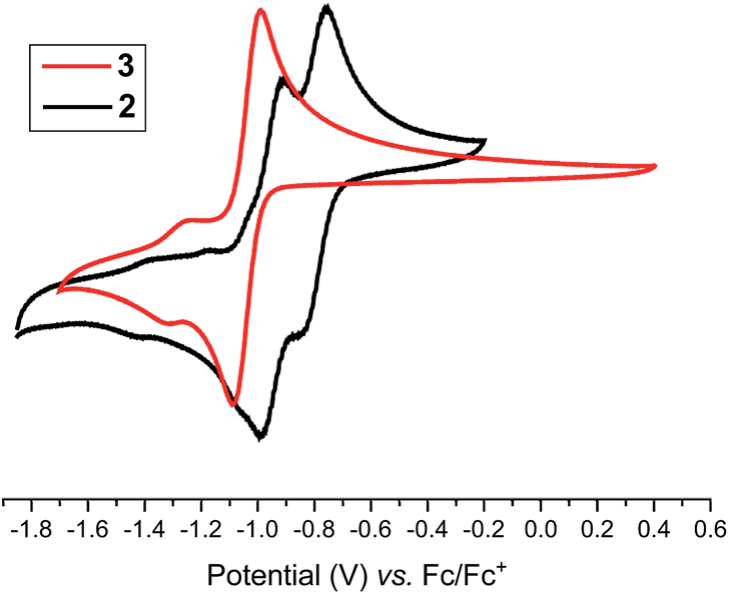
Cyclic voltammograms of **2** and **3** (CH_3_CN/*n*Bu_4_NPF_6_, 100 mV s^–1^, *vs.* Fc^+^/Fc).

**Scheme 2:**
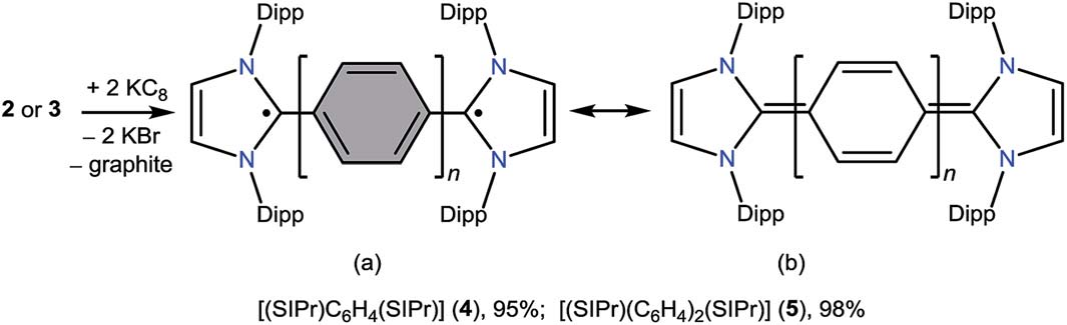
KC_8_ reduction of **2** and **3** to **4** and **5**.

Having the desired compounds in hand, we analyzed the electrochemical properties of **2** and **3** by cyclic voltammetry. Cyclic voltammograms (CVs) of **2** and **3** (Fig. 3) showed two sequential quasi-reversible redox processes, suggesting that both imidazolinium fragments are electrochemically coupled. The first reduction at *E*_1/2_ = –0.80 V (**2**) and *E*_1/2_ = –1.04 V (**3**) is most probably to form a radical cation, which undergoes second reduction at *E*_1/2_ = –0.95 V (**2**) and –1.28 V (**3**) to give the corresponding neutral species. Note, the reduction potential for **2** and **3** is significantly lower compared to the related C2-protonated imidazolinium salt (SIPr)HCl (*ca.* –2.3 V).^17^ Indeed, chemical reduction of **2** and **3** with KC_8_ at –78 °C in THF immediately resulted in the formation of a highly colored solution and a black precipitate (graphite) (Scheme 2). Upon usual workup, compound **4** (green) and **5** (blue) were isolated as air sensitive solids in an almost quantitative yield. Compounds **4** and **5** are indefinitely stable in solution as well as in solid state under an inert gas atmosphere. Single crystals of **4** and **5** suitable for X-ray diffraction were grown by storing a saturated *n*-hexane solution of each at –35 °C for 20 h.

**Fig. 4:**
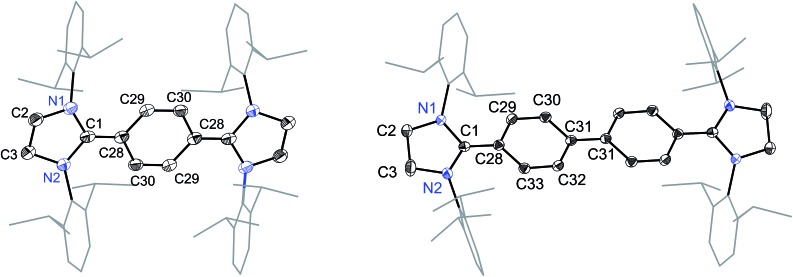
Solid-state molecular structures of [(SIPr)(C_6_H_4_)(SIPr)] (**4**) (left) and [(SIPr)(C_6_H_4_)_2_(SIPr)] (**5**) (right). Hydrogen atoms have been omitted for clarity. Selected bond lengths and angles for **4** and **5** are given in Table 1.

The solid-state molecular structures of **4** and **5** are shown in Fig. 4. The structural parameters of **4**, **5** and their precursors **2**, **3** along with **TH** and **CH** are given in Table 1. The C_α_–N (av. 1.328 Å), C_α_–C_i_ (av. 1.472 Å), and C_i_–C_o_/C_o_–C_o′_ (av. 1.391 Å) bond lengths of **2** and **3** are in accordance with those of mono C2-arylated imidazolinium salts.^15^ The C_α_–N bond length of **4** (av. 1.404 Å) and **5** (1.390 Å) are considerably longer, whereas C_α_–C_i_ bond lengths of **4** (av. 1.371 Å) and **5** (1.386(2) Å) are significantly shorter compared to those of **2** and **3**. The C_α_–C_i_ bond lengths of **4** and **5** are although longer than those in typical olefins (1.33–1.34 Å) and that of (IPr)CH_2_ (1.332(4) Å)^18^ but compare well with that of the vinylsilane IPrCH(SiHCl_2_) (1.379(2) Å)^19^ in which a considerable π-electron density has transferred to the silicon atom. In addition, the C_i_–C_o_ (1.442(2)–1.448(3) Å) and C_o_–C_o′_ (1.347(4)–1.360(2) Å) bond lengths of **4** and **5** are clearly distinguishable (*i.e.*, no longer benzenoid). While the bond length alternation (BLA) in **4** (0.10 Å) is similar to **TH** (0.10 Å), the same in **5** (0.08 Å) is intermediate of **4** and **CH** (0.05 Å).^8^ In addition, the C_i_–C_i′_ bond length of **5** (1.408 Å) is shorter compared to that of **CH** (1.448 Å). This indicates a higher quinoidal contribution to the resonance structure of **5** compared to **CH**, which is however lower in comparison with **4** and **TH**. This becomes also evident when the planarity of imidazolidine rings is compared with that of the bridging phenyl or diphenylene ring. The imidazolidine rings are puckered and feature the plane twist angle of 56.42(5)° in **2** and av. 8.28(10)° in **4** from the bridging phenyl ring, indicating a significant contribution of the quinoid resonance form in the later. As expected, the plane twist angle in **5** (17.56(5)°) is also smaller compared to that of **3** (46.41(9)°), but it is almost twice that of the **4** (8.28(10)°). This also emphasizes the diminished quinoidal contribution to the resonance structure of **5** compared to **4**.

**Fig. 5:**
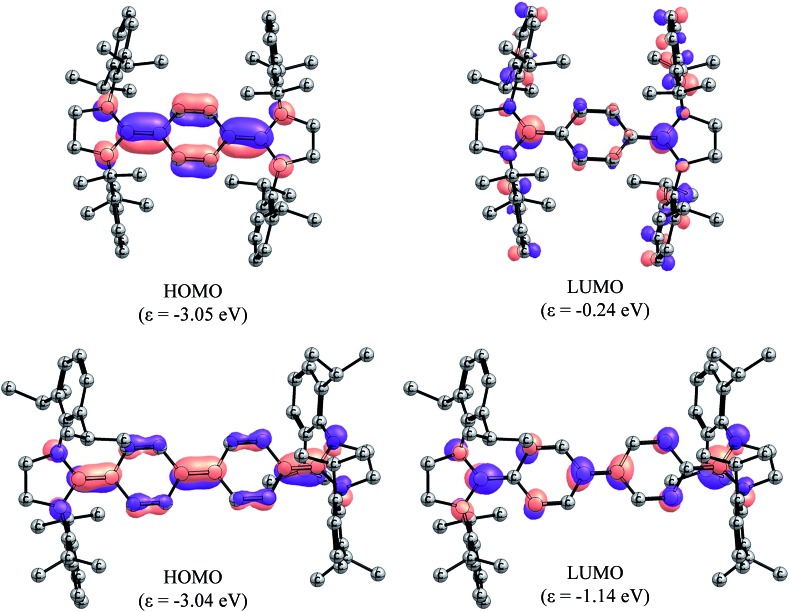
Molecular orbitals (isovalue 0.05) of the CS singlet state species of **4** (top) and **5** (bottom) calculated at B3LYP/def2-TZVPP level of theory. Hydrogens have been omitted for clarity.

In order to gain deeper insight into the electronic structures of **4** and **5**, DFT calculations were performed at the B3LYP/def2-SVP and BH&HLYP/def2-SVP level of theory (see ESI† for details). The frontier molecular orbitals (FMOs) of **4** and **5** are shown in Fig. 5. Geometry optimizations were performed considering three electronic states namely, closed-shell singlet (CS), open-shell singlet (OS), and triplet (T) states (Table 1). As can be seen, the calculated CS singlet state structures are in a better agreement with X-ray structures. The pyramidalization of the nitrogen atom of imidazolidine ring can also be accounted for the quinoidal character of **4** and **5**. The nitrogen atoms in **2** and **3** are almost planar (the sum of the angles at the nitrogen atom ΣN = 355–356°), whereas one of the nitrogen atoms of NHC units in **4** (ΣN = 349.4°) and **5** (ΣN = 347.3°) is pyramidalized. This becomes also obvious when ΣN of **4** and **5** calculated for CS and T states are compared. The OS calculations were carried out within the Noodleman's broken-symmetry (BS) formalism.^20^ All BS computations showed no spin-contamination collapsing on the CS species. In the case of **5**, a broken symmetry singlet wave function –2.3 kcal mol^–1^ lower compared to the CS singlet species is recognized (Table 2).

**Table 2:** Calculated energy (kcal mol^–1^) of closed-shell (CS), open-shell (OS), and triplet (T) ground-state and diradical character (*y*) of **4** and **5**

Method	Compound	CS	OS	T	*y*
B3LYP/def2-TZVPP	**4**	0.0	0.0	+29.1	0.00
	**5**	0.0	0.0	+10.7	0.43
BH&HLYP/def2-TZVPP	**4**	0.0	0.0	+27.8	0.0
	**5**	0.0	–2.3	+4.8	0.35

An improvement of the electronic energies of each species was performed at B3LYP/def2-TZVPP, PBE0/def2-TZVPP, BH&HLYP/def2-TZVPP, and M06-2X/def2-TZVPP levels of theory (see the ESI†). All these methods predicted that for **4** the triplet form is 29.1 kcal mol^–1^ (B3LYP), 28.7 kcal mol^–1^ (PBE0), 27.8 kcal mol^–1^ (BH&HLYP), or 32.0 kcal mol^–1^ (M06-2X) higher in energy (Table T3 in the ESI†). The computed singlet–triplet energy difference Δ*E*_S–T_ for **5** is 10.7 kcal mol^–1^ (B3LYP), 9.5 kcal mol^–1^ (PBE0), 4.8 kcal mol^–1^ (BH&HLYP), or 9.8 kcal mol^–1^ (M06-2X) (Table T4 in the ESI†). It should however be noted that among all the functionals used, BH&HLYP was the only one that recognized a singlet broken symmetry state (Table 2). We also calculated the diradical character (*y*) of **4** and **5** as described by Nakano (*y* = 0 for the closed-shell and *y* = 1 for the pure singlet diradical).^21^ Compound **4** is a perfectly closed-shell species while compound **5** has 43% diradical character (Table 2). The estimated values are however lower than those reported for the **TH** (0.31) and **CH** (0.72) HCs.6*c*

We also performed CASSCF(2,2)+NEVPT2/def2-SVP calculations on model systems **4^Me^** and **5^Me^**, where the 2,6-iPr_2_C_6_H_3_ groups of compounds **4** and **5** were replaced by methyl groups (See the ESI, Tables T5–T7†). These calculation led to a CI (configuration interaction) vector having coefficients of 0.96 for the closed-shell 2,0 configuration, 0.0 for the 1,1 configuration, and 0.3 for the 0,2 configuration. The diradical index computed according to Neese *et al.*^22^ suggest that **4^Me^** and **5^Me^** are closed-shell species with 7.0% and 7.8% diradicaloid character, respectively. Indeed, the occupation of HOMO is in both case 1.92 electrons, while LUMO has 0.07 electrons.

From the above discussion, it becomes clear that the closed-shell singlet is the ground state for **4** and **5**. Indeed, a carefully prepared sample of **4** was EPR silent and exhibit well resolved ^1^H and ^13^C NMR resonances in the expected region (see Plots P8–P10 in the ESI†). Under similar conditions, the sample prepared by dissolving crystals of **5** exhibit a doublet EPR signal (Fig. S5 in the ESI†), which corresponds to a mono-radical species. This observation is in line with the baffling controversy over the magnetic properties of **CH**, the so-called “diradical paradox”.7*a* Similar observations have been also made in many other closed-shell compounds reported recently.11a,^14^,^23^ Baumgarten6*c* addressed this issue in a very recent report that concludes that the weak intensity triplet expected for **CH** can be masked under even ≈0.1% of mono-radical impurity.

Compound **4** showed an intense absorption with maximum (*λ*_max_) at 431 nm along with a weak absorption at 619 nm (Fig. S3†). Compound **5** exhibited *λ*_max_ at 618 nm with a shoulder at 580 nm (Fig. S4†). We carried out time-dependent TD-PCM(THF)-B3LYP/def2-SVP calculations for the assignment of the UV-visible spectral bands. The results are summarized in Tables T8 and T9 (see the ESI†). The results for **4** in the closed-shell configuration gave three main excitations at 256 nm, 350 nm, and 539 nm with the oscillator strengths of 0.092, 1.386, and 0.066, respectively. The main absorption corresponds to the HOMO → LUMO + 8 transition (Fig. S13†). For compound **5**, the absorption bands are red shifted and the oscillator strengths are stronger. The main absorption is due to the HOMO → LUMO transition and occurs at 441 nm with the oscillator strength of 3.296 (Fig. 5).

## Experimental

### Materials and methods

All syntheses and manipulations were performed under an inert gas (Ar or N_2_) atmosphere using standard Schlenk techniques and an MBraun LABmaster Pro glovebox. THF, *n*-hexane, and *o*-xylene were dried over NaK, distilled prior to use, and stored over 3 Å molecular sieve. Melting points were measured using a Büchi B-545 melting point apparatus. NMR spectra were recorded using a Bruker Avance III 500 or a Bruker Avance III 500HD NMR spectrometer. Chemical shifts are given in *δ* ppm and are referenced to the solvent residual peaks:^24^ DMSO-*d*_6_ (^1^H, *δ* = 2.50 ppm and ^13^C, *δ* = 39.52 ppm), C_6_D_6_ (^1^H, *δ* = 7.16 ppm and ^13^C, *δ* = 128.06 ppm). ESI mass spectra were recorded with a Bruker Esquire 3000 spectrometer. UV-visible spectra were recorded using a ThermoFisher Evolution 300 spectrophotometer.

### Synthetic procedures of **2–5**

Compounds **2** and **3** were prepared by the direct C2-arylation of SIPr (**1**) with 1,4-diiodobenzene and 4,4′-dibromobiphenyl under nickel catalysis.^16^,^25^

### [(SIPr)C_6_H_4_(SIPr)](I)_2_ (**2**)

To a Schlenk flask containing SIPr (**1**) (1.76 g, 4.50 mmol), 1,4-diiodobenzene (0.70 g, 2.21 mmol), and Ni(cod)_2_ (87 mg, 0.32 mmol) was added 40 mL *o*-xylene. The resulting reaction mixture was heated under reflux for 4 h and then brought to room temperature. Filtration through a G4 porosity frit afforded an off-white solid, which was washed with (2 × 10 mL) toluene and dried under vacuum. Compound **2** was obtained as a white solid in 81% (1.90 g) yield. Mp: 348–350 °C (dec.). Elemental analysis (%) calcd for **2**, C_60_H_80_I_2_N_4_ (1111): C 64.86; H 7.26; N 5.04; found: C 64.54, H 7.22, N 4.79. ^1^H NMR (500 MHz, DMSO-*d*_6_, 25 °C): *δ* = 0.68 (d, *J* = 6.7 Hz, 24H, CH(CH_3_)_2_), 1.23 (d, *J* = 6.5 Hz, 24H, CH(CH_3_)_2_), 2.87 (sept, *J* = 6.8 Hz, 8H, CH(CH_3_)_2_), 4.55 (s, 8H, NCH_2_), 6.83 (s, 4H, C_6_H_4_), 7.22 (d, *J* = 7.8 Hz, 8H, *m*-C_6_H_3_), 7.42 (t, *J* = 7.8 Hz, 4H, *p*-C_6_H_3_) ppm. ^13^C NMR (125 MHz, DMSO-*d*_6_, 25 °C): *δ* = 22.5, 26.0, 28.2, 53.6, 124.8, 125.3, 125.5, 125.7, 128.7, 129.1, 129.4, 131.4, 136.0, 145.1, 146.1, 164.2 ppm. MS (ESI pos.): *m*/*z* = 428.67 [**2** – 2I]^++^.

### [(SIPr)(C_6_H_4_)_2_(SIPr)](Br)_2_ (**3**)

Compound **3** was prepared by adopting a similar method as descried for **2** using SIPr (**1**) (1.50 g, 3.84 mmol), 4,4′-dibromobiphenyl (0.59 g, 1.89 mmol), and Ni(cod)_2_ (75 mg, 0.25 mmol). Yield: 1.47 g, 71%. Mp: 358–360 °C. Elemental analysis (%) calcd for **3**, C_66_H_84_Br_2_N_4_ (1093): C 72.51; H 7.75; N 5.12; found: C 71.75, H 7.47, N 4.88. ^1^H NMR (500 MHz, DMSO-*d*_6_, 25 °C): *δ* = 0.96 (d, *J* = 6.7 Hz, 24H, CH(CH_3_)_2_), 1.31 (d, *J* = 6.5 Hz, 24H, CH(CH_3_)_2_), 3.06 (sept, *J* = 6.6 Hz, 8H, CH(CH_3_)_2_), 4.56 (s, 8H, NCH_2_), 6.97 (d, *J* = 8.6 Hz, 4H, C_6_H_4_), 7.34 (d, *J* = 7.7 Hz, 8H, *m*-C_6_H_3_), 7.46 (t, *J* = 7.7 Hz, 4H, *p*-C_6_H_3_), 7.72 (d, *J* = 8.5 Hz, 4H, C_6_H_4_) ppm. ^13^C NMR (125 MHz, DMSO-*d*_6_, 25 °C): *δ* = 22.8, 25.7, 28.40, 53.5, 125.6, 127.3, 129.6, 130.6, 131.2, 145.3, 165.3 ppm. MS (ESI pos.): *m*/*z* = 466.4 [**3** – 2Br]^++^.

### [(SIPr)C_6_H_4_(SIPr)] (**4**)

To a Schlenk flask containing **2** (250 mg, 0.25 mmol) and KC_8_ (74 mg, 0.55 mmol) was added 15 mL pre-cooled (–78 °C) THF, which immediately led to the formation of a green suspension. The reaction mixture was brought to room temperature and further stirred for 2 h. Filtration through a plug of Celite afforded a green solution. The volatiles were removed under vacuum and the remaining green residue was extracted with *n*-hexane and stored at –24 °C to obtain **4** as a crystalline solid. Yield: 204 mg, 95%; mp 181 °C (dec.). Elemental analysis (%) calcd for **4**, C_60_H_80_N_4_ (857): C 84.06; H 9.41; N 6.54; found: C 83.36, H 9.13, N 6.32. UV-vis THF, *λ* (nm) (ε (M^–1^ cm^–1^)): 399 (15 906), 431 (17 389), 578 (2020), 619 (2791), 908 (486). MS (ESI pos.): *m*/*z* = 428.3 [**4** + 2H]^+^. ^1^H NMR (500 MHz, C_6_D_6_, 25 °C): *δ* = 1.21 (d, *J* = 6.8 Hz, 24H, HC(CH_3_)_2_), 1.24 (d, *J* = 6.8 Hz, 24H, HC(CH_3_)_2_), 3.37 (s, 8H, NCH_2_), 3.49 (sept, *J* = 6.8 Hz, 8H, HC(CH_3_)_2_), 5.04 (s, 4H, C_6_H_4_), 7.02 (d, *J* = 7.5 Hz, 8H, *m*-C_6_H_3_), 7.10 (t, *J* = 7.4 Hz, 4H, *p*-C_6_H_3_) ppm.^13^C NMR (125 MHz, C_6_D_6_, 25 °C): *δ* = 23.7, 25.2 (HC(CH_3_)_2_), 28.5 (HC(CH_3_)_2_), 53.0 (CH_2_N), 117.6 (C_6_H_4_), 124.3 (*p*-C_6_H_3_), 127.4 (*m*-C_6_H_3_), 128.1, 128.4, 134.9, 141.5, 147.1 (*o*-/*ipso*-C_6_H_3,_*p*-C_6_H_4_) ppm.

### [(SIPr)(C_6_H_4_)_2_(SIPr)] (**5**)

Compound **5** was prepared by employing a similar method as described above for **4** using **3** (800 mg, 0.73 mmol) and KC_8_ (198 mg, 1.46 mmol) in THF (20 mL) as a deep blue solid. Yield: 670 mg, 98%; mp 201 °C (dec.). Elemental analysis (%) calcd for **5**, C_66_H_84_N_4_ (933): C 84.93; H 9.07; N 6.00; found: C 84.55, H 8.77, N 5.68. UV-vis THF, *λ* (nm) (*ε* (M^–1^ cm^–1^)): 580 (14 860), 618 (20 940), 911 (520). MS (ESI pos.): *m*/*z* = 466.3 [**5** + 2H]^+^.

### X-ray crystallography

Solid-state molecular structures of **2** (Fig. S6†), **3** (Fig. S7†), and crystallographic details of **2–5** (Tables T1 and T2) are given in the ESI.† CCDC ; 1826567 (**2**), ; 1826568 (**3**), ; 1826569 (**4**), and ; 1826570 (**5**) contain the supplementary crystallographic data for this paper.†

### Computational calculations

Geometry optimizations were performed using Gaussian 09 (ref. 26) together with TurboMole V6.5.^27^ All geometry optimizations were computed using the functional B3LYP^28^ and BH&HLYP^29^ in combination with the def2-SVP basis set.^30^ The stationary points were located with the Berny algorithm^31^ using redundant internal coordinates. Analytical Hessians were computed to determine the nature of stationary points. The improvements in the electronic energies were carried out by computing single points on the B3LYP/def2-SVP geometries at the B3LYP/def2-TZVPP, BH&HLYP/def2-TZVPP, PBE0/def2-TZVPP^32^ and M06-2X/def2-TZVPP^33^ levels of theory. Further calculation details on optimized molecular structures, relative free energies, frontier molecular orbital analyses, and UV-visible spectra are provided in the ESI.†

## Conclusions

In conclusion, we have presented the synthesis and characterization of the first Kekulé diradicaloid compounds **4** and **5** derived from a classical NHC **1**. Compounds **4** and **5** may be considered as NHC analogues of Thiele's and Chichibabin's hydrocarbons. Remarkably, the precursor compounds **2** and **3** are readily accessible by the double carbenylation of *para*-dihaloarenes with a commercially available NHC **1** using Ni-catalysis. Experimental and computational studies fully corroborate the closed-shell singlet ground state of **4** and **5**. The results demonstrate that by using appropriate linkers, synthetic access to non-Kekulé diradicals and poly-radicals derived from NHCs seems feasible and therefore is worth pursuing.

## Conflicts of interest

There are no conflicts to declare

## Supplementary Material

Supplementary information

Crystal structure data
