# Supplementary material for: Kekulé diradicaloids derived from a classical N-heterocyclic carbene
Source: Chem Sci. 2018 Apr 24;9(22):4970–6. doi: 10.1039/c8sc01209a (PMC5989652; doi:10.1039/c8sc01209a)
Supplement: Supplementary file 1 [file SC-009-C8SC01209A-s001.pdf]

Electronic Supplementary Material (ESI) for Chemical Science.
This journal is © The Royal Society of Chemistry 2018

Kekulé Diradicaloids Derived from a Classical N-Heterocyclic Carbene

Dennis Rottschäfer,[a] Beate Neumann,[a] Hans-Georg Stammler,[a] Diego M. Andrada,[b] and Rajendra S. Ghadwal*,[a]

[a]Anorganische Molekülchemie und Katalyse, Lehrstuhl für Anorganische Chemie und Strukturchemie, Centrum für Molekulare Materialien, Fakultät für Chemie, Universität Bielefeld, Universitätsstr. 25, D-33615 Bielefeld, Germany

[b]Allgemeine und Anorganische Chemie, Universität des Saarlandes, Campus C4.1, D-66123 Saarbrücken, Germany

Email: rghadwal@uni-bielefeld.de

Contents:

NMR Plots of **2**, **3** and **4** (pages 3-7)
Cyclic Voltammetry (pages 8 and 9)
UV-visible Spectroscopy (page 10)
EPR Spectroscopy (page 11)
X-Ray Diffraction Studies (pages 12-15)
Computational Studies (pages 16-55)
References (page 56)

NMR plots of compounds 2, 3, and 4

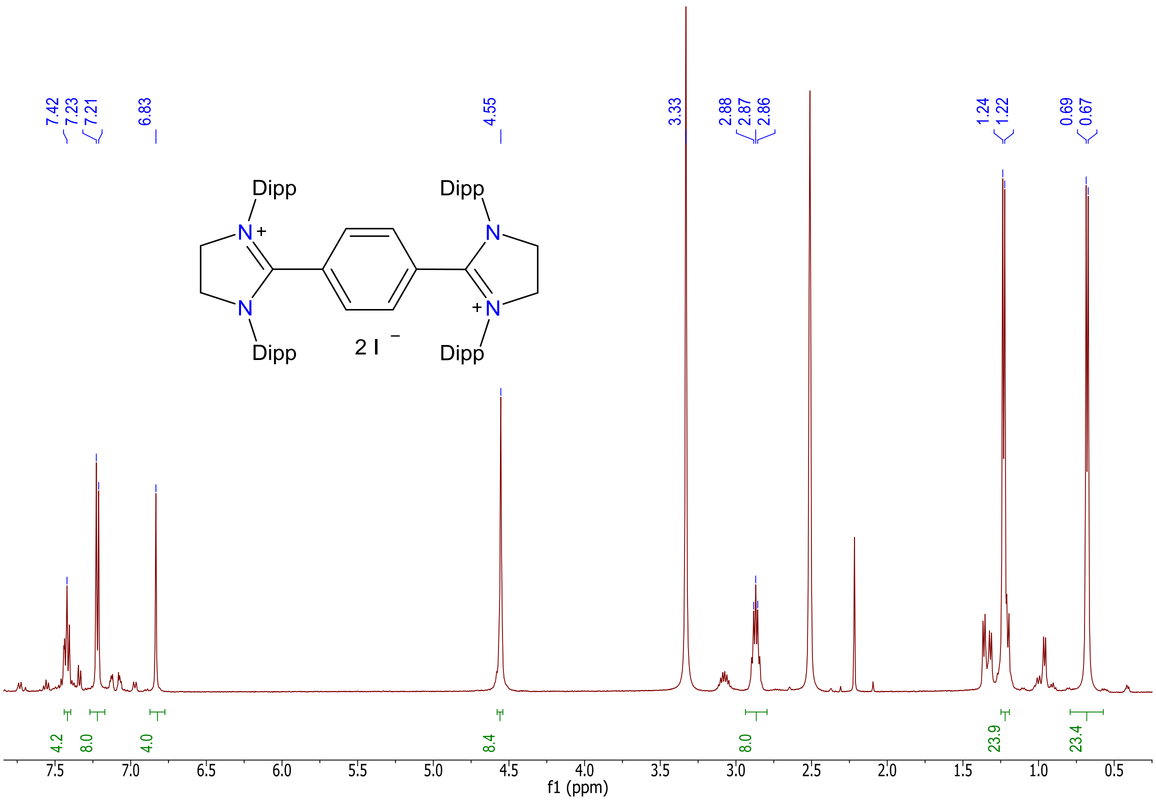

Plot P1. ^{1}H NMR spectrum (in DMSO-d_6) of $[(SIPr)(C_6H_4)(SIPr)](I)_2$ (**2**).

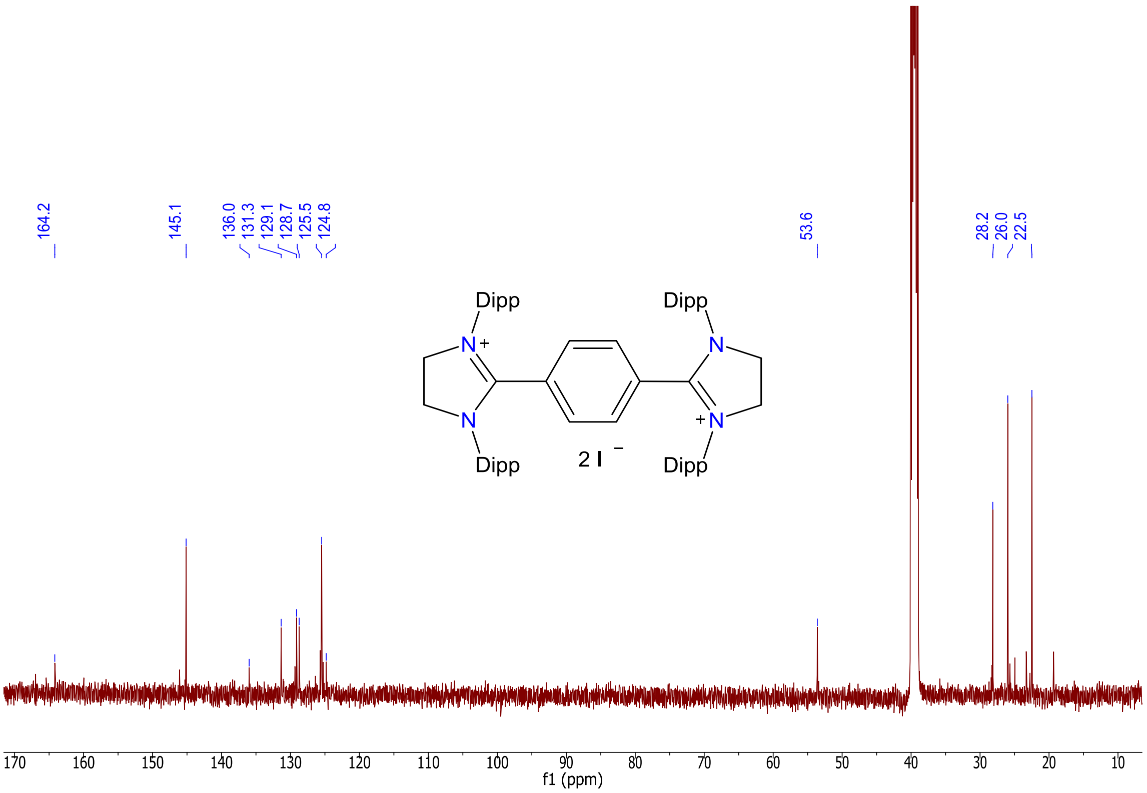

Plot P2. $^{13}C\{^1H\}$ NMR spectrum (in DMSO-d_6) of $[(SIPr)(C_6H_4)(SIPr)](I)_2$ (**2**).

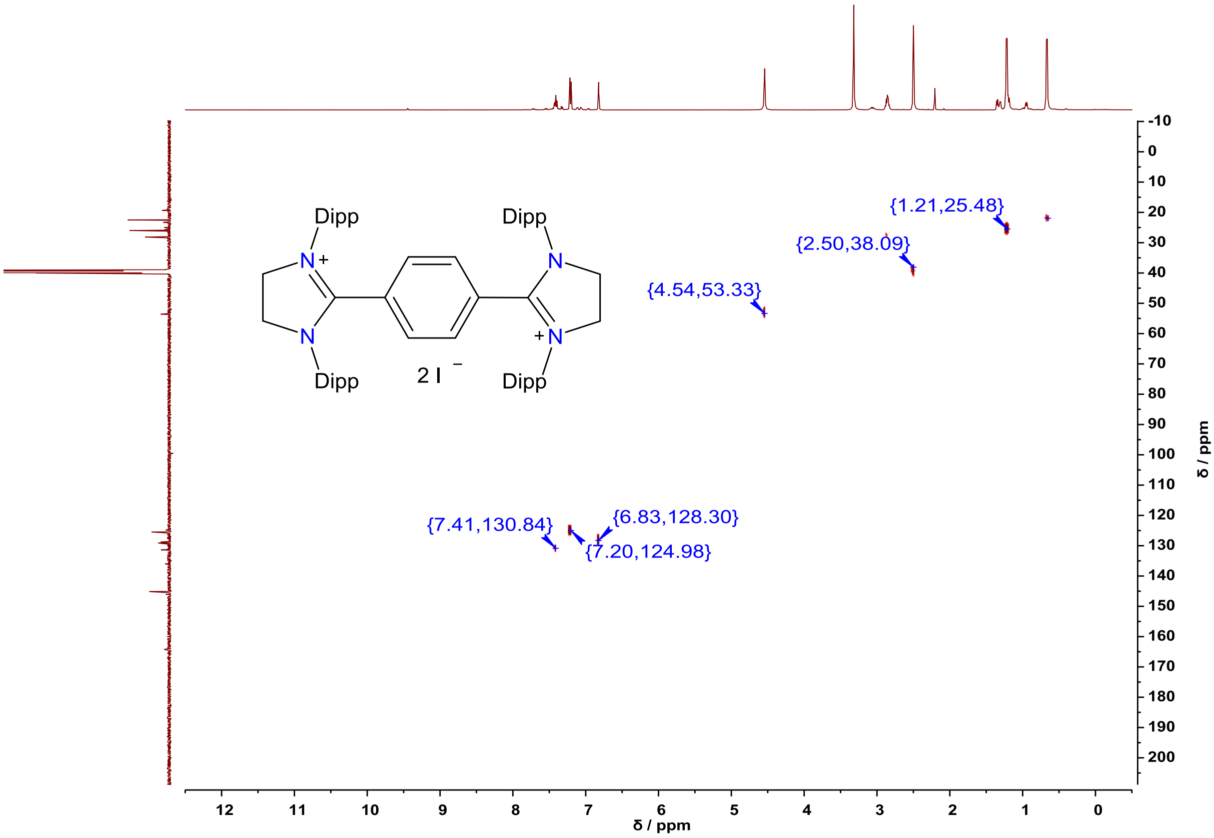

Plot P3. ^{1}H-^{13}C HMQC NMR spectrum (in DMSO-d_6) of $[(SIPr)(C_6H_4)(SIPr)](I)_2$ (**2**).

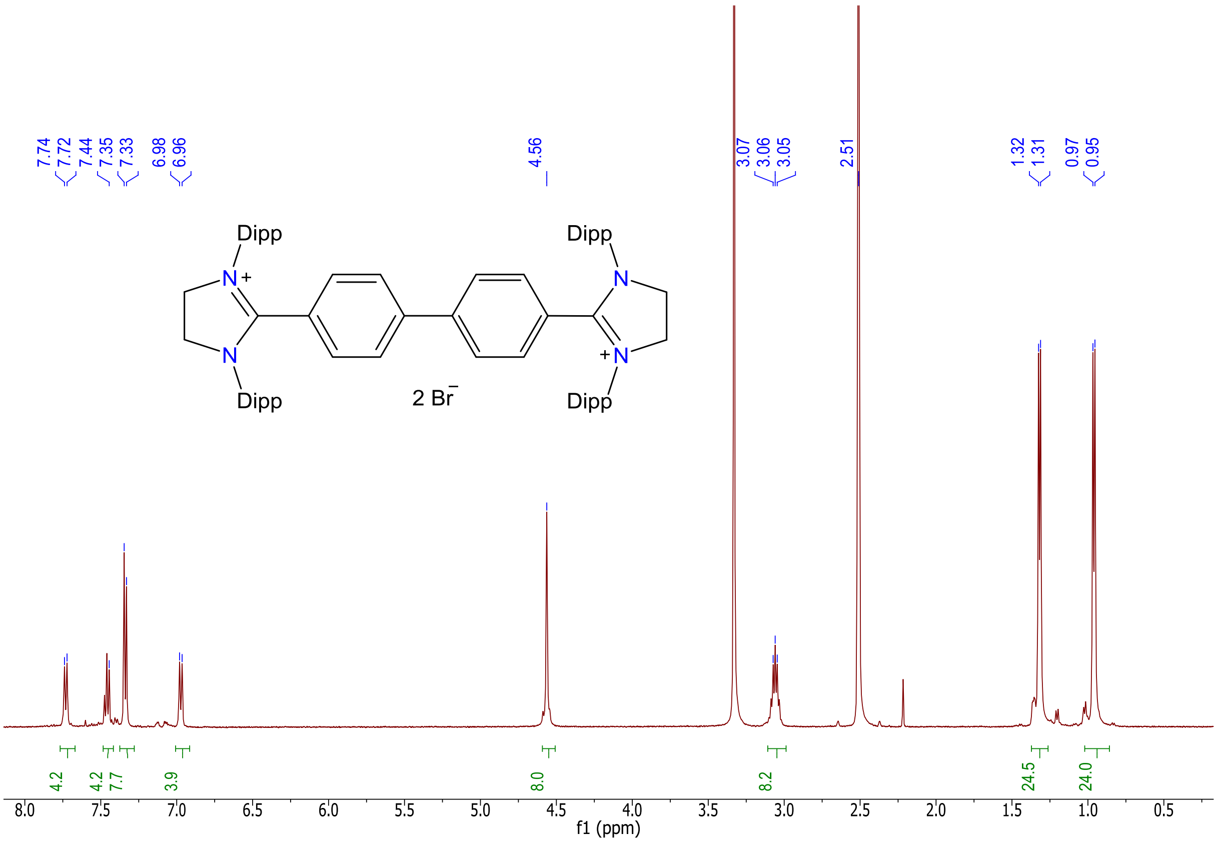

Plot P4. ^{1}H NMR spectrum (in DMSO-d_6) of $[(SIPr)(C_6H_4)_2(SIPr)](Br)_2$ (**3**).

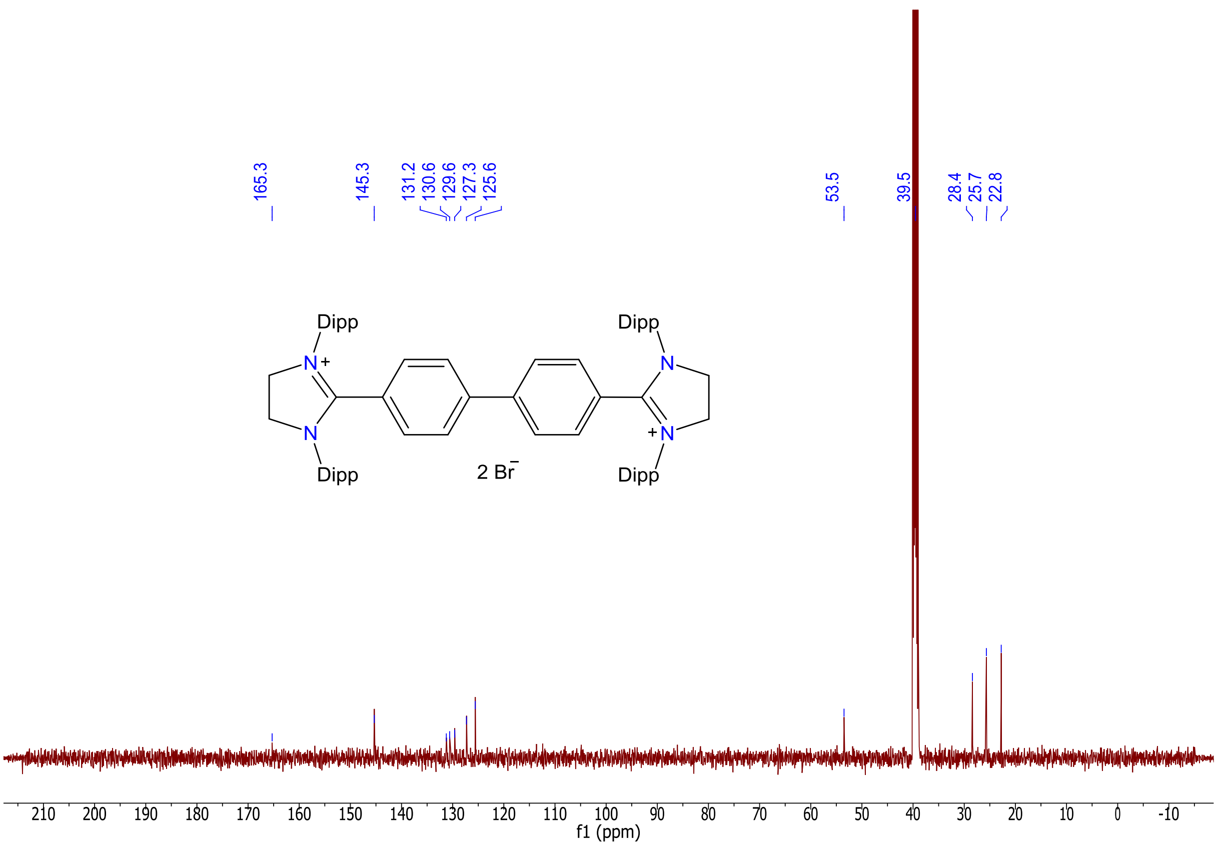

Plot P5. $^{13}C\{^{1}H\}$ NMR spectrum (in DMSO-d_6) of $[(SIPr)(C_6H_4)_2(SIPr)](Br)_2$ (**3**).

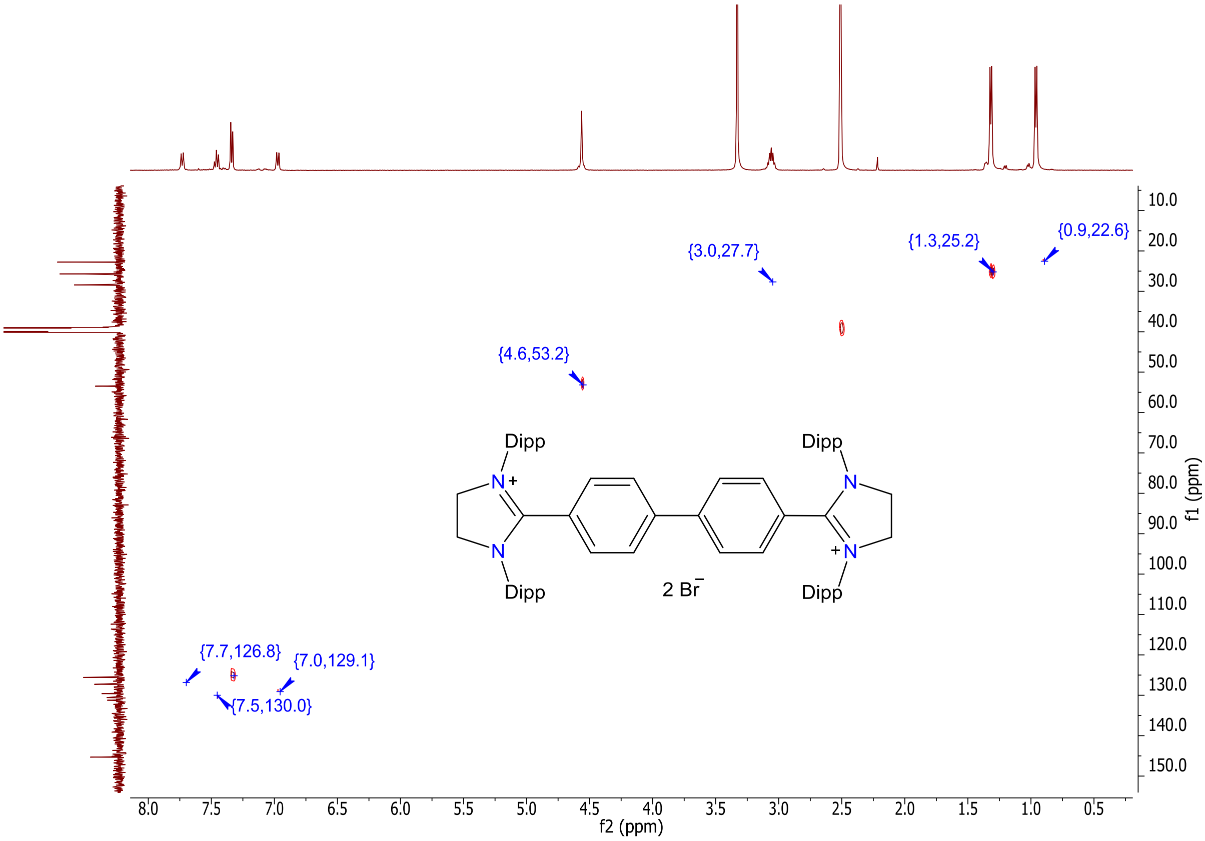

Plot P6. ^{1}H-^{13}C HMQC NMR spectrum (in DMSO-d_6) of $[(SIPr)(C_6H_4)_2(SIPr)](Br)_2$ (**3**).

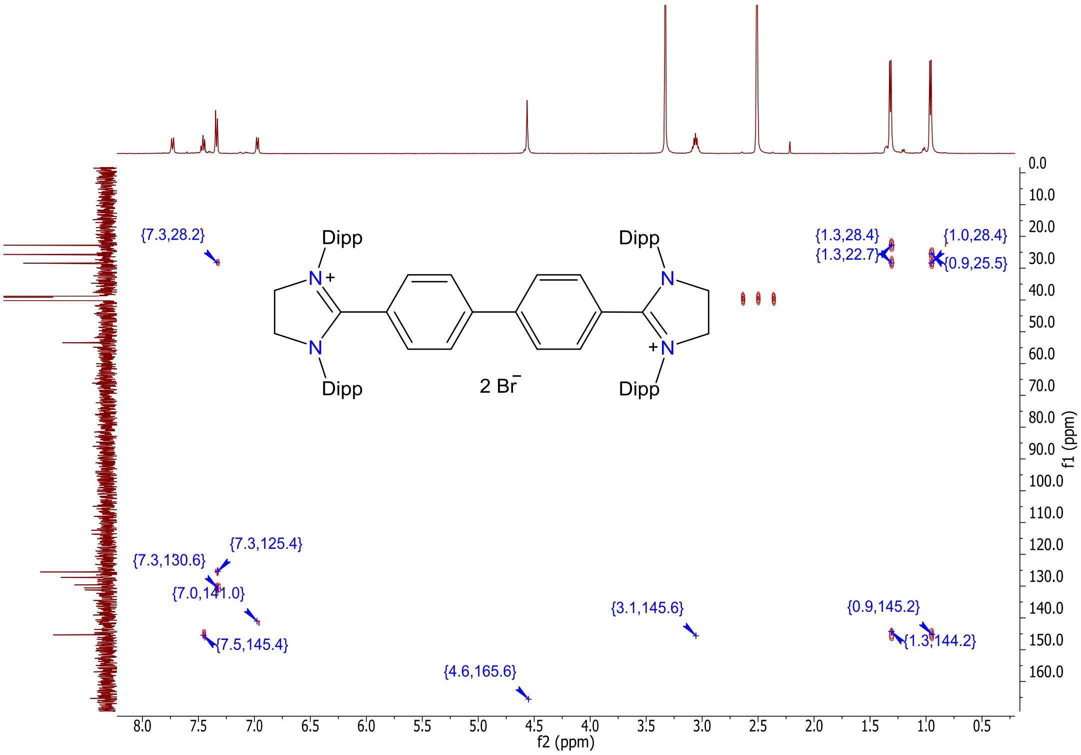

Plot P7. ^{1}H-^{13}C HMBC NMR spectrum (in DMSO-d_6) of [(SIPr)(C_6H_4)$_2$(SIPr)]$(Br)_2$ (**3**).

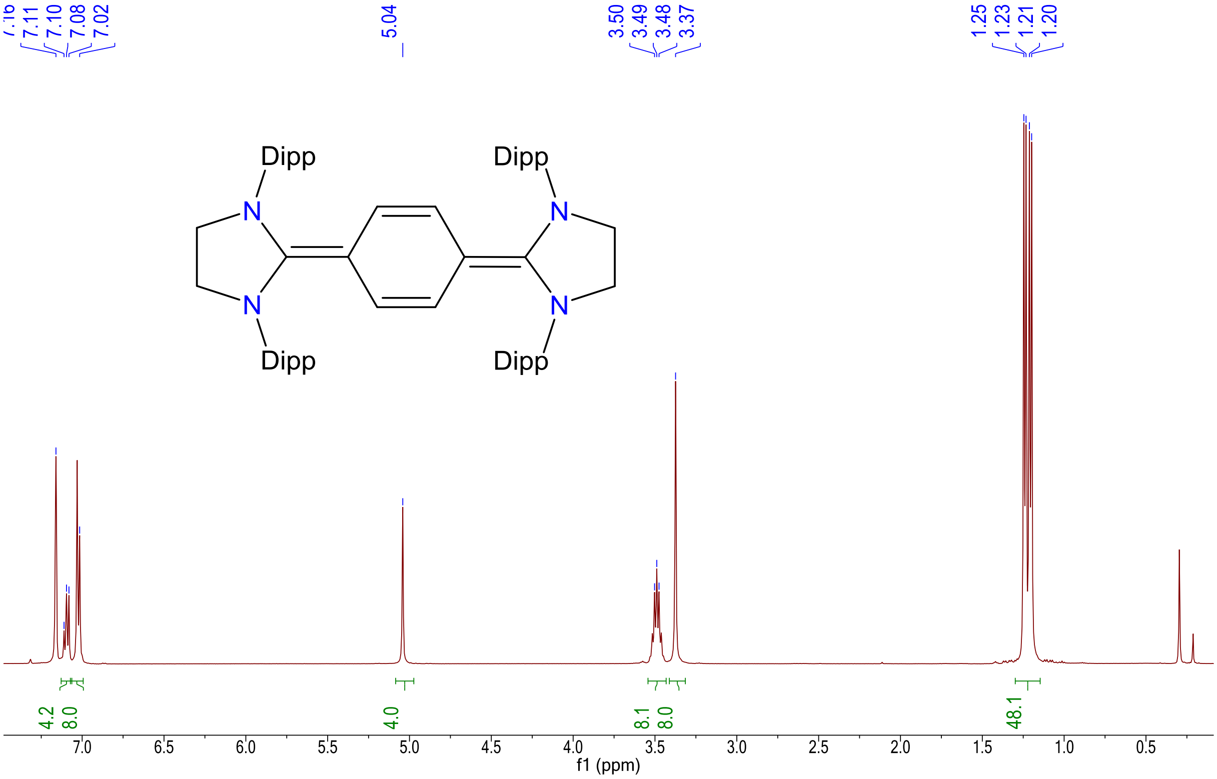

Plot P8. ^{1}H NMR spectrum (in C_6D_6) of [(SIPr)(C_6H_4)(SIPr)] (**4**).

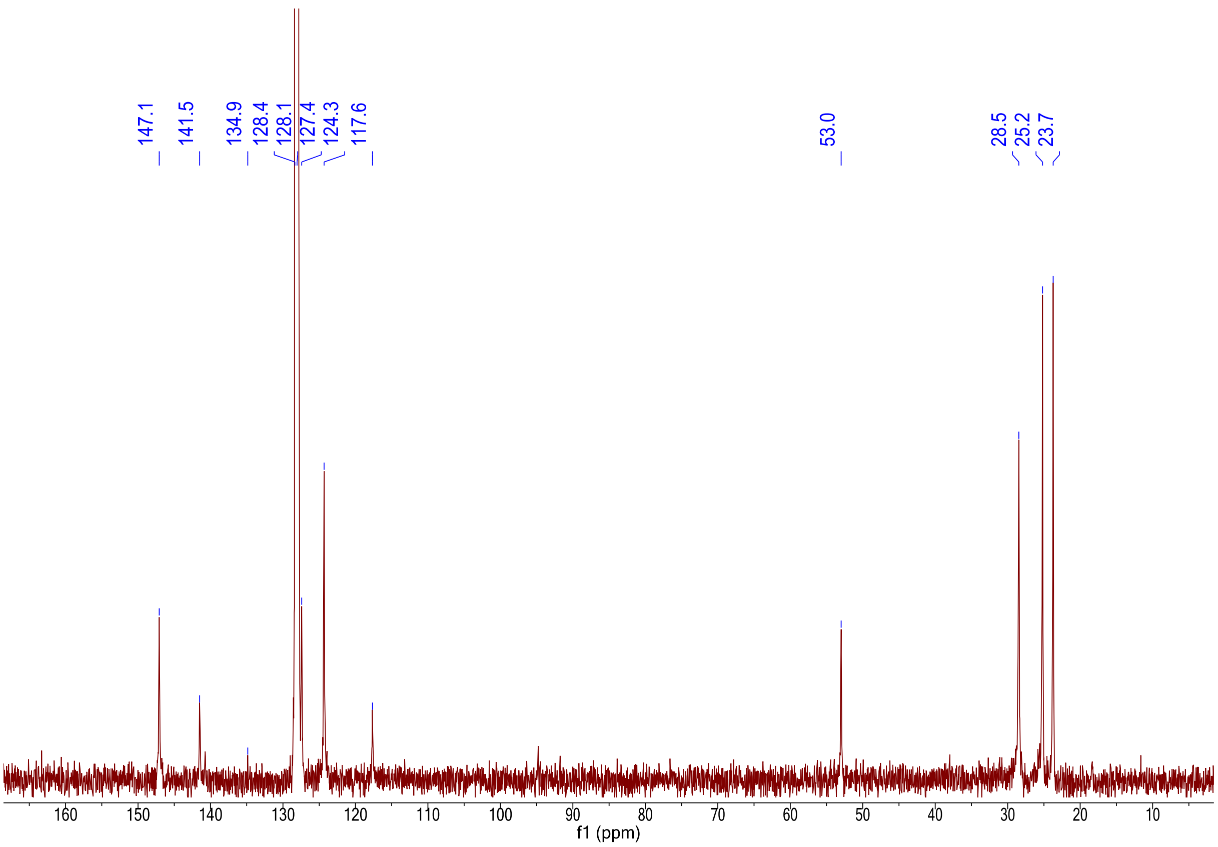

Plot P9. ^{13}C NMR spectrum (in C_6D_6) of [(SIPr)(C_6H_4)(SIPr)] (**4**).

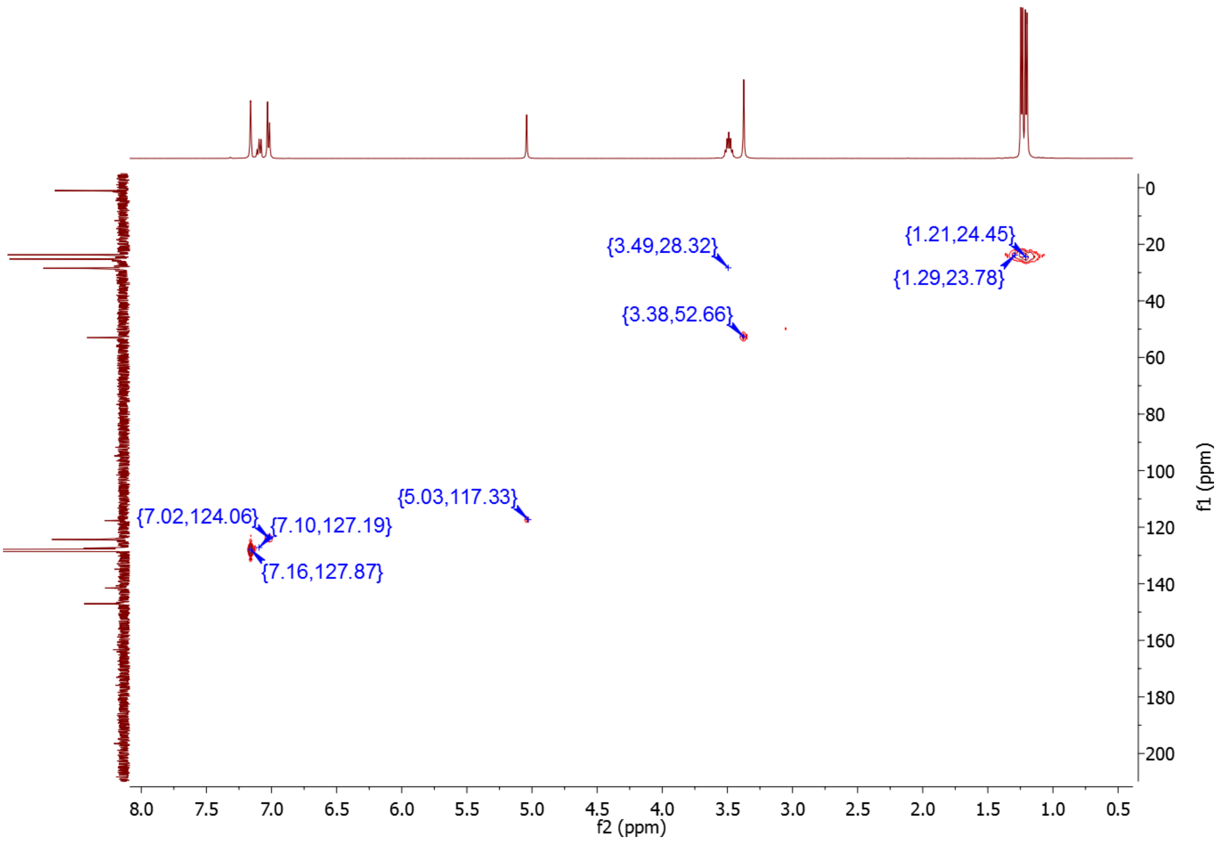

Plot P10. ^{1}H-^{13}C HMQC NMR spectrum (in C_6D_6) of [(SIPr)(C_6H_4)(SIPr)] (**4**).

Cyclic Voltammetry

Cyclic voltammetry (CV) experiments were carried out using a PGSTAT 101 electrochemical workstation (METROHM). All experiments were carried out under an atmosphere of argon in degassed and anhydrous acetonitrile solution containing $^{t}Bu_4NPF_6$ (0.1 M) at a scan rate of 50 mV s^{-1} up to 500 mV s^{-1}. The setup consisted of a glassy carbon working electrode (surface area = 0.04 cm^2), a glassy carbon counter electrode, and a silver wire immersed in a saturated LiCl solution in EtOH and 0.1 M $Bu^{t}_4NPF_6$ solution in acetonitrile as the reference electrode. The recorded voltammograms were referenced to the internal standard Fc/Fc^+ (ferrocene/ferrocenium) couple.

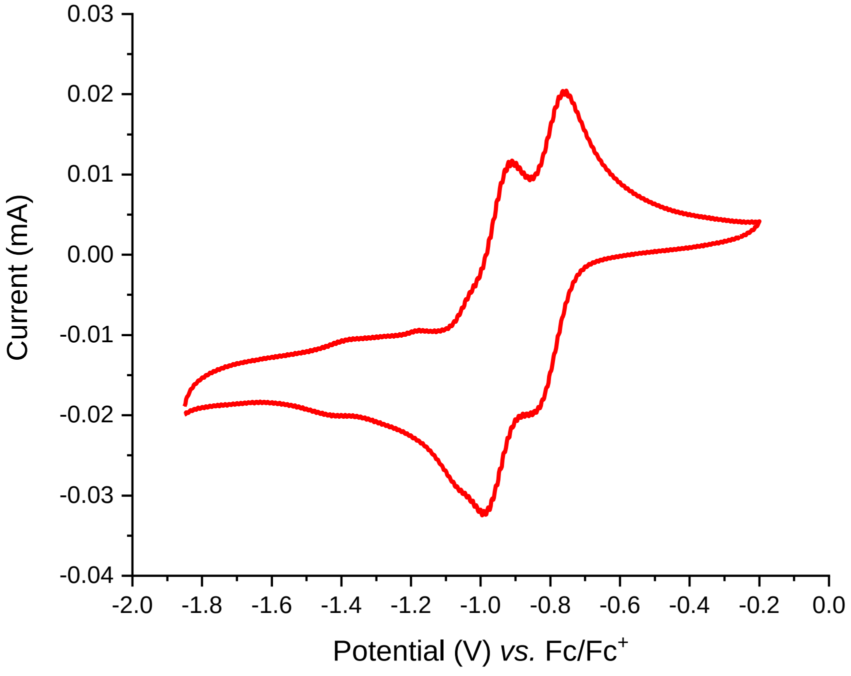

Figure S1. Cyclic voltammogramm of **2** recorded at 100 mV s^{-1}.

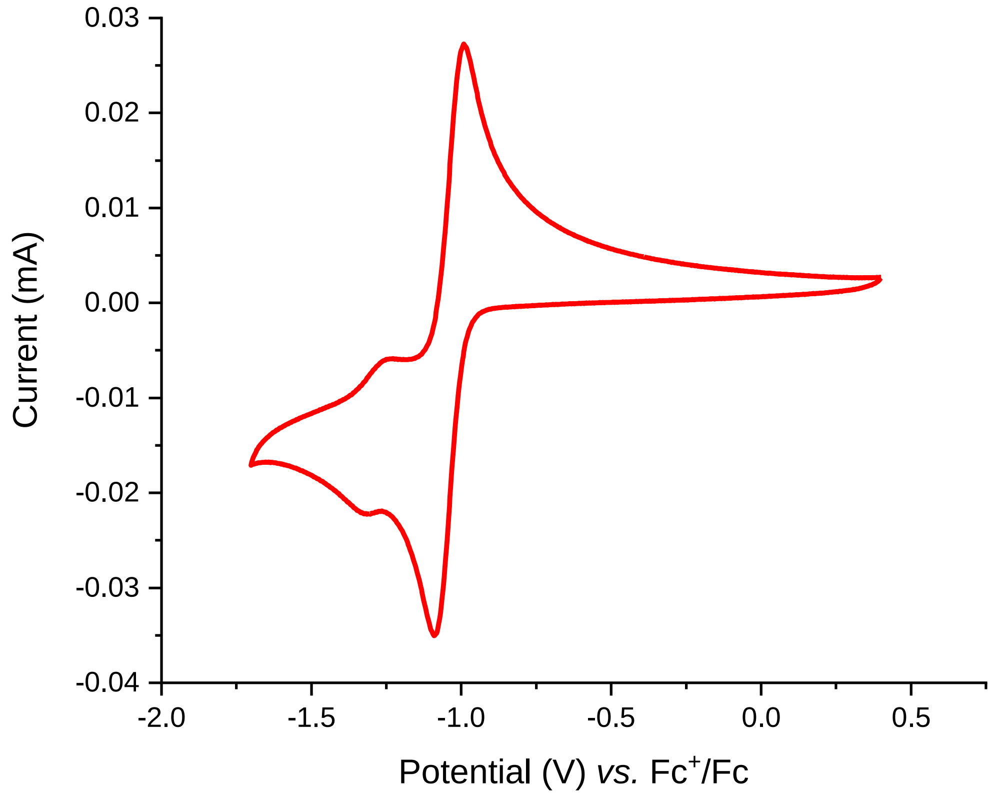

Figure S2. Cyclic voltammogramm of **3** recorded at 100 mV s^{-1}.

UV-visible Spectroscopy

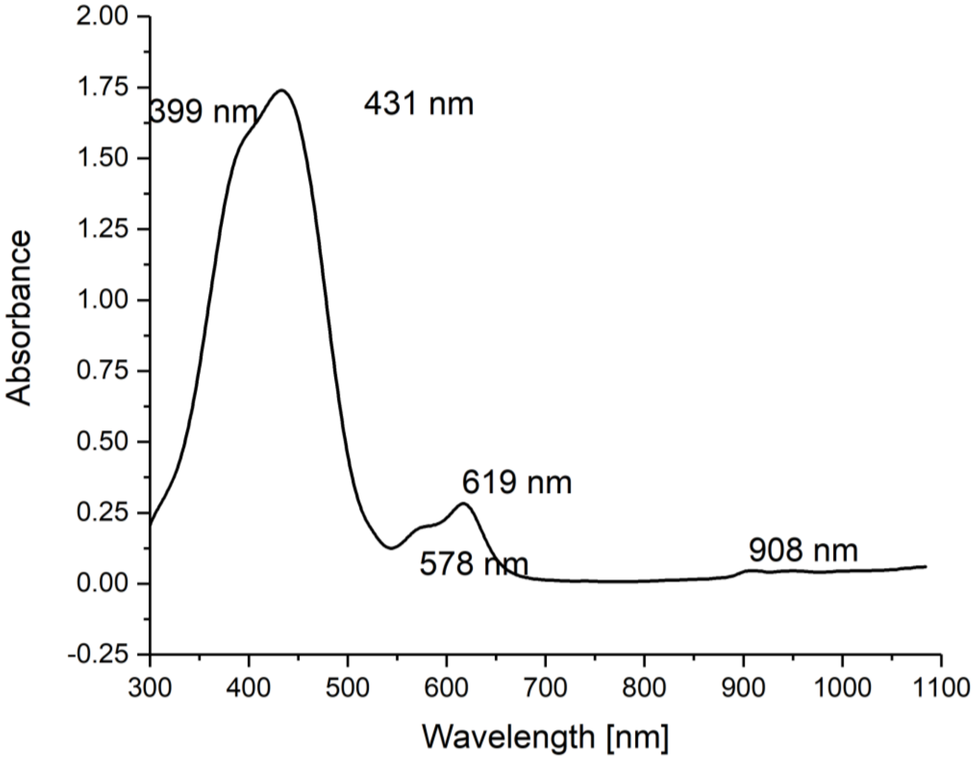

Figure S3. UV-visible spectrum of **4** (10^{-4} M) recorded in THF.

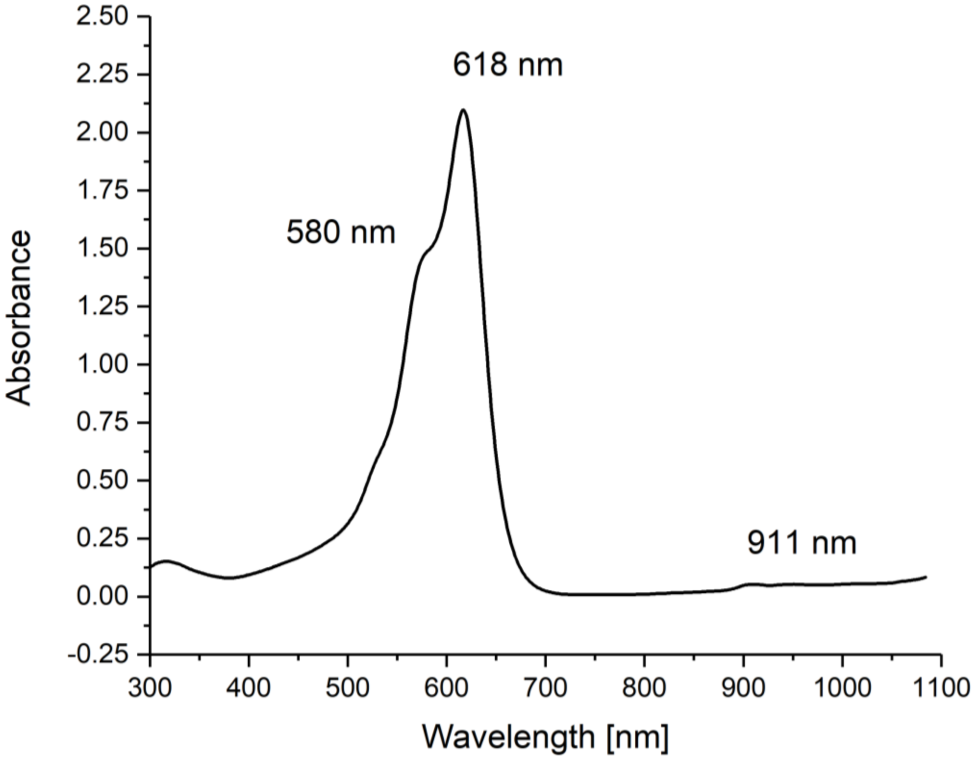

Figure S4. UV-visible spectrum of **5** (10^{-4} M) recorded in THF.

EPR spectroscopy

The continuous wave (CW) EPR experiments were performed at room temperature (298 K) in a Bruker standard ST9402 resonator and with a Bruker ELEXSY E500 spectrometer. The microwave frequency was 9.63 GHz and the modulation amplitude was 0.3 mT.

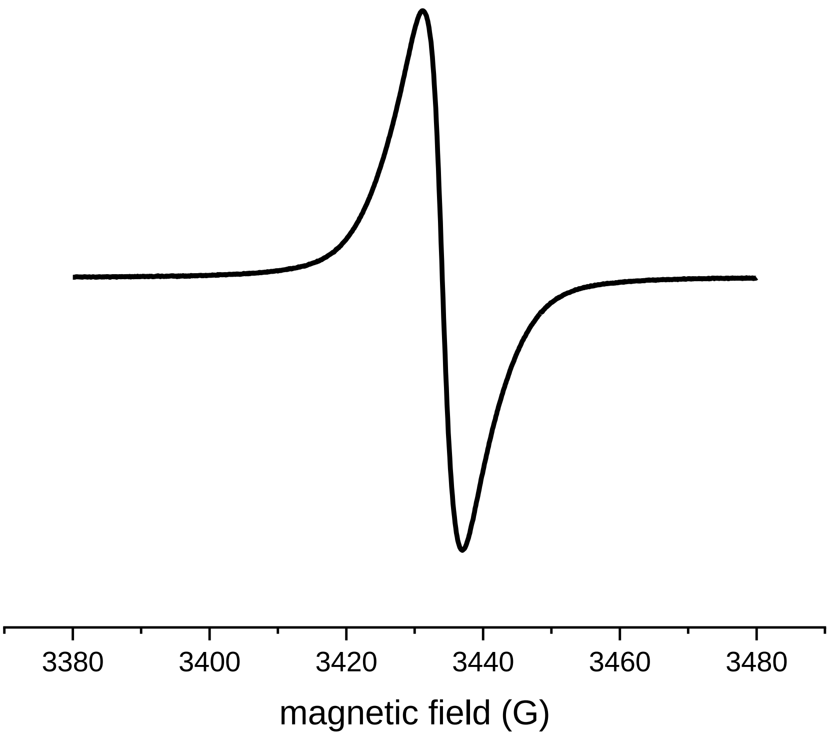

Figure S5. EPR spectrum of **5** (4 mM THF solution). Microwave freq. 9.63 GHz, power = 0.2 mW, mod. freq. 100 KHz, amp. 0.3 mT, T = 298 K.

X-Ray Diffraction Studies

Single crystals were examined on a Rigaku Supernova diffractometer using. Using Olex2[1], the structure was solved with the ShelXT[2] structure solution program using Intrinsic Phasing and refined with the ShelXL[3] refinement package using Least Squares minimization. The asymmetric unit of compound **3** contains besides the half the molecule of **3** some methanol solvent molecules, one is fully occupied, another one (O2/C35) is partly (61%) occupied. Compound **5** contains a disordered THF molecule near an inversion center, which was "squeezed" using the routine of Olex. The sum formula contains the solvent for further calculations. Additionally C10, C11 and C12 are disordered over two sites in ratio 74:26.

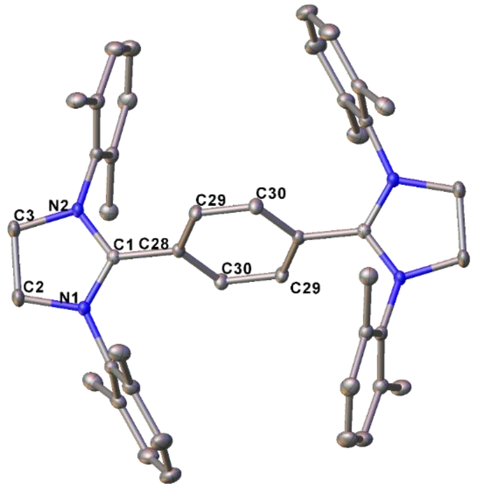

Selected bond lengths (Å) and angles (º):

C1 – N1:1.323(2)

C1 – N2: 1.329(2)

C1 – C28: 1.478(2)

C28 – C29: 1.398(2)

C28 – C30: 1.391(1)

C29 – C30: 1.399(2)

N1 – C1 – N2: 113.2(2)

Figure S6. Solid state molecular structure of **2**. H atoms and methyl of isopropyl groups as well as the iodide counter anions have been omitted for clarity

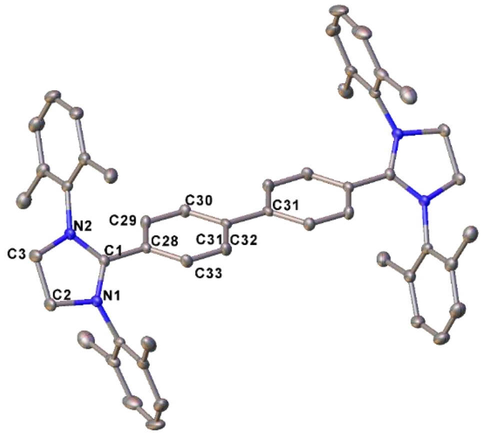

Selected bond lengths (Å) and angles (º):

C1 – N1:1.337(3)

C1 – N2: 1.325(3)

C1 – C28: 1.466(3)

C28 – C29: 1.394(3)

C28 – C33: 1.395(3)

C29 – C30: 1.384(3)

C32 – C33: 1.381(3)

C31 – C31: 1.494(4)

N1 – C1 – N2: 111.5(2)

Figure S7. Solid state molecular structure of **3**. H atoms and methyl of isopropyl groups as well as the bromide counter anions have been omitted for clarity.

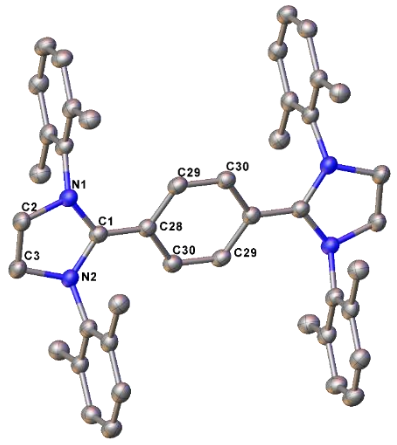

Selected bond lengths (Å) and angles (º):*

C1 – N1:1.406(3) [1.407(3)]

C1 – N2: 1.398(3) [1.405(3)]

C1 – C28: 1.376(3) [1.365(4)]

C28 – C29: 1.449(3) [1.448(3)]

C28 – C30: 1.448(3) [1.460(3)]

C29 – C30: 1.349(3) [1.347(4)]

N1 – C1 – N2: 108.5(2) [107.9(2)]

Figure S8. Solid state molecular structure of **4**. H atoms and methyl of isopropyl groups have been omitted for clarity. *The asymmetric unit contains two molecules of **4**. Only one molecule is shown above. Bond lengths and angles for the second molecule are given in square brackets.

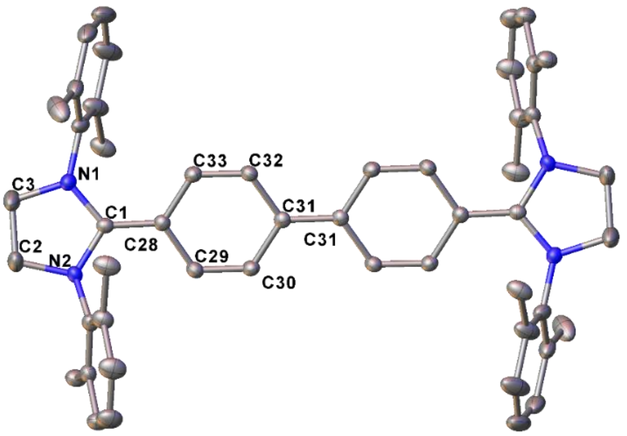

Selected bond lengths (Å) and angles (º):

C1 – N1:1.383(2)

C1 – N2: 1.397(2)

C1 – C28: 1.386(2)

C28 – C29: 1.442(2)

C28 – C33: 1.445(2)

C29 – C30: 1.360(2)

C32 – C33: 1.356(2)

C31 – C31: 1.408(2)

N1 – C1 – N2: 107.9(2)

Figure S9: Solid state molecular structure of **5**. H atoms and methyl of isopropyl groups have been omitted for clarity.

.

Table T1. Crystallographic details of compounds **2** and **3**.

	**2** · 2 MeOH	**3** · 3.2 MeOH
Empirical formula	$C_{62}H_{88}I_2N_4O_2$	$C_{69.22}H_{96.87}Br_2N_4O_{3.21}$
Formula weight	1175.16	1196.21
Radiation	Mo Kα (λ = 0.71073 Å)	MoKα (λ = 0.71073)
Temperature/K	100.0(1)	100.0(1)
Crystal system	monoclinic	monoclinic
Space group	$P2_1/n$	$P2_1/n$
a/Å	14.01776(12)	9.3343(2)
b/Å	11.68842(10)	17.7848(3)
c/Å	18.61137(19)	20.1423(4)
α/°	90	90
β/°	97.6225(9)	95.674(2)
γ/°	90	90
Volume/Å^3	3022.44(5)	3327.41(11)
Z	2	2
ρ_{calc}mg/mm^3	1.291	1.194
μ/mm^{-1}	1.083	1.265
F(000)	1220.0	1272.0
Crystal size/mm^3	0.391 × 0.308 × 0.204	0.417 × 0.113 × 0.108
2Θ range for data collection	3.428 to 60.056	3.062 to 60.16
Index ranges	-19 ≤ h ≤ 19, -16 ≤ k ≤ 16, -26 ≤ l ≤ 26	-13 ≤ h ≤ 13, -25 ≤ k ≤ 25, -28 ≤ l ≤ 28
Reflections collected	171581	192850
Independent reflections	8848 [R_{int} = 0.0508, R_{sigma} = 0.0175]	9771 [R_{int} = 0.0684, R_{sigma} = 0.0270]
Reflections with [I>=2σ (I)]	8020	7860
Completness / Θ full	1.000 / 30.0	0.998 / 30.1
Data/restraints/parameters	8848/0/329	9771/0/374
Goodness-of-fit on F^2	1.037	1.173
Final R indexes [I>=2σ (I)]	R_1 = 0.0220, wR_2 = 0.0482	R_1 = 0.0733, wR_2 = 0.1365
Final R indexes [all data]	R_1 = 0.0260, wR_2 = 0.0499	R_1 = 0.0918, wR_2 = 0.1430
Largest diff. peak/hole / e Å^{-3}	0.42/-0.42	0.68/-1.48
CCDC number	1826567	1826568

Table T2. Crystallographic details of compounds **4** and **5**.

	**4**	**5** · THF
Empirical formula	$C_{60}H_{80}N_4$	$C_{70}H_{92}N_4O$
Formula weight	857.28	1005.47
Radiation used / λ	CuKα (λ = 1.54184)	CuKα (λ = 1.54184)
Temperature/K	100.0(1)	100.0(1)
Crystal system	triclinic	triclinic
Space group	P-1	P-1
a/Å	10.4185(5)	9.3970(3)
b/Å	10.5244(5)	11.5574(5)
c/Å	25.1966(7)	15.1108(6)
α/°	97.968(3)	70.870(4)
β/°	90.283(3)	84.424(3)
γ/°	110.092(4)	76.360(3)
Volume/Å^3	2565.45(19)	1506.40(10)
Z	2	1
ρ_{calc}mg/mm^3	1.110	1.108
μ/mm^{-1}	0.479	0.488
F(000)	936.0	548.0
Crystal size/mm^3	0.21 × 0.133 × 0.037	0.36 × 0.167 × 0.026
2Θ range for data collection	7.096 to 144.256	6.192 to 144.254
Index ranges	-12 ≤ h ≤ 12, -12 ≤ k ≤ 12, -31 ≤ l ≤ 31	-11 ≤ h ≤ 11, -13 ≤ k ≤ 14, -18 ≤ l ≤ 18
Reflections collected	37630	25880
Independent reflections	9804 [R_{int} = 0.0436, R_{sigma} = 0.0344]	5928 [R_{int} = 0.0269, R_{sigma} = 0.0217]
Reflections with [I>=2σ (I)]	7686	5051
Completness / Θ full/°	0.971 / 71.2	0.998 / 71.2
Data/restraints/parameters	9804/0/593	5928/0/337
Goodness-of-fit on F^2	1.068	1.038
Final R indexes [I>=2σ (I)]	R_1 = 0.0716, wR_2 = 0.2049	R_1 = 0.0483, wR_2 = 0.1243
Final R indexes [all data]	R_1 = 0.0881, wR_2 = 0.2208	R_1 = 0.0563, wR_2 = 0.1314
Largest diff. peak/hole / e Å^{-3}	0.36/-0.32	0.47/-0.42
CCDC number	1826569	1826570

Computational Studies

Geometry optimizations were performed using the Gaussian 09 optimizer[4] together with TurboMole V6.5.[5] All geometry optimizations were computed using the functional B3LYP[6] and BH&HLYP[7] in combination with the def2-SVP basis set.[8] The stationary points were located with the Berny algorithm[9] using redundant internal coordinates. Analytical Hessians were computed to determine the nature of stationary points. The improvements in the electronic energies were carried out by computing single points on the B3LYP/def2-SVP geometries at the B3LYP/def2-TZVPP, BH&HLYP/def2-TZVPP, PBE0/def2-TZVPP[10] and M06-2X/def2-TZVPP[11] levels of theory.

Time-dependent density functional theory (TDDFT) was employed to calculate excitation energies as implemented in ORCA 4.0.1.2.[12] We used the functional B3LYP in combination the def2-SVP basis sets. The solvent THF was described in this case by the conductor-like polarizable continuum model, CPCM.[13]

The diradical character (y_i) is defined by the weight of the doubly excited configuration in the multi-configurational (MC)-SCF theory and is formally expressed in the spin-projected UFH (PUHF) theory[14] as:

$$y_i = 1 - \frac{2T_i}{1 + T_i^2} \qquad (1)$$

Where T_i is the orbital overlap between the orbital pair and it ca be represented by the occupations numbers (n_i) of the UHF natural orbitals (UNOs):

$$T_i = \frac{n_{HOMO-i} - n_{LUMO+i}}{2} \qquad (2)$$

The diradical character y_i obtained from the UNO occupations number have a value between 0 and 1. In purely closed-shell system n_{HOMO-i} and n_{LUMO+i} = 0, then y = 0. When the occupations of the two orbitals are equal the system is a pure diradical and y = 1. The diradical characters have been computed from the occupation number of the lowest unoccupied natural orbital (LUNO) at the UHF/6-31G(d,p) level of theory.

The CASSCF(2,2)+NEVPT2/def2-SVP calculations were performed on the model systems **4^{Me}** and **5^{Me}** using the ORCA 4.0.1.2 software.[12] The singlet diradical index *d* proposed by Neese and co-workers[14-15] was calculated, for an *ab initio* CI calculation with the canonical MOs, by the following equation:

$$d = 200\sqrt{\frac{c_0^2 c_d^2}{c_0^2 + c_d^2}}$$

Where c_0^2 is the weight of the closed- shell configuration in the CI wave function and c_d^2 is weight of the double excitation computed.

Table T3. Compound **4** electronic energies (*E* in Hartrees) and relative electronic energies (ΔE_r in kcal/mol) of the different electronic states: Singlet closed-shell (CS) and triplet (T) states.

Methods	*E*		ΔE_{S-T}
	CS	T	
B3LYP/def2-SVP	-2551.72784856	-2551.68277394	28.3
BH&HLYP/def2-SVP	-2550.10914948	-2550.06892412	25.2
B3LYP/def2-TZVPP	-2554.49021815	-2554.44376760	29.1
BH&HLYP /def2-TZVPP	-2552.82261847	-2552.77823921	27.8
PBE0/def2-TZVPP	-2551.39027466	-2551.34452486	28.7
M06-2X/def2-TZVPP	-2553.38145089	-2553.33052231	32.0

Table T4. Compound **5** electronic energies (*E* in Hartrees) and relative electronic energies (ΔE_r in kcal/mol) of the different electronic states: Singlet closed-shell (CS) and Triplet (T) states.

Methods	*E*		ΔE_{S-T}
	CS	T	
B3LYP/def2-SVP	-2782.60842094	-2782.59283786	9.8
BH&HLYP/def2-SVP	-2780.84082482	-2780.83459499	3.9
B3LYP/def2-TZVPP	-2785.61835868	-2785.60135209	10.7
BH&HLYP/def2-TZVPP	-2783.79914917	-2783.79151100	4.8
PBE0/def2-TZVPP	-2782.23907035	-2782.22395290	9.5
M06-2X/def2-TZVPP	-2784.40606753	-2784.39037670	9.8

Table T5. Compound $\mathbf{4^{Me}}$ electronic energies (*E* in Hartrees) and relative electronic energies (ΔE_r in kcal/mol) of the different electronic states: Singlet closed-shell (CS) and triplet (T) states.

Methods	*E*		ΔE_{S-T}
	CS	T	
B3LYP/def2-SVP	-842.47358585	-842.43377335	25.0
BH&HLYP/def2-SVP	-841.93987354	-841.90791990	20.1
B3LYP/def2-TZVPP	-843.39412122	-843.35521126	24.4
BH&HLYP /def2-TZVPP	-842.84626353	-842.81443241	20.0
PBE0/def2-TZVPP	-842.38674667	-842.34826853	24.1
M06-2X/def2-TZVPP	-843.02230632	-842.97880260	27.3
CASSCF(2,2)/ /def2-SVP	-836.94670369	-836.86884504	48.9
CASSCF(2,2) + NEVPT2/def2-SVP	-839.7954621	-839.7321582	39.7

Table T6. Compound **5**^{Me} electronic energies (E in Hartrees) and relative electronic energies (ΔE_r in kcal/mol) of the different electronic states: Singlet closed-shell (CS) and Triplet (T) states.

Methods	*E*		ΔE_{S-T}
	CS	T	
B3LYP/def2-SVP	-1073.35448377	-1073.34348667	6.9
BH&HLYP/def2-SVP	-1072.67063564	-1072.67121070	-0.4
B3LYP/def2-TZVPP	-1074.52219951	-1074.51032097	7.5
BHandHLYP/def2-TZVPP	-1073.82192184	-1073.82160646	0.2
PBE0/def2-TZVPP	-1073.23618872	-1073.22652184	6.1
M06-2X/def2-TZVPP	-1074.04745975	-1074.03874829	5.5
CASSCF(2,2) /def2-SVP	-1066.29796717	-1066.25916564	24.3
CASSCF(2,2) + NEVPT2/def2-SVP	-1069.94188434	-1069.91002208	20.0

Table T7. CASSCF(2,2)+NEVPT2/def2-SVP Energies (in Hartree), HOMO- LUMO occupations, CI vectors and diradicaloid character of **4**^{Me} and **5**^{Me}.

CI vector	**4**^{Me}	**5**^{Me}
20	0.96461	0.96068
02	0.03539	0.03932
Occ(HOMO)	1.9292	1.9214
Occ(LUMO)	0.0708	0.0786
CASSCF(2,2)	-836.94670369	-1066.29796717
CASSCF(2,2)+NEVPT2	-839.7954621	-1069.94188434
d	7.0 %	7.8 %

Table T8. TD-PCM(THF)[B3LYP/def2-SVP] results for compound **4** as a singlet closed-shell species and triplet in the THF.[a] Wavelength (λ), oscillator strength (f) and main assignment.[b]

	**Singlet Closed-Shell**			**Triplet**		
	**λ(nm)**	**f**	**Assignment**	**λ(nm)**	**f**	**Assignment**
**1**	539.7	0.0662	HOMO → LUMO (c= 0.54)	841.5	0.0265	SOMO(α) → LUMO+7 (c= 0.96)
**2**	350.7	1.3860	HOMO → LUMO+8 (c= 0.79)	502.0	0.1267	SOMO(α) → LUMO + 10 (c= -0.91)
**3**	256.4	0.0925	HOMO-1 -> LUMO (c= -0.82)	328.9	0.4354	HOMO-2(β) → LUMO (c= 0.86)

[a] All the geometry was optimized at the B3LYP/def2-SVP. [b] H means HOMO and L means LUMO.

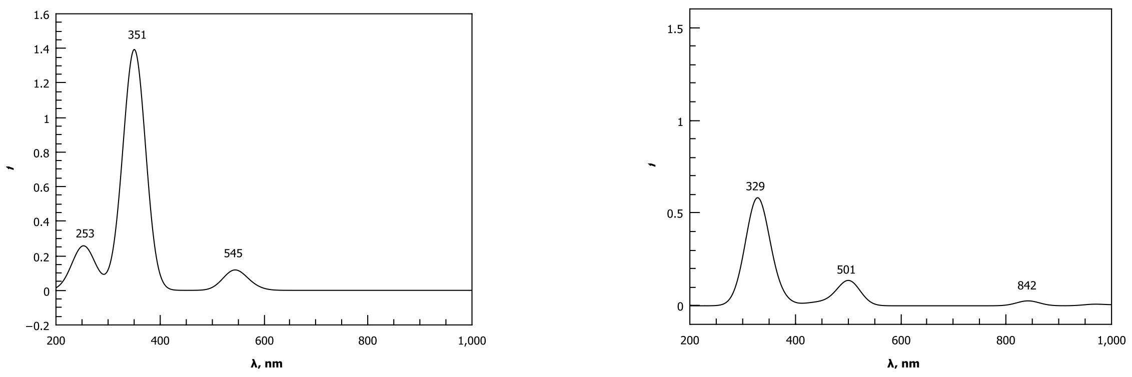

(A) (B)

Figure S10. TD-DFT spectra simulation of compound **4** in singlet state in the THF solvent at the B3LYP/def2-SVP for (A) singlet close shell and (B) triplet species. The middle bandwidth was arbitrary chosen as 50 nm.

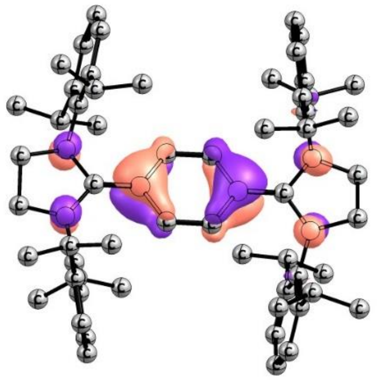

HOMO-2

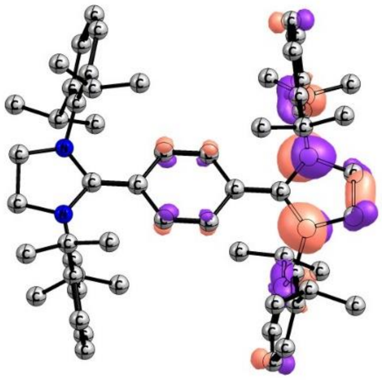

HOMO-1

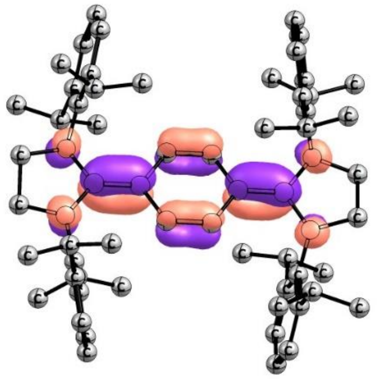

HOMO

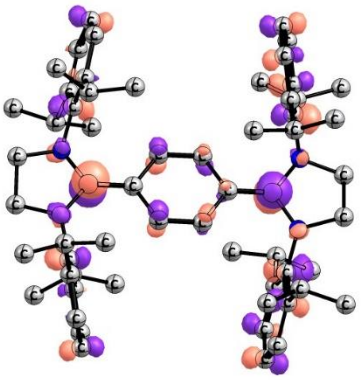

LUMO

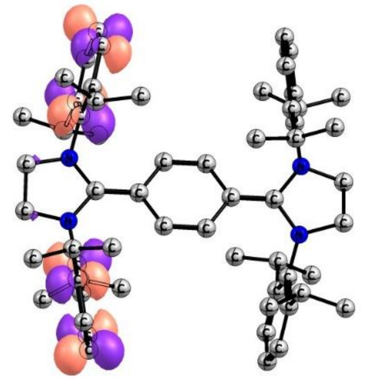

LUMO+1

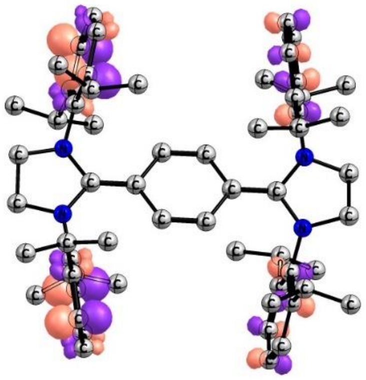

LUMO+2

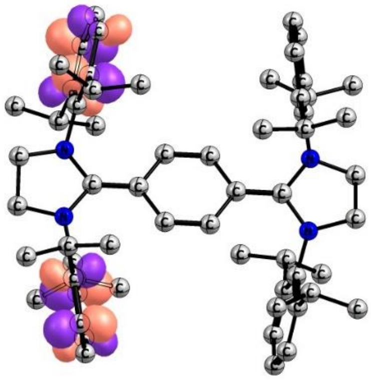

LUMO+3

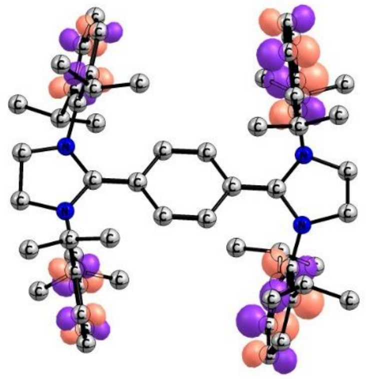

LUMO+4

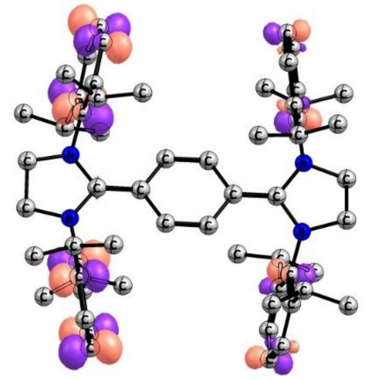

LUMO+5

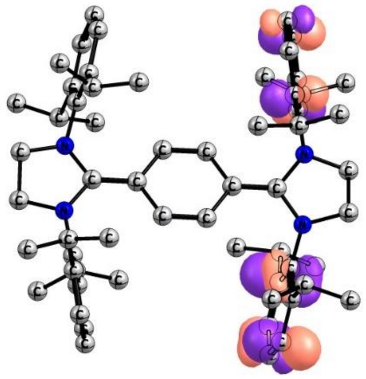

LUMO+6

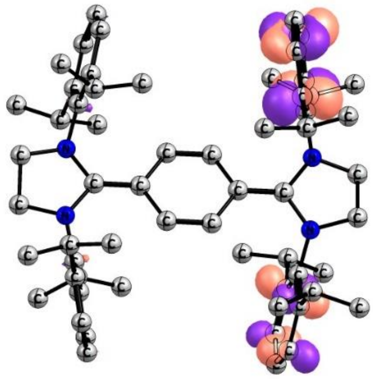

LUMO+7

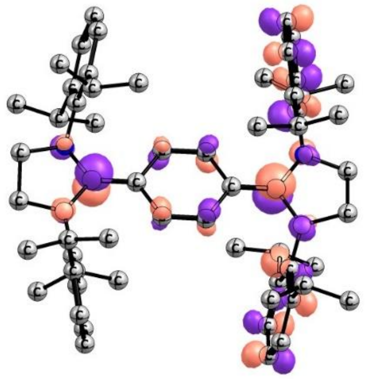

LUMO+8

Figure S11. Frontier KS molecular orbitals (isocontour 0.05 au) of compounds **4** as singlet closed-shell (B3LYP/def2-SVP). Hydrogen atoms are omitted for clarity.

Table T9. TD-PCM(THF)[B3LYP/def2-SVP] results for compound **5** as a singlet closed-shell species and triplet in the THF.[a] Wavelength (λ), oscillator strength (f) and main assignment.[b]

	**Singlet Close Shell**			**Triplet**		
	**λ(nm)**	**f**	**Assignment**	**λ(nm)**	**f**	**Assignment**
**1**	589.0	0.3480	HOMO -> LUMO+10 (c= -0.67)	771.4	0.0623	SOMO(α) -> LUMO+2 (c= 0.94)
**2**	441.4	3.2957	HOMO -> LUMO (c= 0.84)	709.1	0.0275	SOMO(α) → LUMO+6 (c= 0.94)
**3**	258.5	0.1554	HOMO-3 -> LUMO (c= -0.88)	501.9	0.2193	SOMO(α) → LUMO+8 (c= 0.88)
**4**				369.4	1.0640	HOMO(β) → LUMO (c= -0.83)

[a] All the geometry was optimized at the B3LYP/def2-SVP. [b] H means HOMO and L means LUMO.

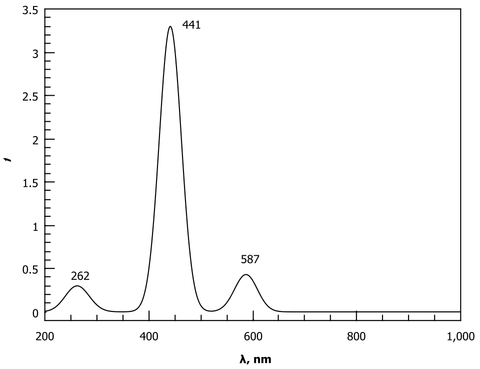

(A)

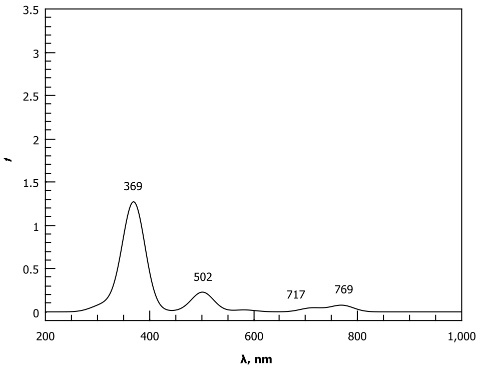

(B)

Figure S12. TD-DFT spectra simulation of compound **5** in singlet state in the THF solvent at the B3LYP/def2-SVP for (A) singlet close shell and (B) triplet species. The middle band width was arbitrary chosen as 50 nm.

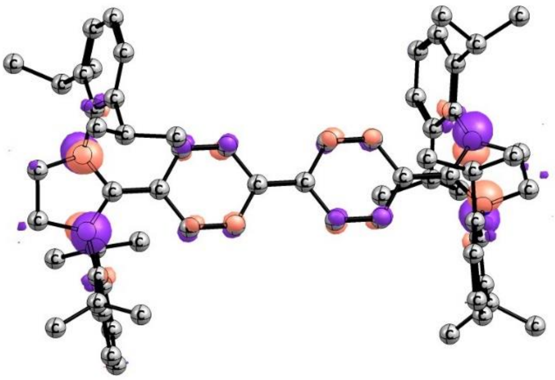

HOMO-2

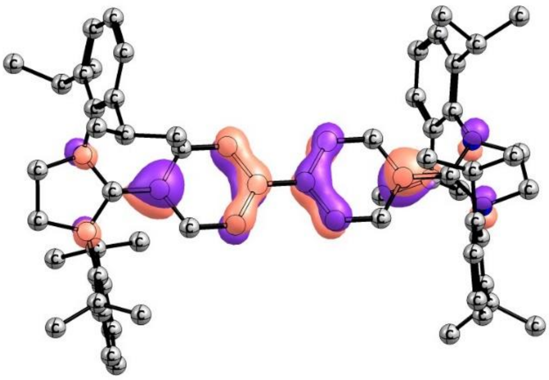

HOMO-1

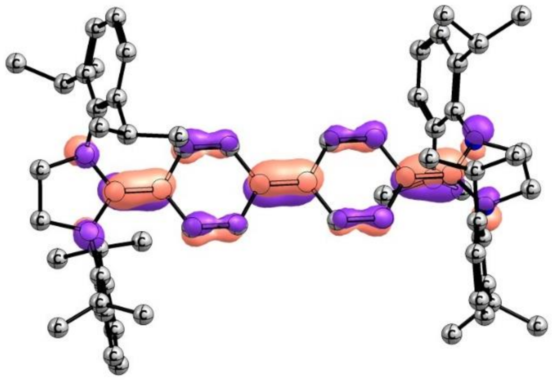

HOMO

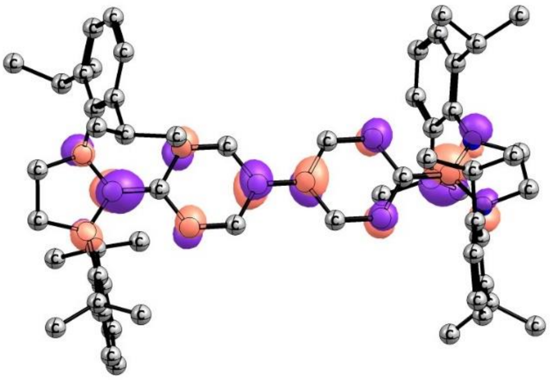

LUMO

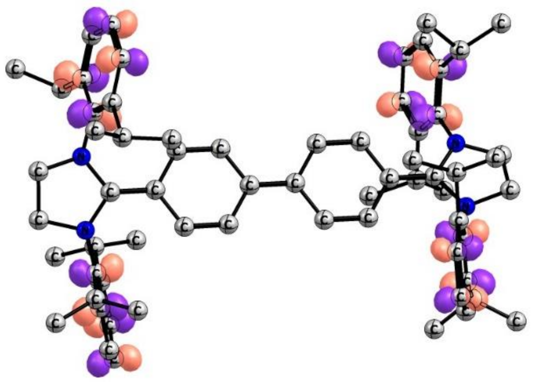

LUMO+1

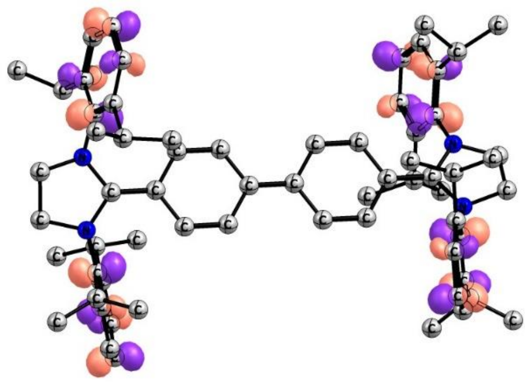

LUMO+2

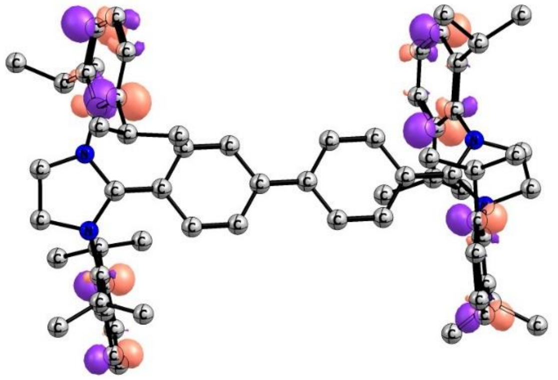

LUMO+3

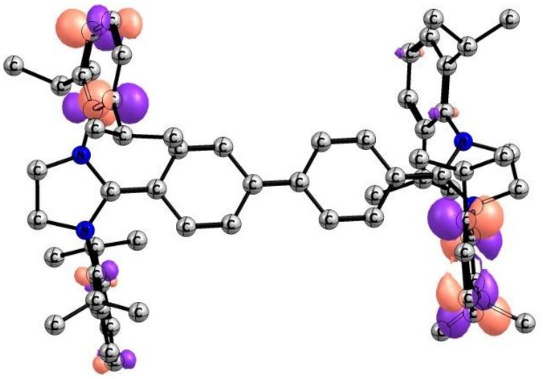

LUMO+4

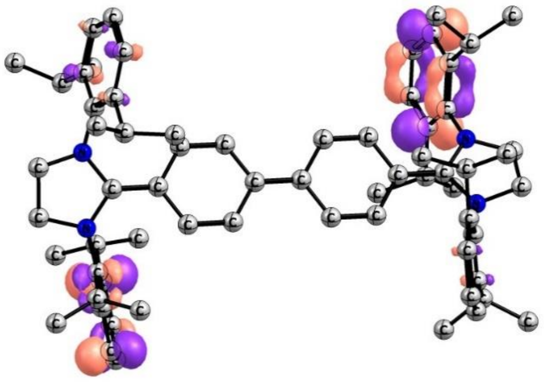

LUMO+5

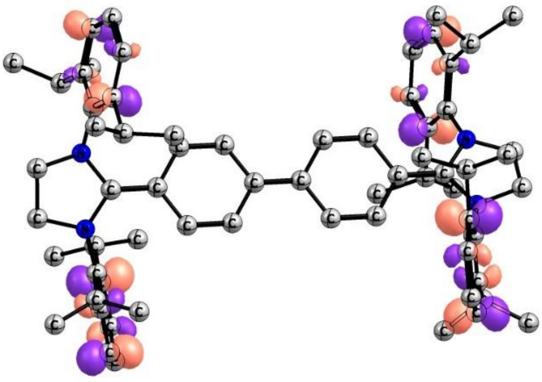

LUMO+6

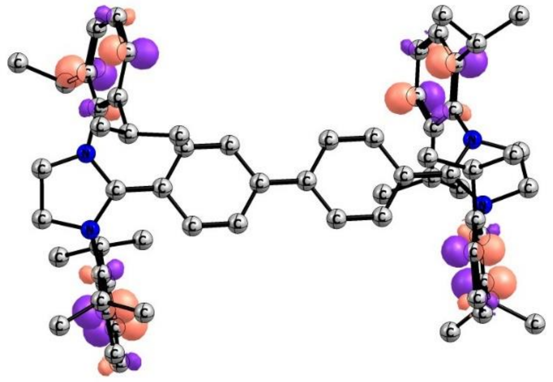

LUMO+7

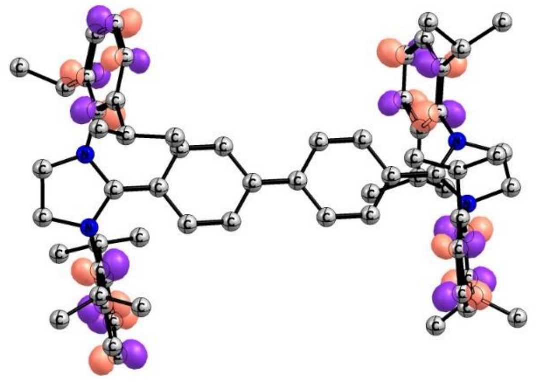

LUMO+8

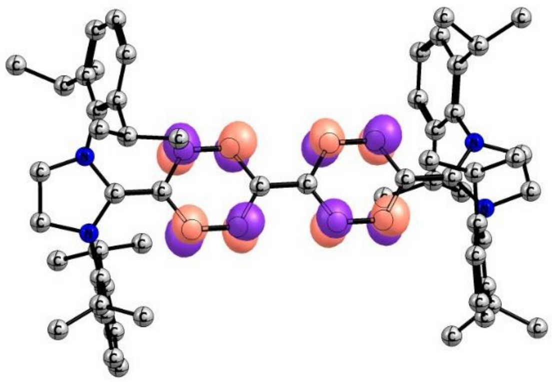

LUMO+9

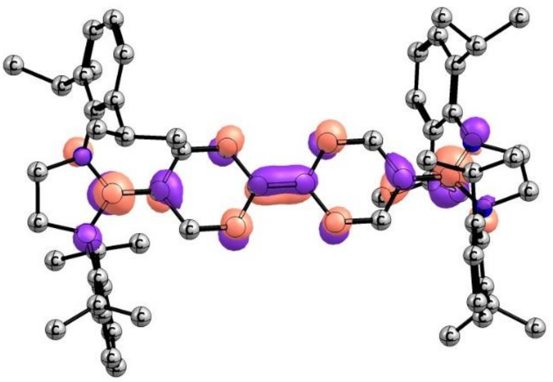

LUMO+10

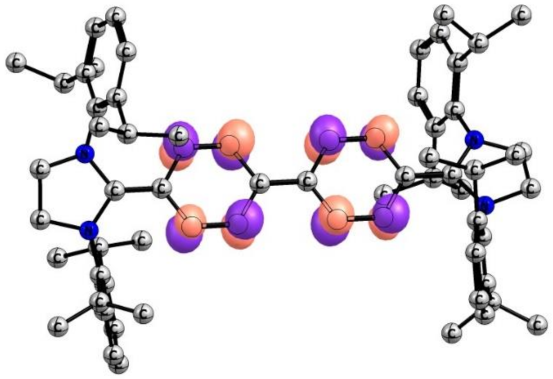

LUMO+11

Figure S13. Frontier KS molecular orbitals (isocontour 0.05 au) of compounds **5** as singlet closed-shell (B3LYP/def2-SVP). Hydrogen atoms are omitted for clarity.

xyz coordinate (in Å) and energies in (Hartree) at the B3LYP/def2-SVP and BH&HLYP levels of theory.

2-B3LYP

7	-3.636469	0.857737	-0.704548
6	-2.892897	0.000015	0.000004
7	-3.636413	-0.857729	0.704595
6	-5.075313	0.641619	-0.422364
1	-5.465687	1.515229	0.121876
6	-5.075276	-0.641651	0.422485
6	-1.407753	0.000044	-0.000006
6	-0.697126	-0.008001	1.210915
6	-0.697129	0.008102	-1.210927
6	0.697123	0.008129	1.210913
6	0.697121	-0.008029	-1.210929
6	1.407748	0.000042	-0.000008
1	-1.226733	-0.011008	2.162047
1	-1.226739	0.011079	-2.162057
1	1.226734	0.011131	2.162043
1	1.226727	-0.011055	-2.162060
1	-5.637660	-0.546287	1.360612
6	-3.205464	2.036821	-1.427010
6	-2.892146	3.209549	-0.697815
6	-3.212631	1.999129	-2.843003
6	-2.530496	4.346936	-1.434797
6	-2.846810	3.170015	-3.521145
6	-2.503114	4.329502	-2.827846
1	-2.284126	5.270892	-0.907704
1	-2.841723	3.179834	-4.612809
1	-2.228633	5.232092	-3.378860
6	-3.205336	-2.036813	1.427011
6	-3.212437	-1.999127	2.843002
6	-2.892047	-3.209536	0.697796
6	-2.846569	-3.170011	3.521123
6	-2.530327	-4.346915	1.434756
6	-2.502876	-4.329486	2.827803
1	-2.841436	-3.179835	4.612787
1	-2.283963	-5.270864	0.907646
1	-2.228354	-5.232076	3.378798
6	-3.645737	-0.770403	3.642134
1	-3.729858	0.077324	2.941541
6	-2.624951	-0.365988	4.721195
1	-2.540809	-1.129123	5.509845
1	-1.615605	-0.210245	4.306852
1	-2.937411	0.570173	5.209078
6	-5.034810	-0.986836	4.277296
1	-5.796576	-1.260431	3.530548
1	-5.006128	-1.797501	5.022101
1	-5.373029	-0.073483	4.791201
6	-2.989196	-3.308777	-0.823912
1	-3.202255	-2.304940	-1.225683
6	-4.160620	-4.219562	-1.243667
1	-4.265042	-4.235008	-2.339950
1	-3.999192	-5.256685	-0.910723
1	-5.118093	-3.884402	-0.814645
6	-1.672932	-3.776814	-1.468881
1	-0.826146	-3.131403	-1.189443
1	-1.420640	-4.806545	-1.171788
1	-1.761675	-3.769244	-2.566627
6	-3.645925	0.770394	-3.642121
1	-3.729962	-0.077349	-2.941537
6	-2.625184	0.366050	-4.721252
1	-1.615807	0.210341	-4.306975
1	-2.937632	-0.570110	-5.209146
1	-2.541125	1.129215	-5.509884
6	-5.035042	0.986773	-4.277198
1	-5.373245	0.073412	-4.791100
1	-5.796780	1.260314	-3.530401
1	-5.006448	1.797452	-5.021991
6	-2.989227	3.308783	0.823901
1	-3.202350	2.304957	1.225665
6	-4.160559	4.219659	1.243713

1	-5.118072	3.884596	0.814705
1	-4.264952	4.235078	2.339999
1	-3.999052	5.256780	0.910800
6	-1.672898	3.776694	1.468829
1	-1.761589	3.769081	2.566579
1	-0.826173	3.131231	1.189324
1	-1.420545	4.806418	1.171767
6	2.892892	0.000017	-0.000012
7	3.636453	0.857759	0.704526
6	5.075303	0.641617	0.422392
6	5.075277	-0.641665	-0.422441
7	3.636418	-0.857752	-0.704564
1	5.465657	-1.515278	0.121789
1	5.637698	0.546246	1.360511
6	3.205353	-2.036856	-1.426956
6	3.212559	-1.999242	-2.842950
6	2.891988	-3.209539	-0.697706
6	2.846713	-3.170152	-3.521037
6	2.530289	-4.346943	-1.434635
6	2.502939	-4.329583	-2.827685
1	2.841665	-3.180034	-4.612701
1	2.283866	-5.270861	-0.907497
1	2.228437	-5.232194	-3.378655
6	3.205433	2.036866	1.426942
6	2.892163	3.209581	0.697706
6	3.212527	1.999203	2.842936
6	2.530487	4.346987	1.434646
6	2.846679	3.170106	3.521034
6	2.503032	4.329582	2.827694
1	2.284151	5.270934	0.907521
1	2.841532	3.179948	4.612699
1	2.228529	5.232186	3.378674
6	3.645966	-0.770578	-3.642117
1	3.730010	0.077199	-2.941577
6	5.035114	-0.987071	-4.277095
1	5.796783	-1.260627	-3.530234
1	5.006513	-1.797785	-5.021850
1	5.373409	-0.073757	-4.791019
6	2.625317	-0.366234	-4.721335
1	2.541266	-1.129427	-5.509940
1	1.615921	-0.210451	-4.307131
1	2.937845	0.569889	-5.209249
6	2.989042	-3.308713	0.824013
1	3.202098	-2.304864	1.225753
6	1.672731	-3.776694	1.468926
1	0.825975	-3.131278	1.189408
1	1.420435	-4.806434	1.171868
1	1.761409	-3.769072	2.566677
6	4.160423	-4.219506	1.243877
1	4.264785	-4.234903	2.340166
1	3.998987	-5.256640	0.910973
1	5.117926	-3.884390	0.814891
6	2.989322	3.308782	-0.824006
1	3.202446	2.304943	-1.225739
6	1.673035	3.776706	-1.469010
1	1.761782	3.769071	-2.566756
1	0.826284	3.131263	-1.189538
1	1.420685	4.806439	-1.171981
6	4.160694	4.219626	-1.243777
1	5.118178	3.884554	-0.814711
1	4.265145	4.235019	-2.340058
1	3.999189	5.256757	-0.910895
6	3.645762	0.770479	3.642102
1	3.729852	-0.077272	2.941535
6	5.034832	0.986870	4.277282
1	5.373002	0.073514	4.791215
1	5.796624	1.260413	3.530541
1	5.006175	1.797553	5.022068
6	2.624942	0.366145	4.721161
1	1.615597	0.210426	4.306809
1	2.937356	-0.570009	5.209089
1	2.540820	1.129317	5.509779
1	5.637668	-0.546309	-1.360565
1	5.465709	1.515215	-0.121843
1	-5.637743	0.546245	-1.360462
1	-5.465655	-1.515273	-0.121732

2-BH&HLYP

7	-3.605786	-0.850974	0.695128
6	-2.871604	-0.000009	0.000208
7	-3.605894	0.850976	-0.694575
6	-5.032526	-0.640705	0.415369
1	-5.416886	-1.503391	-0.133458
6	-5.032586	0.640752	-0.414535
6	-1.392916	-0.000019	0.000096
6	-0.692378	0.008961	-1.203485
6	-0.692205	-0.009009	1.203577
6	0.692205	-0.009034	-1.203584
6	0.692379	0.008982	1.203478
6	1.392916	-0.000022	-0.000103
1	-1.220372	0.015289	-2.146198
1	-1.220064	-0.015336	2.146366
1	1.220064	-0.015380	-2.146372
1	1.220372	0.015325	2.146190
1	-5.593998	0.552588	-1.343791
6	-3.170838	-2.017828	1.415786
6	-2.844291	-3.177713	0.695103
6	-3.180016	-1.978634	2.820154
6	-2.474902	-4.303308	1.426893
6	-2.806757	-3.135344	3.497127
6	-2.452130	-4.284018	2.810282
1	-2.218929	-5.217813	0.905141
1	-2.804588	-3.143796	4.580468
1	-2.172190	-5.176352	3.358846
6	-3.171060	2.017820	-1.415318
6	-3.180693	1.978687	-2.819685
6	-2.844230	3.177660	-0.694692
6	-2.807600	3.135410	-3.496729
6	-2.475010	4.303265	-1.426551
6	-2.452684	4.284033	-2.809949
1	-2.805793	3.143911	-4.580071
1	-2.218822	5.217735	-0.904843
1	-2.172877	5.176377	-3.358564
6	-3.623778	0.758878	-3.610346
1	-3.713260	-0.080454	-2.913932
6	-2.615544	0.348583	-4.683872
1	-2.529147	1.105345	-5.466751
1	-1.614177	0.186398	-4.274928
1	-2.935084	-0.578493	-5.165915
6	-5.001361	0.986941	-4.239445
1	-5.752494	1.268621	-3.497288
1	-4.963830	1.789443	-4.980513
1	-5.346998	0.082602	-4.746714
6	-2.928673	3.275094	0.819089
1	-3.155354	2.282558	1.218166
6	-4.071961	4.199809	1.244484
1	-4.167533	4.215379	2.333283
1	-3.894323	5.226121	0.913559
1	-5.030156	3.883781	0.823989
6	-1.607893	3.716881	1.447790
1	-0.781144	3.062921	1.160688
1	-1.344483	4.734992	1.150884
1	-1.686119	3.708701	2.538096
6	-3.622737	-0.758750	3.610903
1	-3.712500	0.080525	2.914457
6	-2.614027	-0.348381	4.683952
1	-1.612837	-0.186254	4.274552
1	-2.933337	0.578744	5.166052
1	-2.527302	-1.105074	5.466861
6	-5.000051	-0.986732	4.240623
1	-5.345452	-0.082341	4.747960
1	-5.751513	-1.268469	3.498822
1	-4.962210	-1.789167	4.981748
6	-2.929217	-3.275202	-0.818648
1	-3.155989	-2.282672	-1.217690
6	-4.072674	-4.199891	-1.243646
1	-5.030723	-3.883814	-0.822857
1	-4.168593	-4.215497	-2.332413
1	-3.894968	-5.226198	-0.912741
6	-1.608653	-3.717058	-1.447752
1	-1.687226	-3.708918	-2.538034
1	-0.781790	-3.063115	-1.160940
1	-1.345183	-4.735167	-1.150891
6	2.871605	-0.000017	-0.000216

7	3.605783	-0.851032	-0.695078
6	5.032525	-0.640750	-0.415333
6	5.032590	0.640753	0.414501
7	3.605897	0.851008	0.694515
1	5.416834	1.503417	-0.134440
1	5.593748	-0.552617	-1.344705
6	3.171065	2.017893	1.415193
6	3.180723	1.978853	2.819562
6	2.844207	3.177680	0.694495
6	2.807627	3.135617	3.496535
6	2.474983	4.303328	1.426285
6	2.452682	4.284189	2.809684
1	2.805840	3.144192	4.579876
1	2.218772	5.217759	0.904520
1	2.172873	5.176566	3.358245
6	3.170827	-2.017926	-1.415665
6	2.844209	-3.177739	-0.694902
6	3.180071	-1.978847	-2.820037
6	2.474810	-4.303382	-1.426614
6	2.806809	-3.135603	-3.496931
6	2.452107	-4.284207	-2.810007
1	2.218778	-5.217833	-0.904798
1	2.804695	-3.144147	-4.580271
1	2.172163	-5.176577	-3.358509
6	3.623849	0.759107	3.610298
1	3.713309	-0.080277	2.913945
6	5.001457	0.987230	4.239322
1	5.752557	1.268858	3.497111
1	4.963951	1.789789	4.980329
1	5.347122	0.082933	4.746647
6	2.615666	0.348887	4.683900
1	2.529303	1.105704	5.466728
1	1.614280	0.186670	4.275015
1	2.935232	-0.578153	5.165995
6	2.928615	3.275007	-0.819295
1	3.155313	2.282447	-1.218303
6	1.607812	3.716713	-1.448001
1	0.781085	3.062752	-1.160834
1	1.344381	4.734839	-1.151167
1	1.686015	3.708453	-2.538308
6	4.071871	4.199722	-1.244780
1	4.167420	4.215215	-2.333582
1	3.894213	5.226053	-0.913925
1	5.030083	3.883750	-0.824281
6	2.929057	-3.275099	0.818861
1	3.155851	-2.282543	1.217825
6	1.608446	-3.716842	1.447944
1	1.686969	-3.708608	2.538229
1	0.781625	-3.062887	1.161036
1	1.344944	-4.734965	1.151163
6	4.072456	-4.199799	1.243994
1	5.030539	-3.883799	0.823224
1	4.168322	-4.215315	2.332768
1	3.894723	-5.226127	0.913169
6	3.622884	-0.759050	-3.610869
1	3.712608	0.080291	-2.914498
6	5.000245	-0.987124	-4.240453
1	5.345706	-0.082792	-4.747853
1	5.751641	-1.268802	-3.498562
1	4.962448	-1.789633	-4.981501
6	2.614274	-0.348765	-4.684044
1	1.613051	-0.186578	-4.274747
1	2.933644	0.578307	-5.166207
1	2.527600	-1.105532	-5.466886
1	5.593989	0.552630	1.343769
1	5.416887	-1.503406	0.133537
1	-5.593754	-0.552523	1.344733
1	-5.416811	1.503450	0.134366

3-B3LYP

6	5.058263	0.000024	-0.000013
7	5.809989	-1.049010	0.360678
6	7.243890	0.682045	-0.350381
1	7.833828	1.442992	0.176545
6	7.243906	-0.681962	0.350328
6	3.579959	0.000015	-0.000009
6	2.862662	-1.113119	-0.473773

6	2.862655	1.113146	0.473752
6	1.470075	-1.105518	-0.481669
6	1.470068	1.105536	0.481648
6	0.743118	0.000006	-0.000010
1	3.388667	-1.989469	-0.847602
1	3.388654	1.989501	0.847578
1	0.942452	-1.989386	-0.844830
1	0.942439	1.989402	0.844806
1	7.604079	-0.630776	1.390375
6	5.392600	2.289194	-0.979291
6	5.007329	2.288363	-2.341310
6	5.475442	3.478462	-0.213992
6	4.659515	3.521338	-2.912715
6	5.122940	4.679364	-0.844478
6	4.713089	4.702742	-2.176615
1	4.355055	3.558911	-3.960721
1	5.176003	5.615625	-0.285202
1	4.445662	5.651365	-2.648015
6	5.392652	-2.289142	0.979266
6	5.475506	-3.478402	0.213958
6	5.007412	-2.288330	2.341296
6	5.123040	-4.679317	0.844441
6	4.659636	-3.521318	2.912697
6	4.713217	-4.702714	2.176586
1	5.176112	-5.615572	0.285155
1	4.355203	-3.558908	3.960710
1	4.445821	-5.651347	2.647983
6	5.957773	-3.504025	-1.236299
1	6.052389	-2.460828	-1.579921
6	4.960500	-4.197802	-2.182065
1	4.849881	-5.267248	-1.945544
1	3.957948	-3.743190	-2.136271
1	5.312498	-4.129667	-3.223161
6	7.348887	-4.159808	-1.348264
1	8.091634	-3.674530	-0.695558
1	7.310435	-5.222251	-1.060661
1	7.719660	-4.108567	-2.384036
6	4.994560	-1.035846	3.215555
1	5.238910	-0.167068	2.584002
6	6.076104	-1.111250	4.311315
1	6.099643	-0.177951	4.895801
1	5.879850	-1.937014	5.012901
1	7.079556	-1.272987	3.886873
6	3.607371	-0.768039	3.826559
1	2.829206	-0.697050	3.051134
1	3.313341	-1.565830	4.526371
1	3.615128	0.176325	4.393526
6	5.957729	3.504109	1.236259
1	6.052393	2.460917	1.579883
6	4.960437	4.197846	2.182034
1	3.957904	3.743190	2.136255
1	5.312451	4.129732	3.223126
1	4.849768	5.267287	1.945510
6	7.348815	4.159953	1.348205
1	7.719604	4.108729	2.383972
1	8.091575	3.674709	0.695488
1	7.310312	5.222395	1.060603
6	4.994479	1.035868	-3.215552
1	5.238871	0.167105	-2.583994
6	6.075985	1.111281	-4.311349
1	7.079446	1.273050	-3.886942
1	6.099528	0.177974	-4.895820
1	5.879687	1.937029	-5.012941
6	3.607275	0.768017	-3.826504
1	3.615037	-0.176353	-4.393461
1	2.829139	0.697015	-3.051050
1	3.313199	1.565793	-4.526313
6	-0.743905	0.000001	-0.000009
6	-1.470994	-0.619359	-1.034695
6	-1.470999	0.619355	1.034678
6	-2.863549	-0.627362	-1.034433
6	-2.863554	0.627343	1.034418
6	-3.580697	-0.000009	-0.000010
1	-0.943539	-1.084166	-1.869626
1	-0.943547	1.084167	1.869607
1	-3.389674	-1.105830	-1.858328
1	-3.389682	1.105802	1.858315

6	-5.058849	-0.000026	-0.000003
7	-5.810312	1.062438	0.318955
6	-7.244296	0.758240	0.113708
6	-7.244274	-0.758368	-0.113640
7	-5.810286	-1.062515	-0.318936
1	-7.604622	-1.323818	0.760670
1	-7.833921	1.068487	0.985983
6	-5.391930	-2.430599	-0.538058
6	-5.010574	-3.223616	0.570731
6	-5.470152	-2.950881	-1.853352
6	-4.660844	-4.558273	0.317582
6	-5.115723	-4.293877	-2.039766
6	-4.709068	-5.088996	-0.969472
1	-4.359620	-5.199616	1.148480
1	-5.165020	-4.728777	-3.040065
1	-4.440478	-6.134440	-1.138475
6	-5.392010	2.430538	0.538082
6	-5.470204	2.950787	1.853390
6	-5.010775	3.223609	-0.570712
6	-5.115865	4.293806	2.039815
6	-4.661132	4.558286	-0.317552
6	-4.709324	5.088977	0.969517
1	-5.165146	4.728681	3.040125
1	-4.360006	5.199672	-1.148453
1	-4.440805	6.134439	1.138527
6	-5.003971	-2.715440	2.011227
1	-5.252202	-1.642224	2.003652
6	-5.950624	-2.127162	-3.047972
1	-6.046762	-1.079124	-2.719721
6	-7.340660	-2.596088	-3.523133
1	-7.710657	-1.951360	-4.335744
1	-7.300699	-3.627145	-3.908233
1	-8.084640	-2.582473	-2.711040
6	-4.951584	-2.139515	-4.219331
1	-3.949556	-1.796201	-3.916047
1	-4.839946	-3.146394	-4.650172
1	-5.302693	-1.477727	-5.026274
6	-3.618509	-2.849341	2.668461
1	-3.320932	-3.904555	2.771772
1	-2.839918	-2.335823	2.083713
1	-3.631318	-2.413836	3.680111
6	-6.086031	-3.418857	2.854140
1	-7.088430	-3.306324	2.411677
1	-5.886578	-4.498104	2.943996
1	-6.114232	-3.000953	3.872845
6	-5.950565	2.127012	3.048016
1	-6.046625	1.078970	2.719756
6	-7.340624	2.595813	3.523229
1	-8.084629	2.582140	2.711161
1	-7.710538	1.951045	4.335847
1	-7.300740	3.626870	3.908338
6	-4.951483	2.139438	4.219338
1	-4.839918	3.146322	4.650188
1	-5.302505	1.477609	5.026286
1	-3.949436	1.796217	3.916013
6	-5.004238	2.715480	-2.011224
1	-5.252343	1.642235	-2.003667
6	-6.086452	3.418803	-2.854017
1	-6.114695	3.000932	-3.872735
1	-7.088799	3.306139	-2.411470
1	-5.887132	4.498076	-2.943853
6	-3.618851	2.849576	-2.668575
1	-2.840144	2.336134	-2.083914
1	-3.631695	2.414105	-3.680240
1	-3.321414	3.904830	-2.771876
1	-7.604694	1.323678	-0.760589
1	-7.833919	-1.068636	-0.985894
1	7.833846	-1.442902	-0.176608
1	7.604046	0.630863	-1.390434

3-BH&HLYP

7	5.765693	1.030153	-0.379285
6	5.022815	-0.000001	0.000016
7	5.765690	-1.030156	0.379318
6	7.187889	0.670016	-0.362567
1	7.772855	1.435468	0.145240
6	7.187887	-0.670025	0.362596

6	3.550352	-0.000001	0.000012
6	2.841754	-1.115829	-0.445980
6	2.841752	1.115826	0.446000
6	1.458433	-1.108582	-0.454733
6	1.458431	1.108580	0.454744
6	0.740505	-0.000001	0.000004
1	3.364937	-1.994152	-0.794691
1	3.364933	1.994149	0.794712
1	0.932896	-1.991940	-0.796659
1	0.932892	1.991938	0.796667
1	7.545958	-0.595997	1.392859
6	5.347866	2.251229	-1.012650
6	4.956301	2.226100	-2.360054
6	5.431973	3.443980	-0.274879
6	4.605041	3.438251	-2.948646
6	5.076743	4.625196	-0.917875
6	4.661577	4.624073	-2.238666
1	4.295911	3.456260	-3.987093
1	5.131897	5.564000	-0.379676
1	4.392121	5.556741	-2.721390
6	5.347858	-2.251236	1.012673
6	5.431923	-3.443974	0.274876
6	4.956324	-2.226125	2.360086
6	5.076680	-4.625195	0.917856
6	4.605053	-3.438281	2.948662
6	4.661547	-4.624089	2.238657
1	5.131801	-5.563989	0.379636
1	4.295947	-3.456304	3.987116
1	4.392081	-5.556761	2.721368
6	5.918555	-3.493896	-1.164032
1	6.020464	-2.465186	-1.521145
6	4.928469	-4.193355	-2.095692
1	4.811357	-5.249300	-1.841086
1	3.936426	-3.734682	-2.063813
1	5.284604	-4.146399	-3.127801
6	7.295612	-4.156348	-1.256364
1	8.030988	-3.669662	-0.610508
1	7.246640	-5.206005	-0.955353
1	7.670050	-4.123247	-2.282769
6	4.934574	-0.963433	3.203621
1	5.192692	-0.115053	2.564663
6	5.987898	-1.022081	4.312002
1	6.005114	-0.084282	4.873529
1	5.775580	-1.826889	5.020253
1	6.989743	-1.195781	3.910625
6	3.546746	-0.682945	3.779432
1	2.789547	-0.628288	2.993595
1	3.242499	-1.460868	4.484196
1	3.546336	0.266006	4.322392
6	5.918648	3.493923	1.164014
1	6.020528	2.465220	1.521153
6	4.928616	4.193448	2.095683
1	3.936554	3.734814	2.063848
1	5.284780	4.146509	3.127783
1	4.811538	5.249390	1.841049
6	7.295733	4.156324	1.256289
1	7.670199	4.123237	2.282684
1	8.031071	3.669592	0.610425
1	7.246793	5.205975	0.955251
6	4.934504	0.963391	-3.203562
1	5.192629	0.115019	-2.564595
6	5.987793	1.021998	-4.311979
1	6.989654	1.195689	-3.910639
1	6.004975	0.084187	-4.873486
1	5.775466	1.826794	-5.020240
6	3.546652	0.682913	-3.779321
1	3.546210	-0.266049	-4.322262
1	2.789479	0.628284	-2.993459
1	3.242395	1.460825	-4.484092
6	-0.741355	-0.000001	0.000000
6	-1.459424	-0.605844	-1.033748
6	-1.459428	0.605842	1.033745
6	-2.842711	-0.614131	-1.032896
6	-2.842716	0.614127	1.032887
6	-3.551209	-0.000001	-0.000007
1	-0.934106	-1.061434	-1.864288
1	-0.934114	1.061433	1.864287

1	-3.365983	-1.084909	-1.852295
1	-3.365993	1.084904	1.852284
6	-5.023532	-0.000001	0.000003
7	-5.766193	1.048091	0.326541
6	-7.188451	0.752233	0.120882
6	-7.188455	-0.752236	-0.120778
7	-5.766206	-1.048092	-0.326502
1	-7.546773	-1.321285	0.741142
1	-7.773141	1.049634	0.990188
6	-5.347578	-2.402255	-0.566730
6	-4.958316	-3.202437	0.518539
6	-5.428753	-2.899355	-1.878338
6	-4.605855	-4.522252	0.248300
6	-5.072275	-4.227702	-2.086554
6	-4.659122	-5.030677	-1.037071
1	-4.298778	-5.168693	1.061988
1	-5.125088	-4.644922	-3.085139
1	-4.389093	-6.064439	-1.221345
6	-5.347568	2.402265	0.566714
6	-5.428653	2.899384	1.878321
6	-4.958404	3.202439	-0.518596
6	-5.072190	4.227742	2.086490
6	-4.605953	4.522267	-0.248403
6	-4.659137	5.030711	1.036964
1	-5.124934	4.644975	3.085074
1	-4.298953	5.168703	-1.062124
1	-4.389119	6.064483	1.221201
6	-4.940019	-2.714113	1.956490
1	-5.201450	-1.652841	1.965639
6	-5.914356	-2.063460	-3.051033
1	-6.016596	-1.029801	-2.708547
6	-7.291149	-2.533101	-3.528100
1	-7.665172	-1.882347	-4.322719
1	-7.241841	-3.549243	-3.927869
1	-8.026944	-2.539898	-2.719809
6	-4.923684	-2.051711	-4.215472
1	-3.931657	-1.707263	-3.910855
1	-4.806626	-3.044520	-4.656130
1	-5.279321	-1.386284	-5.006031
6	-3.552787	-2.838098	2.586214
1	-3.245320	-3.883442	2.671018
1	-2.795991	-2.312951	1.998579
1	-3.555249	-2.417051	3.595145
6	-5.992558	-3.438361	2.798560
1	-6.993986	-3.335372	2.372536
1	-5.776928	-4.507279	2.871663
1	-6.012842	-3.035640	3.814658
6	-5.914136	2.063492	3.051068
1	-6.016399	1.029830	2.708599
6	-7.290886	2.533122	3.528271
1	-8.026762	2.539912	2.720054
1	-7.664824	1.882366	4.322928
1	-7.241547	3.549265	3.928035
6	-4.923347	2.051761	4.215408
1	-4.806255	3.044574	4.656048
1	-5.278897	1.386334	5.006007
1	-3.931346	1.707323	3.910694
6	-4.940197	2.714099	-1.956542
1	-5.201634	1.652829	-1.965665
6	-5.992781	3.438338	-2.798562
1	-6.013122	3.035602	-3.814653
1	-6.994185	3.335356	-2.372481
1	-5.777155	4.507254	-2.871695
6	-3.553000	2.838076	-2.586346
1	-2.796169	2.312939	-1.998745
1	-3.555516	2.417014	-3.595271
1	-3.245540	3.883419	-2.671181
1	-7.546808	1.321283	-0.741021
1	-7.773184	-1.049641	-0.990057
1	7.772849	-1.435479	-0.145213
1	7.545956	0.595988	-1.392831

4-Singlet-B3LYP

7	-3.742638	1.102194	-0.236234
6	-2.890747	0.000139	0.000104
7	-3.742660	-1.101881	0.236507
6	-5.134934	0.759065	0.043401

1	-5.439093	1.107267	1.049830
6	-5.134990	-0.758650	-0.042855
6	-1.500300	0.000101	0.000030
6	-0.695965	-1.157951	0.382773
6	-0.695901	1.158084	-0.382785
6	0.665855	-1.161525	0.371269
6	0.665920	1.161546	-0.371382
6	1.461384	-0.000021	-0.000082
1	-1.189404	-2.067120	0.715291
1	-1.189294	2.067277	-0.715317
1	1.174140	-2.069646	0.691398
1	1.174254	2.069602	-0.691615
1	-5.815238	-1.227494	0.686097
6	-3.427598	2.498711	-0.139370
6	-3.167122	3.100729	1.117102
6	-3.480733	3.277705	-1.323075
6	-2.927709	4.482186	1.154654
6	-3.242373	4.656026	-1.229626
6	-2.962868	5.256518	-0.003382
1	-2.712773	4.962571	2.112435
1	-3.272051	5.269850	-2.133538
1	-2.774390	6.332019	0.050709
6	-3.427797	-2.498430	0.139519
6	-3.480899	-3.277497	1.323170
6	-3.167554	-3.100395	-1.117032
6	-3.242778	-4.655853	1.229591
6	-2.928373	-4.481885	-1.154714
6	-2.963525	-5.256300	0.003270
1	-3.272443	-5.269734	2.133464
1	-2.713648	-4.962241	-2.112559
1	-2.775232	-6.331828	-0.050930
6	-3.769570	-2.652941	2.685339
1	-3.913558	-1.575954	2.515870
6	-2.580196	-2.806778	3.650169
1	-2.377279	-3.865889	3.880408
1	-1.662248	-2.371528	3.226198
1	-2.788392	-2.294984	4.604361
6	-5.063754	-3.206708	3.307758
1	-5.926078	-3.076559	2.634385
1	-4.979566	-4.283082	3.531833
1	-5.291698	-2.689482	4.254387
6	-3.136267	-2.305599	-2.420411
1	-3.327905	-1.251303	-2.177311
6	-4.238506	-2.764646	-3.393247
1	-4.240245	-2.136008	-4.298945
1	-4.086029	-3.807665	-3.715714
1	-5.239078	-2.704090	-2.935806
6	-1.751175	-2.360665	-3.087541
1	-0.966219	-2.021107	-2.396873
1	-1.498558	-3.383596	-3.412430
1	-1.727448	-1.714035	-3.979975
6	-3.769680	2.653090	-2.685155
1	-3.913804	1.576137	-2.515586
6	-2.580436	2.806686	-3.650177
1	-1.662475	2.371361	-3.226307
1	-2.788837	2.294836	-4.604294
1	-2.377411	3.865751	-3.880545
6	-5.063894	3.206991	-3.307418
1	-5.292016	2.689770	-4.254006
1	-5.926131	3.076944	-2.633917
1	-4.979607	4.283351	-3.531517
6	-3.135794	2.306032	2.420540
1	-3.327527	1.251731	2.177537
6	-4.237933	2.765247	3.393414
1	-5.238543	2.704727	2.936052
1	-4.239656	2.136699	4.299174
1	-4.085347	3.808287	3.715761
6	-1.750656	2.361034	3.087577
1	-1.726923	1.714439	3.980036
1	-0.965773	2.021388	2.396873
1	-1.497939	3.383957	3.412411
6	2.846030	-0.000080	-0.000124
7	3.680912	1.134450	-0.023247
6	5.076743	0.733063	-0.225261
6	5.076698	-0.733455	0.224789
7	3.680801	-1.134688	0.022926
1	5.382276	-0.827934	1.284417

1	5.749394	1.361644	0.375606
6	3.392436	-2.390224	-0.602975
6	3.167606	-2.475614	-2.001136
6	3.392563	-3.558539	0.202610
6	2.905243	-3.737124	-2.556182
6	3.152314	-4.797637	-0.406818
6	2.898386	-4.890060	-1.773747
1	2.721652	-3.820116	-3.630449
1	3.152946	-5.705777	0.200947
1	2.701107	-5.862984	-2.231597
6	3.392752	2.389952	0.602820
6	3.392945	3.558363	-0.202634
6	3.168085	2.475207	2.001009
6	3.152916	4.797421	0.406964
6	2.905948	3.736687	2.556231
6	2.899151	4.889717	1.773932
1	3.153601	5.705629	-0.200700
1	2.722491	3.819584	3.630528
1	2.702050	5.862609	2.231925
6	3.253747	-1.267895	-2.932043
1	3.463081	-0.382739	-2.318751
6	4.418975	-1.414299	-3.929245
1	5.377751	-1.578502	-3.411351
1	4.264891	-2.263336	-4.615089
1	4.516949	-0.505096	-4.545353
6	1.927596	-0.993574	-3.660037
1	2.014658	-0.093716	-4.291144
1	1.641540	-1.831765	-4.316737
1	1.111793	-0.826411	-2.942320
6	3.655637	-3.492143	1.704990
1	3.617622	-2.428940	1.986143
6	2.580866	-4.218762	2.531452
1	1.572070	-3.841965	2.302699
1	2.583202	-5.305665	2.348551
1	2.760795	-4.069003	3.608670
6	5.060557	-4.019949	2.052716
1	5.264269	-3.912745	3.131146
1	5.157954	-5.088690	1.798624
1	5.846452	-3.478099	1.503698
6	3.655876	3.492115	-1.705044
1	3.617606	2.428959	-1.986337
6	2.581214	4.219087	-2.531331
1	2.761037	4.069425	-3.608581
1	1.572345	3.842495	-2.302560
1	2.583811	5.305968	-2.348301
6	5.060888	4.019666	-2.052806
1	5.846713	3.477571	-1.503931
1	5.264491	3.912579	-3.131267
1	5.158520	5.088349	-1.798559
6	3.254201	1.267368	2.931758
1	3.463262	0.382247	2.318322
6	4.419657	1.413476	3.928739
1	4.517596	0.504206	4.544754
1	5.378357	1.577550	3.410662
1	4.265863	2.262493	4.614674
6	1.928157	0.993159	3.659985
1	1.112183	0.826233	2.942411
1	2.015208	0.093192	4.290935
1	1.642368	1.831290	4.316878
1	5.749207	-1.362093	-0.376179
1	5.382193	0.827483	-1.284931
1	-5.815284	1.227943	-0.685434
1	-5.439355	-1.106831	-1.049230

4-Triplet-B3LYP

7	-3.655548	-1.020093	0.519947
6	-2.838073	0.000832	0.000125
7	-3.654589	1.022101	-0.520472
6	-5.050325	-0.754239	0.142919
1	-5.342845	-1.329525	-0.755644
6	-5.049713	0.757296	-0.143995
6	-1.393116	0.000327	0.000313
6	-0.639466	1.204317	0.018569
6	-0.640154	-1.204093	-0.017765
6	0.751582	1.204522	0.011162
6	0.750889	-1.205079	-0.010114
6	1.507645	-0.000496	0.000564

1	-1.161006	2.161378	0.040179
1	-1.162234	-2.160855	-0.039444
1	1.268786	2.163112	0.006604
1	1.267566	-2.163953	-0.005446
1	-5.731340	1.034538	-0.960225
6	-3.358811	-1.771902	1.703438
6	-3.350759	-3.188954	1.605934
6	-3.110602	-1.132769	2.947334
6	-3.085817	-3.943461	2.757181
6	-2.828156	-1.934846	4.062657
6	-2.815746	-3.326235	3.976535
1	-3.080863	-5.034469	2.696372
1	-2.636400	-1.459303	5.027571
1	-2.602016	-3.929216	4.862994
6	-3.356798	1.773476	-1.703956
6	-3.108149	1.133920	-2.947552
6	-3.348045	3.190544	-1.606784
6	-2.824579	1.935582	-4.062887
6	-3.082037	3.944640	-2.758050
6	-2.811526	3.326988	-3.977091
1	-2.632442	1.459705	-5.027557
1	-3.076567	5.035660	-2.697490
1	-2.596950	3.929652	-4.863562
6	-3.233030	-0.376857	-3.141225
1	-3.294997	-0.840959	-2.148839
6	-2.013461	-1.001328	-3.837200
1	-1.889820	-0.631441	-4.868320
1	-1.086892	-0.788858	-3.285087
1	-2.127240	-2.096151	-3.895438
6	-4.533936	-0.720657	-3.893327
1	-5.420314	-0.314468	-3.380002
1	-4.528102	-0.306755	-4.915202
1	-4.659849	-1.813182	-3.975370
6	-3.637288	3.901418	-0.287170
1	-3.589549	3.134537	0.500898
6	-5.057087	4.499744	-0.270983
1	-5.278378	4.960738	0.706091
1	-5.167251	5.280303	-1.042237
1	-5.823908	3.734005	-0.465880
6	-2.592662	4.976709	0.056884
1	-1.570685	4.566865	0.048064
1	-2.619971	5.821718	-0.650195
1	-2.782923	5.387566	1.061887
6	-3.234677	0.378025	3.141301
1	-3.296193	0.842379	2.149003
6	-2.014808	1.001592	3.837586
1	-1.088340	0.788781	3.285434
1	-2.127976	2.096457	3.896207
1	-1.891416	0.631249	4.868574
6	-4.535502	0.722455	3.893244
1	-4.660733	1.815042	3.975498
1	-5.422015	0.316942	3.379620
1	-4.530151	0.308330	4.915029
6	-3.639708	-3.899349	0.286003
1	-3.591021	-3.132319	-0.501860
6	-5.059920	-4.496686	0.268904
1	-5.826300	-3.730447	0.463573
1	-5.281014	-4.957286	-0.708401
1	-5.171047	-5.277347	1.039916
6	-2.595632	-4.975277	-0.057712
1	-2.785653	-5.385813	-1.062889
1	-1.573388	-4.566113	-0.048291
1	-2.623875	-5.820398	0.649195
6	2.951934	-0.000969	0.000577
7	3.779681	-1.015355	-0.533009
6	5.169921	-0.551844	-0.528757
6	5.170271	0.548127	0.530116
7	3.780459	1.012932	0.533923
1	5.870081	1.364644	0.291589
1	5.456298	-0.149338	-1.519273
6	3.397268	1.874404	1.618329
6	3.024681	1.352057	2.884322
6	3.451319	3.275904	1.400647
6	2.667836	2.255520	3.896399
6	3.101759	4.134779	2.451866
6	2.701857	3.633201	3.688690
1	2.371797	1.870975	4.875623

1	3.138074	5.216205	2.297839
1	2.424221	4.316704	4.495477
6	3.395874	-1.876052	-1.617804
6	3.448638	-3.277695	-1.400737
6	3.023884	-1.352808	-2.883611
6	3.098395	-4.135790	-2.452372
6	2.666312	-2.255495	-3.896114
6	2.699061	-3.633304	-3.689005
1	3.133699	-5.217316	-2.298816
1	2.370712	-1.870262	-4.875200
1	2.420878	-4.316194	-4.496122
6	3.052602	-0.141250	3.211716
1	3.312399	-0.686740	2.294544
6	4.138447	-0.458446	4.258662
1	5.132116	-0.108798	3.935610
1	3.919964	0.020993	5.226911
1	4.200580	-1.545340	4.433329
6	1.682401	-0.670995	3.666274
1	1.733728	-1.755920	3.854169
1	1.347942	-0.190601	4.600284
1	0.914871	-0.500321	2.898369
6	3.894799	3.864724	0.063747
1	3.994865	3.020434	-0.634279
6	2.858438	4.833814	-0.530829
1	1.873583	4.354597	-0.644118
1	2.724316	5.729746	0.097239
1	3.180964	5.177786	-1.527296
6	5.272753	4.543601	0.178936
1	5.616396	4.901327	-0.805910
1	5.236311	5.413830	0.855392
1	6.034542	3.852740	0.573607
6	3.891441	-3.867509	-0.064052
1	3.991846	-3.023666	0.634468
6	2.854403	-4.836337	0.529754
1	3.176463	-5.180946	1.526151
1	1.869761	-4.356666	0.642980
1	2.719997	-5.731908	-0.098767
6	5.269029	-4.547135	-0.179311
1	6.031311	-3.856499	-0.573418
1	5.612240	-4.905635	0.805403
1	5.232209	-5.416941	-0.856290
6	3.053267	0.140620	-3.210341
1	3.313450	0.685453	-2.292891
6	4.139589	0.457254	-4.256962
1	4.202789	1.544167	-4.431124
1	5.132871	0.106510	-3.933905
1	3.920808	-0.021536	-5.225463
6	1.683651	0.671884	-3.664903
1	0.915805	0.501654	-2.897214
1	1.736060	1.756834	-3.852354
1	1.348897	0.192192	-4.599167
1	5.455983	0.145332	1.520711
1	5.868929	-1.368985	-0.289997
1	-5.732490	-1.030947	0.958887
1	-5.342155	1.332756	0.754483

4-Singlet-Open-Shell-B3LYP $\langle S^2 \rangle$= 0.0000

7	-3.742638	1.102194	-0.236234
6	-2.890747	0.000139	0.000104
7	-3.742660	-1.101881	0.236507
6	-5.134934	0.759065	0.043401
1	-5.439093	1.107267	1.049830
6	-5.134990	-0.758650	-0.042855
6	-1.500300	0.000101	0.000030
6	-0.695965	-1.157951	0.382773
6	-0.695901	1.158084	-0.382785
6	0.665855	-1.161525	0.371269
6	0.665920	1.161546	-0.371382
6	1.461384	-0.000021	-0.000082
1	-1.189404	-2.067120	0.715291
1	-1.189294	2.067277	-0.715317
1	1.174140	-2.069646	0.691398
1	1.174254	2.069602	-0.691615
1	-5.815238	-1.227494	0.686097
6	-3.427598	2.498711	-0.139370
6	-3.167122	3.100729	1.117102
6	-3.480733	3.277705	-1.323075

6	-2.927709	4.482186	1.154654
6	-3.242373	4.656026	-1.229626
6	-2.962868	5.256518	-0.003382
1	-2.712773	4.962571	2.112435
1	-3.272051	5.269850	-2.133538
1	-2.774390	6.332019	0.050709
6	-3.427797	-2.498430	0.139519
6	-3.480899	-3.277497	1.323170
6	-3.167554	-3.100395	-1.117032
6	-3.242778	-4.655853	1.229591
6	-2.928373	-4.481885	-1.154714
6	-2.963525	-5.256300	0.003270
1	-3.272443	-5.269734	2.133464
1	-2.713648	-4.962241	-2.112559
1	-2.775232	-6.331828	-0.050930
6	-3.769570	-2.652941	2.685339
1	-3.913558	-1.575954	2.515870
6	-2.580196	-2.806778	3.650169
1	-2.377279	-3.865889	3.880408
1	-1.662248	-2.371528	3.226198
1	-2.788392	-2.294984	4.604361
6	-5.063754	-3.206708	3.307758
1	-5.926078	-3.076559	2.634385
1	-4.979566	-4.283082	3.531833
1	-5.291698	-2.689482	4.254387
6	-3.136267	-2.305599	-2.420411
1	-3.327905	-1.251303	-2.177311
6	-4.238506	-2.764646	-3.393247
1	-4.240245	-2.136008	-4.298945
1	-4.086029	-3.807665	-3.715714
1	-5.239078	-2.704090	-2.935806
6	-1.751175	-2.360665	-3.087541
1	-0.966219	-2.021107	-2.396873
1	-1.498558	-3.383596	-3.412430
1	-1.727448	-1.714035	-3.979975
6	-3.769680	2.653090	-2.685155
1	-3.913804	1.576137	-2.515586
6	-2.580436	2.806686	-3.650177
1	-1.662475	2.371361	-3.226307
1	-2.788837	2.294836	-4.604294
1	-2.377411	3.865751	-3.880545
6	-5.063894	3.206991	-3.307418
1	-5.292016	2.689770	-4.254006
1	-5.926131	3.076944	-2.633917
1	-4.979607	4.283351	-3.531517
6	-3.135794	2.306032	2.420540
1	-3.327527	1.251731	2.177537
6	-4.237933	2.765247	3.393414
1	-5.238543	2.704727	2.936052
1	-4.239656	2.136699	4.299174
1	-4.085347	3.808287	3.715761
6	-1.750656	2.361034	3.087577
1	-1.726923	1.714439	3.980036
1	-0.965773	2.021388	2.396873
1	-1.497939	3.383957	3.412411
6	2.846030	-0.000080	-0.000124
7	3.680912	1.134450	-0.023247
6	5.076743	0.733063	-0.225261
6	5.076698	-0.733455	0.224789
7	3.680801	-1.134688	0.022926
1	5.382276	-0.827934	1.284417
1	5.749394	1.361644	0.375606
6	3.392436	-2.390224	-0.602975
6	3.167606	-2.475614	-2.001136
6	3.392563	-3.558539	0.202610
6	2.905243	-3.737124	-2.556182
6	3.152314	-4.797637	-0.406818
6	2.898386	-4.890060	-1.773747
1	2.721652	-3.820116	-3.630449
1	3.152946	-5.705777	0.200947
1	2.701107	-5.862984	-2.231597
6	3.392752	2.389952	0.602820
6	3.392945	3.558363	-0.202634
6	3.168085	2.475207	2.001009
6	3.152916	4.797421	0.406964
6	2.905948	3.736687	2.556231
6	2.899151	4.889717	1.773932

1	3.153601	5.705629	-0.200700
1	2.722491	3.819584	3.630528
1	2.702050	5.862609	2.231925
6	3.253747	-1.267895	-2.932043
1	3.463081	-0.382739	-2.318751
6	4.418975	-1.414299	-3.929245
1	5.377751	-1.578502	-3.411351
1	4.264891	-2.263336	-4.615089
1	4.516949	-0.505096	-4.545353
6	1.927596	-0.993574	-3.660037
1	2.014658	-0.093716	-4.291144
1	1.641540	-1.831765	-4.316737
1	1.111793	-0.826411	-2.942320
6	3.655637	-3.492143	1.704990
1	3.617622	-2.428940	1.986143
6	2.580866	-4.218762	2.531452
1	1.572070	-3.841965	2.302699
1	2.583202	-5.305665	2.348551
1	2.760795	-4.069003	3.608670
6	5.060557	-4.019949	2.052716
1	5.264269	-3.912745	3.131146
1	5.157954	-5.088690	1.798624
1	5.846452	-3.478099	1.503698
6	3.655876	3.492115	-1.705044
1	3.617606	2.428959	-1.986337
6	2.581214	4.219087	-2.531331
1	2.761037	4.069425	-3.608581
1	1.572345	3.842495	-2.302560
1	2.583811	5.305968	-2.348301
6	5.060888	4.019666	-2.052806
1	5.846713	3.477571	-1.503931
1	5.264491	3.912579	-3.131267
1	5.158520	5.088349	-1.798559
6	3.254201	1.267368	2.931758
1	3.463262	0.382247	2.318322
6	4.419657	1.413476	3.928739
1	4.517596	0.504206	4.544754
1	5.378357	1.577550	3.410662
1	4.265863	2.262493	4.614674
6	1.928157	0.993159	3.659985
1	1.112183	0.826233	2.942411
1	2.015208	0.093192	4.290935
1	1.642368	1.831290	4.316878
1	5.749207	-1.362093	-0.376179
1	5.382193	0.827483	-1.284931
1	-5.815284	1.227943	-0.685434
1	-5.439355	-1.106831	-1.049230

4-Singlet-BH&HLYP

7	-3.709918	1.094894	-0.218426
6	-2.863466	-0.000123	-0.000047
7	-3.709787	-1.095238	0.218264
6	-5.092835	0.753337	0.047822
1	-5.399452	1.092582	1.046234
6	-5.092745	-0.753849	-0.047982
6	-1.490924	-0.000051	-0.000028
6	-0.686399	-1.158725	0.372221
6	-0.686478	1.158678	-0.372273
6	0.660103	-1.162148	0.360657
6	0.660024	1.162209	-0.360641
6	1.455571	0.000067	0.000031
1	-1.174980	-2.063782	0.695294
1	-1.175110	2.063670	-0.695448
1	1.163513	-2.065651	0.671878
1	1.163375	2.065731	-0.671905
1	-5.762511	-1.223987	0.676866
6	-3.393824	2.478417	-0.092660
6	-3.136653	3.044804	1.167533
6	-3.435175	3.276624	-1.249158
6	-2.886291	4.412649	1.238321
6	-3.186762	4.640571	-1.126415
6	-2.908326	5.206827	0.105359
1	-2.672473	4.865377	2.200216
1	-3.207245	5.269279	-2.009965
1	-2.711010	6.270664	0.183355
6	-3.393528	-2.478724	0.092536
6	-3.434927	-3.276945	1.249017

6	-3.136191	-3.045079	-1.167648
6	-3.186427	-4.640879	1.126276
6	-2.885749	-4.412906	-1.238431
6	-2.907849	-5.207100	-0.105478
1	-3.206954	-5.269600	2.009815
1	-2.671822	-4.865615	-2.200310
1	-2.710473	-6.270926	-0.183472
6	-3.723390	-2.688222	2.617460
1	-3.874258	-1.617272	2.475552
6	-2.539674	-2.857177	3.569545
1	-2.333002	-3.912613	3.771707
1	-1.632083	-2.409906	3.157186
1	-2.748382	-2.372781	4.528322
6	-5.003175	-3.263666	3.223353
1	-5.859924	-3.124007	2.558387
1	-4.909459	-4.336387	3.417979
1	-5.231290	-2.775120	4.175499
6	-3.116918	-2.223467	-2.444145
1	-3.332726	-1.188048	-2.179965
6	-4.195606	-2.682198	-3.426101
1	-4.207787	-2.036121	-4.309069
1	-4.017004	-3.705186	-3.769970
1	-5.191125	-2.656772	-2.974295
6	-1.736640	-2.233086	-3.098477
1	-0.970440	-1.893512	-2.400118
1	-1.462568	-3.236234	-3.439375
1	-1.722683	-1.571939	-3.970162
6	-3.723437	2.687869	-2.617631
1	-3.874299	1.616918	-2.475727
6	-2.539578	2.856830	-3.569539
1	-1.632051	2.409567	-3.157032
1	-2.748127	2.372433	-4.528349
1	-2.332878	3.912268	-3.771665
6	-5.003149	3.263266	-3.223717
1	-5.231118	2.774681	-4.175879
1	-5.859984	3.123601	-2.558863
1	-4.909439	4.335984	-3.418366
6	-3.117473	2.223218	2.444047
1	-3.333181	1.187777	2.179861
6	-4.196320	2.681901	3.425854
1	-5.191778	2.656394	2.973920
1	-4.208570	2.035849	4.308840
1	-4.017832	3.704913	3.769712
6	-1.737279	2.232936	3.098545
1	-1.723374	1.571776	3.970220
1	-0.970976	1.893435	2.400264
1	-1.463321	3.236096	3.439495
6	2.821916	0.000126	0.000057
7	3.652561	1.123560	-0.033005
6	5.039661	0.729968	-0.217736
6	5.039685	-0.729482	0.218271
7	3.652683	-1.123202	0.033133
1	5.356249	-0.833813	1.264215
1	5.697846	1.350797	0.392210
6	3.361473	-2.379979	-0.566390
6	3.155155	-2.484929	-1.953540
6	3.340334	-3.523732	0.252355
6	2.888040	-3.742926	-2.488673
6	3.095427	-4.761945	-0.333163
6	2.859084	-4.873655	-1.692124
1	2.715484	-3.840507	-3.554946
1	3.078876	-5.652475	0.285156
1	2.657775	-5.844505	-2.132216
6	3.361071	2.380297	0.566497
6	3.339765	3.524033	-0.252272
6	3.154620	2.485224	1.953624
6	3.094531	4.762198	0.333203
6	2.887199	3.743175	2.488720
6	2.858053	4.873877	1.692145
1	3.077838	5.652717	-0.285128
1	2.714534	3.840738	3.554977
1	2.656495	5.844693	2.132201
6	3.251407	-1.295586	-2.892532
1	3.497133	-0.417373	-2.297907
6	4.378785	-1.479225	-3.909668
1	5.337194	-1.665583	-3.416799
1	4.185144	-2.320490	-4.581421

1	4.484768	-0.582667	-4.528038
6	1.922742	-0.999908	-3.584546
1	2.014682	-0.117332	-4.224953
1	1.603803	-1.834892	-4.215917
1	1.136948	-0.806078	-2.852793
6	3.587057	-3.431830	1.747200
1	3.551824	-2.372350	2.007845
6	2.508482	-4.135826	2.568555
1	1.511219	-3.762358	2.323921
1	2.510480	-5.217307	2.405141
1	2.677066	-3.968174	3.636419
6	4.976140	-3.957967	2.112354
1	5.169814	-3.833558	3.182266
1	5.067249	-5.023328	1.878669
1	5.762197	-3.433248	1.563397
6	3.586788	3.432138	-1.747064
1	3.551572	2.372656	-2.007709
6	2.508469	4.136211	-2.568680
1	2.677258	3.968470	-3.636497
1	1.511105	3.762886	-2.324237
1	2.510569	5.217704	-2.405348
6	4.975993	3.958203	-2.111884
1	5.761892	3.433430	-1.562754
1	5.169912	3.833808	-3.181752
1	5.067095	5.023554	-1.878145
6	3.250992	1.295906	2.892634
1	3.496801	0.417709	2.298027
6	4.378347	1.479696	3.909770
1	4.484406	0.583190	4.528202
1	5.336744	1.666108	3.416899
1	4.184624	2.320994	4.581457
6	1.922358	1.000071	3.584636
1	1.136597	0.806166	2.852866
1	2.014396	0.117487	4.225017
1	1.603309	1.834995	4.216030
1	5.698099	-1.350231	-0.391509
1	5.356488	0.834320	-1.263598
1	-5.762656	1.223399	-0.677024
1	-5.399321	-1.093140	-1.046392

4-Triplet-BH&HLYP

7	3.663873	-1.051491	-0.738138
6	2.859102	-0.109995	-0.089570
7	3.712119	0.817613	0.517720
6	5.052414	-0.860977	-0.359551
1	5.334389	-1.544929	0.451980
6	5.082648	0.579699	0.100083
6	1.451159	-0.089955	-0.057258
6	0.726333	0.696620	0.888290
6	0.656030	-0.846232	-0.967157
6	-0.649303	0.723055	0.903148
6	-0.721255	-0.798085	-0.934109
6	-1.422081	-0.014775	-0.009154
1	1.258519	1.277251	1.626027
1	1.128750	-1.458833	-1.719013
1	-1.149387	1.331368	1.648690
1	-1.282348	-1.381263	-1.655419
1	5.785039	0.747563	0.920046
6	3.288925	-2.383738	-1.084347
6	2.929052	-3.319668	-0.098926
6	3.356172	-2.752289	-2.440257
6	2.601370	-4.611130	-0.504911
6	3.032338	-4.058711	-2.793547
6	2.649434	-4.982175	-1.836702
1	2.308869	-5.343199	0.239498
1	3.075116	-4.356835	-3.835424
1	2.392380	-5.994734	-2.128772
6	3.384470	2.172109	0.825361
6	3.517370	2.591572	2.161490
6	3.004826	3.077705	-0.181322
6	3.239523	3.918419	2.475643
6	2.722922	4.391269	0.186899
6	2.836269	4.812854	1.499807
1	3.334532	4.255894	3.501941
1	2.416526	5.100961	-0.573392
1	2.615253	5.842046	1.761525
6	3.949548	1.639373	3.262097

1	4.055795	0.654184	2.806020
6	2.897904	1.517406	4.364321
1	2.743818	2.467607	4.884157
1	1.933657	1.195519	3.963303
1	3.211802	0.781739	5.110855
6	5.306854	2.034350	3.844703
1	6.073924	2.099415	3.068280
1	5.260673	3.007872	4.342184
1	5.636960	1.299950	4.585629
6	2.912712	2.695695	-1.649110
1	3.153567	1.636676	-1.739995
6	3.934755	3.466522	-2.486861
1	3.904235	3.131711	-3.528052
1	3.730938	4.541193	-2.480730
1	4.953046	3.323236	-2.114699
6	1.500350	2.880048	-2.203469
1	0.768334	2.309798	-1.629377
1	1.195981	3.930626	-2.187828
1	1.453152	2.540457	-3.242276
6	3.763870	-1.766997	-3.520770
1	3.929992	-0.807440	-3.029441
6	2.658345	-1.560333	-4.555534
1	1.731044	-1.222753	-4.086300
1	2.957587	-0.804553	-5.287920
1	2.440003	-2.481460	-5.103958
6	5.072847	-2.180073	-4.193962
1	5.388845	-1.423511	-4.918538
1	5.877456	-2.306172	-3.464395
1	4.965901	-3.127274	-4.731251
6	2.901201	-2.990557	1.383744
1	3.160796	-1.938541	1.502374
6	3.942385	-3.806048	2.152612
1	4.947873	-3.662832	1.747220
1	3.956512	-3.510652	3.206036
1	3.722099	-4.876801	2.113706
6	1.508437	-3.175593	1.984790
1	1.504673	-2.870809	3.035281
1	0.763776	-2.578031	1.456353
1	1.188874	-4.221051	1.945623
6	-2.906573	0.018662	0.007938
7	-3.633975	-0.673088	1.003067
6	-4.830869	0.044209	1.383669
6	-4.956668	1.085809	0.280095
7	-3.585016	1.252090	-0.149794
1	-5.379233	2.028254	0.635300
1	-4.730116	0.526538	2.366548
6	-3.272028	2.219226	-1.144630
6	-3.374075	1.932879	-2.520060
6	-2.900631	3.509522	-0.716299
6	-3.074525	2.937184	-3.437445
6	-2.623186	4.483765	-1.671239
6	-2.701762	4.203188	-3.023372
1	-3.147789	2.726431	-4.498609
1	-2.338265	5.479579	-1.349843
1	-2.478852	4.972704	-3.754971
6	-3.336156	-1.970937	1.482399
6	-3.582205	-3.100213	0.676230
6	-2.831303	-2.127920	2.787226
6	-3.307610	-4.364026	1.190891
6	-2.587697	-3.413192	3.265161
6	-2.817613	-4.525018	2.475365
1	-3.494318	-5.240740	0.581033
1	-2.205178	-3.545248	4.271313
1	-2.618100	-5.519045	2.861657
6	-3.847520	0.588296	-3.041650
1	-3.894087	-0.092279	-2.191789
6	-5.251396	0.691075	-3.640242
1	-5.967222	1.094386	-2.919391
1	-5.263058	1.346468	-4.516603
1	-5.608690	-0.293875	-3.957027
6	-2.874001	-0.023301	-4.047917
1	-3.189862	-1.036480	-4.314871
1	-2.827660	0.557028	-4.974181
1	-1.863615	-0.079072	-3.637683
6	-2.819561	3.874175	0.754183
1	-2.953301	2.947408	1.313014
6	-1.459177	4.454471	1.136510

1	-0.644131	3.786038	0.851101
1	-1.281192	5.421421	0.656283
1	-1.403841	4.614820	2.217745
6	-3.947627	4.826290	1.154949
1	-3.918999	5.029321	2.230084
1	-3.862429	5.785078	0.634011
1	-4.930102	4.410473	0.916227
6	-4.197755	-2.976570	-0.705099
1	-4.112269	-1.931775	-1.001044
6	-3.467012	-3.803900	-1.760015
1	-3.871267	-3.591774	-2.754422
1	-2.397984	-3.578151	-1.773148
1	-3.577888	-4.878801	-1.589753
6	-5.685805	-3.328861	-0.670717
1	-6.227281	-2.704814	0.045048
1	-6.141570	-3.186100	-1.655650
1	-5.838600	-4.372707	-0.379017
6	-2.566041	-0.937747	3.689953
1	-2.680389	-0.043134	3.077132
6	-3.586937	-0.863820	4.826565
1	-3.428020	0.033191	5.433204
1	-4.611394	-0.838363	4.446425
1	-3.503829	-1.731433	5.488506
6	-1.140564	-0.926894	4.238531
1	-0.407537	-0.964035	3.430239
1	-0.963829	-0.013935	4.815812
1	-0.954452	-1.773990	4.905391
1	-5.599984	0.716221	-0.532928
1	-5.702168	-0.615681	1.430324
1	5.724003	-1.051290	-1.200067
1	5.362792	1.253792	-0.720021

4-Singlet-Open-Shell-BH&HLYP $\langle S^2 \rangle$= 0.0000

7	-3.709918	1.094894	-0.218426
6	-2.863466	-0.000123	-0.000047
7	-3.709787	-1.095238	0.218264
6	-5.092835	0.753337	0.047822
1	-5.399452	1.092582	1.046234
6	-5.092745	-0.753849	-0.047982
6	-1.490924	-0.000051	-0.000028
6	-0.686399	-1.158725	0.372221
6	-0.686478	1.158678	-0.372273
6	0.660103	-1.162148	0.360657
6	0.660024	1.162209	-0.360641
6	1.455571	0.000067	0.000031
1	-1.174980	-2.063782	0.695294
1	-1.175110	2.063670	-0.695448
1	1.163513	-2.065651	0.671878
1	1.163375	2.065731	-0.671905
1	-5.762511	-1.223987	0.676866
6	-3.393824	2.478417	-0.092660
6	-3.136653	3.044804	1.167533
6	-3.435175	3.276624	-1.249158
6	-2.886291	4.412649	1.238321
6	-3.186762	4.640571	-1.126415
6	-2.908326	5.206827	0.105359
1	-2.672473	4.865377	2.200216
1	-3.207245	5.269279	-2.009965
1	-2.711010	6.270664	0.183355
6	-3.393528	-2.478724	0.092536
6	-3.434927	-3.276945	1.249017
6	-3.136191	-3.045079	-1.167648
6	-3.186427	-4.640879	1.126276
6	-2.885749	-4.412906	-1.238431
6	-2.907849	-5.207100	-0.105478
1	-3.206954	-5.269600	2.009815
1	-2.671822	-4.865615	-2.200310
1	-2.710473	-6.270926	-0.183472
6	-3.723390	-2.688222	2.617460
1	-3.874258	-1.617272	2.475552
6	-2.539674	-2.857177	3.569545
1	-2.333002	-3.912613	3.771707
1	-1.632083	-2.409906	3.157186
1	-2.748382	-2.372781	4.528322
6	-5.003175	-3.263666	3.223353
1	-5.859924	-3.124007	2.558387
1	-4.909459	-4.336387	3.417979

1	-5.231290	-2.775120	4.175499
6	-3.116918	-2.223467	-2.444145
1	-3.332726	-1.188048	-2.179965
6	-4.195606	-2.682198	-3.426101
1	-4.207787	-2.036121	-4.309069
1	-4.017004	-3.705186	-3.769970
1	-5.191125	-2.656772	-2.974295
6	-1.736640	-2.233086	-3.098477
1	-0.970440	-1.893512	-2.400118
1	-1.462568	-3.236234	-3.439375
1	-1.722683	-1.571939	-3.970162
6	-3.723437	2.687869	-2.617631
1	-3.874299	1.616918	-2.475727
6	-2.539578	2.856830	-3.569539
1	-1.632051	2.409567	-3.157032
1	-2.748127	2.372433	-4.528349
1	-2.332878	3.912268	-3.771665
6	-5.003149	3.263266	-3.223717
1	-5.231118	2.774681	-4.175879
1	-5.859984	3.123601	-2.558863
1	-4.909439	4.335984	-3.418366
6	-3.117473	2.223218	2.444047
1	-3.333181	1.187777	2.179861
6	-4.196320	2.681901	3.425854
1	-5.191778	2.656394	2.973920
1	-4.208570	2.035849	4.308840
1	-4.017832	3.704913	3.769712
6	-1.737279	2.232936	3.098545
1	-1.723374	1.571776	3.970220
1	-0.970976	1.893435	2.400264
1	-1.463321	3.236096	3.439495
6	2.821916	0.000126	0.000057
7	3.652561	1.123560	-0.033005
6	5.039661	0.729968	-0.217736
6	5.039685	-0.729482	0.218271
7	3.652683	-1.123202	0.033133
1	5.356249	-0.833813	1.264215
1	5.697846	1.350797	0.392210
6	3.361473	-2.379979	-0.566390
6	3.155155	-2.484929	-1.953540
6	3.340334	-3.523732	0.252355
6	2.888040	-3.742926	-2.488673
6	3.095427	-4.761945	-0.333163
6	2.859084	-4.873655	-1.692124
1	2.715484	-3.840507	-3.554946
1	3.078876	-5.652475	0.285156
1	2.657775	-5.844505	-2.132216
6	3.361071	2.380297	0.566497
6	3.339765	3.524033	-0.252272
6	3.154620	2.485224	1.953624
6	3.094531	4.762198	0.333203
6	2.887199	3.743175	2.488720
6	2.858053	4.873877	1.692145
1	3.077838	5.652717	-0.285128
1	2.714534	3.840738	3.554977
1	2.656495	5.844693	2.132201
6	3.251407	-1.295586	-2.892532
1	3.497133	-0.417373	-2.297907
6	4.378785	-1.479225	-3.909668
1	5.337194	-1.665583	-3.416799
1	4.185144	-2.320490	-4.581421
1	4.484768	-0.582667	-4.528038
6	1.922742	-0.999908	-3.584546
1	2.014682	-0.117332	-4.224953
1	1.603803	-1.834892	-4.215917
1	1.136948	-0.806078	-2.852793
6	3.587057	-3.431830	1.747200
1	3.551824	-2.372350	2.007845
6	2.508482	-4.135826	2.568555
1	1.511219	-3.762358	2.323921
1	2.510480	-5.217307	2.405141
1	2.677066	-3.968174	3.636419
6	4.976140	-3.957967	2.112354
1	5.169814	-3.833558	3.182266
1	5.067249	-5.023328	1.878669
1	5.762197	-3.433248	1.563397
6	3.586788	3.432138	-1.747064

1	3.551572	2.372656	-2.007709
6	2.508469	4.136211	-2.568680
1	2.677258	3.968470	-3.636497
1	1.511105	3.762886	-2.324237
1	2.510569	5.217704	-2.405348
6	4.975993	3.958203	-2.111884
1	5.761892	3.433430	-1.562754
1	5.169912	3.833808	-3.181752
1	5.067095	5.023554	-1.878145
6	3.250992	1.295906	2.892634
1	3.496801	0.417709	2.298027
6	4.378347	1.479696	3.909770
1	4.484406	0.583190	4.528202
1	5.336744	1.666108	3.416899
1	4.184624	2.320994	4.581457
6	1.922358	1.000071	3.584636
1	1.136597	0.806166	2.852866
1	2.014396	0.117487	4.225017
1	1.603309	1.834995	4.216030
1	5.698099	-1.350231	-0.391509
1	5.356488	0.834320	-1.263598
1	-5.762656	1.223399	-0.677024
1	-5.399321	-1.093140	-1.046392

5-Singlet-B3LYP

7	5.883798	-1.091761	0.284034
6	5.049472	-0.000012	-0.000025
7	5.883789	1.091749	-0.284063
6	7.280799	-0.663882	0.372536
1	7.958905	-1.405772	-0.077774
6	7.280796	0.663893	-0.372554
6	3.651291	-0.000011	-0.000058
6	2.863784	1.217248	0.002469
6	2.863782	-1.217272	-0.002636
6	1.496852	1.210566	0.014226
6	1.496850	-1.210589	-0.014426
6	0.707249	-0.000010	-0.000094
1	3.365729	2.181651	0.029837
1	3.365723	-2.181672	-0.030036
1	0.992075	2.177137	0.048395
1	0.992074	-2.177158	-0.048643
1	7.583535	0.536650	-1.429634
6	5.537418	-2.254959	1.053073
6	5.179670	-2.146778	2.420505
6	5.641840	-3.520788	0.422924
6	4.893570	-3.326639	3.123376
6	5.359435	-4.668824	1.175851
6	4.980647	-4.576441	2.513696
1	4.603520	-3.264711	4.175112
1	5.430397	-5.652319	0.704478
1	4.755905	-5.481330	3.084369
6	5.537405	2.254944	-1.053103
6	5.641774	3.520755	-0.422911
6	5.179744	2.146796	-2.420558
6	5.359420	4.668810	-1.175821
6	4.893676	3.326679	-3.123413
6	4.980708	4.576462	-2.513693
1	5.430343	5.652291	-0.704410
1	4.603702	3.264779	-4.175171
1	4.755991	5.481364	-3.084353
6	6.037266	3.662224	1.044894
1	6.169488	2.644223	1.439762
6	4.930606	4.331184	1.879667
1	4.744455	5.368105	1.554904
1	3.981117	3.779289	1.804145
1	5.217627	4.364915	2.943598
6	7.375814	4.405573	1.206244
1	8.182602	3.920051	0.634114
1	7.304888	5.448275	0.854894
1	7.680168	4.433458	2.265539
6	5.108953	0.812388	-3.160923
1	5.353114	0.013461	-2.447650
6	6.146886	0.743449	-4.297361
1	6.128061	-0.249336	-4.776305
1	5.942605	1.491566	-5.080602
1	7.169571	0.923438	-3.928391
6	3.692258	0.519470	-3.686569

1	2.952479	0.541494	-2.873543
1	3.385020	1.253798	-4.449517
1	3.656107	-0.477890	-4.154484
6	6.037463	-3.662303	-1.044851
1	6.169603	-2.644307	-1.439763
6	4.930975	-4.331436	-1.879703
1	3.981396	-3.779688	-1.804229
1	5.218067	-4.365096	-2.943617
1	4.744966	-5.368396	-1.554990
6	7.376118	-4.405500	-1.206014
1	7.680604	-4.433422	-2.265273
1	8.182784	-3.919842	-0.633828
1	7.305280	-5.448181	-0.854583
6	5.108761	-0.812340	3.160805
1	5.352945	-0.013436	2.447515
6	6.146592	-0.743297	4.297330
1	7.169318	-0.923265	3.928464
1	6.127685	0.249510	4.776223
1	5.942268	-1.491382	5.080590
6	3.692007	-0.519462	3.686316
1	3.655775	0.477912	4.154194
1	2.952299	-0.541541	2.873226
1	3.384731	-1.253777	4.449261
6	-0.707277	-0.000008	-0.000087
6	-1.496879	1.210571	-0.014332
6	-1.496879	-1.210585	0.014163
6	-2.863810	1.217253	-0.002533
6	-2.863811	-1.217268	0.002420
6	-3.651319	-0.000009	-0.000027
1	-0.992103	2.177143	-0.048486
1	-0.992102	-2.177159	0.048266
1	-3.365754	2.181655	-0.029859
1	-3.365753	-2.181673	0.029729
6	-5.049498	0.000000	0.000029
7	-5.883801	1.091762	0.284106
6	-7.280807	0.663900	0.372654
6	-7.280844	-0.663869	-0.372449
7	-5.883841	-1.091745	-0.283995
1	-7.958930	-1.405760	0.077896
1	-7.583498	0.536635	1.429744
6	-5.537484	-2.254943	-1.053043
6	-5.641991	-3.520771	-0.422906
6	-5.179700	-2.146767	-2.420461
6	-5.359660	-4.668817	-1.175839
6	-4.893653	-3.326642	-3.123339
6	-4.980829	-4.576442	-2.513676
1	-5.430695	-5.652311	-0.704474
1	-4.603575	-3.264720	-4.175067
1	-4.756125	-5.481338	-3.084352
6	-5.537390	2.254961	1.053129
6	-5.179505	2.146779	2.420524
6	-5.641894	3.520789	0.422995
6	-4.893388	3.326644	3.123383
6	-5.359465	4.668829	1.175909
6	-4.980566	4.576448	2.513723
1	-4.603232	3.264714	4.175089
1	-5.430500	5.652325	0.704549
1	-4.755805	5.481339	3.084385
6	-6.037627	-3.662270	1.044856
1	-6.169787	-2.644275	1.439760
6	-5.108708	-0.812327	-3.160749
1	-5.352914	-0.013420	-2.447468
6	-6.146473	-0.743255	-4.297334
1	-6.127511	0.249554	-4.776221
1	-5.942130	-1.491344	-5.080584
1	-7.169224	-0.923194	-3.928521
6	-3.691916	-0.519480	-3.686171
1	-2.952259	-0.541599	-2.873035
1	-3.384616	-1.253789	-4.449112
1	-3.655625	0.477902	-4.154028
6	-4.931111	-4.331379	1.879700
1	-4.745028	-5.368302	1.554903
1	-3.981562	-3.779571	1.804298
1	-5.218237	-4.365136	2.943602
6	-7.376262	-4.405498	1.206047
1	-8.182950	-3.919870	0.633865
1	-7.305404	-5.448189	0.854651

1	-7.680718	-4.433405	2.265313
6	-5.108429	0.812328	3.160783
1	-5.352677	0.013430	2.447508
6	-6.146089	0.743205	4.297459
1	-7.168879	0.923132	3.928747
1	-6.127064	-0.249615	4.776321
1	-5.941688	1.491277	5.080710
6	-3.691580	0.519509	3.686074
1	-3.384225	1.253827	4.448985
1	-3.655228	-0.477872	4.153930
1	-2.951997	0.541638	2.872872
6	-6.037642	3.662296	-1.044747
1	-6.169852	2.644297	-1.439628
6	-7.376282	4.405536	-1.205814
1	-7.305377	5.448229	-0.854431
1	-7.680863	4.433427	-2.265046
1	-8.182914	3.919929	-0.633537
6	-4.931194	4.331369	-1.879705
1	-3.981628	3.779591	-1.804287
1	-5.218367	4.365010	-2.943598
1	-4.745126	5.368330	-1.555032
1	-7.958915	1.405805	-0.077629
1	-7.583613	-0.536618	-1.429518
1	7.958885	1.405792	0.077772
1	7.583521	-0.536622	1.429619

5-Triplet-B3LYP

7	-5.927865	1.122895	0.179378
6	-5.095210	0.000012	-0.000006
7	-5.928033	-1.122727	-0.179429
6	-7.318043	0.687263	0.328339
1	-8.015137	1.390463	-0.153572
6	-7.318174	-0.686971	-0.328146
6	-3.671121	-0.000043	-0.000077
6	-2.905408	-1.196384	0.186149
6	-2.905407	1.196290	-0.186328
6	-1.518330	-1.184983	0.184541
6	-1.518325	1.184889	-0.184741
6	-0.768792	-0.000044	-0.000099
1	-3.411925	-2.146399	0.342009
1	-3.411925	2.146301	-0.342212
1	-0.994077	-2.134923	0.315761
1	-0.994081	2.134831	-0.315985
1	-7.597698	-0.621123	-1.397617
6	-5.571711	2.333116	0.867038
6	-5.174256	2.321970	2.228478
6	-5.693901	3.554430	0.155183
6	-4.868465	3.547669	2.838877
6	-5.391241	4.751674	0.818002
6	-4.973514	4.752541	2.147380
1	-4.550208	3.558607	3.884219
1	-5.477129	5.700084	0.281584
1	-4.733186	5.694522	2.647290
6	-5.572045	-2.333022	-0.867018
6	-5.694319	-3.554282	-0.155088
6	-5.174739	-2.321984	-2.228496
6	-5.391927	-4.751600	-0.817894
6	-4.869183	-3.547753	-2.838879
6	-4.974348	-4.752579	-2.147321
1	-5.477929	-5.699979	-0.281437
1	-4.551029	-3.558786	-3.884251
1	-4.734222	-5.694617	-2.647222
6	-6.136433	-3.595956	1.305727
1	-6.266893	-2.552718	1.628271
6	-5.066998	-4.222524	2.218004
1	-4.883772	-5.280330	1.967496
1	-4.107676	-3.688041	2.139271
1	-5.388486	-4.183969	3.271883
6	-7.489369	-4.312018	1.471197
1	-8.270694	-3.855313	0.842884
1	-7.421272	-5.375981	1.189787
1	-7.827912	-4.267813	2.519563
6	-5.094555	-1.045713	-3.065267
1	-5.329815	-0.194261	-2.412401
6	-6.136962	-1.054583	-4.199972
1	-6.111895	-0.101851	-4.754216
1	-5.942610	-1.863498	-4.923076

1	-7.159315	-1.196315	-3.814109
6	-3.679302	-0.802668	-3.619172
1	-2.934134	-0.770566	-2.811425
1	-3.380742	-1.591573	-4.329141
1	-3.639179	0.157979	-4.158208
6	-6.136187	3.596251	-1.305584
1	-6.266939	2.553048	-1.628132
6	-5.066727	4.222578	-2.217987
1	-4.107539	3.687835	-2.139431
1	-5.388406	4.184152	-3.271813
1	-4.883195	5.280330	-1.967475
6	-7.488948	4.312670	-1.470850
1	-7.827703	4.268506	-2.519149
1	-8.270306	3.856234	-0.842381
1	-7.420516	5.376628	-1.189499
6	-5.094191	1.045671	3.065225
1	-5.329637	0.194261	2.412371
6	-6.136495	1.054697	4.200022
1	-7.158863	1.196577	3.814254
1	-6.111516	0.101963	4.754269
1	-5.941959	1.863587	4.923105
6	-3.678932	0.802396	3.619005
1	-3.638912	-0.158270	4.158016
1	-2.933832	0.770198	2.811200
1	-3.380189	1.591235	4.328971
6	0.711996	-0.000046	-0.000081
6	1.459797	-0.920232	0.771167
6	1.459821	0.920137	-0.771309
6	2.846800	-0.929604	0.777395
6	2.846824	0.929515	-0.777489
6	3.606998	-0.000040	-0.000030
1	0.933549	-1.633978	1.410031
1	0.933594	1.633874	-1.410201
1	3.361242	-1.651794	1.409394
1	3.361283	1.651703	-1.409476
6	5.029832	-0.000038	-0.000002
7	5.853715	1.056388	-0.420997
6	7.248353	0.601965	-0.474837
6	7.248326	-0.602019	0.474981
7	5.853697	-1.056458	0.421047
1	7.928210	-1.401460	0.148828
1	7.539742	0.302541	-1.499611
6	5.561339	-2.453645	0.310791
6	5.243315	-3.046551	-0.938689
6	5.641757	-3.244843	1.487558
6	4.993120	-4.426594	-0.973605
6	5.409242	-4.623326	1.390505
6	5.079321	-5.214051	0.172468
1	4.742364	-4.898557	-1.926810
1	5.474267	-5.244329	2.287382
1	4.890480	-6.289223	0.115593
6	5.561332	2.453573	-0.310790
6	5.641802	3.244749	-1.487568
6	5.243231	3.046497	0.938662
6	5.409260	4.623231	-1.390553
6	4.993015	4.426537	0.973540
6	5.079265	5.213973	-0.172544
1	5.474325	5.244218	-2.287437
1	4.742200	4.898515	1.926722
1	4.890405	6.289144	-0.115700
6	5.208699	-2.265214	-2.250807
1	5.382768	-1.206282	-2.020732
6	5.968783	-2.631121	2.847321
1	5.939053	-1.538760	2.720285
6	7.385628	-3.012796	3.315584
1	7.630133	-2.517792	4.270045
1	7.473302	-4.100852	3.471849
1	8.150641	-2.723887	2.578046
6	4.925527	-2.985918	3.921290
1	3.909820	-2.699775	3.607127
1	4.915689	-4.064480	4.147407
1	5.148950	-2.456514	4.862007
6	3.835978	-2.347565	-2.940834
1	3.596258	-3.378431	-3.249753
1	3.034291	-1.995639	-2.275114
1	3.823909	-1.720909	-3.847636
6	6.338114	-2.715308	-3.196889

1	7.327318	-2.622100	-2.720094
1	6.217453	-3.767256	-3.503377
1	6.343249	-2.101598	-4.112947
6	5.968912	2.631007	-2.847301
1	5.939196	1.538648	-2.720245
6	7.385775	3.012700	-3.315494
1	8.150753	2.723821	-2.577908
1	7.630342	2.517682	-4.269931
1	7.473436	4.100755	-3.471776
6	4.925706	2.985762	-3.921332
1	4.915856	4.064320	-4.147467
1	5.149190	2.456346	-4.862027
1	3.909989	2.699601	-3.607218
6	5.208556	2.265181	2.250790
1	5.382652	1.206248	2.020742
6	6.337915	2.715308	3.196925
1	6.343012	2.101612	4.112992
1	7.327145	2.622110	2.720183
1	6.217219	3.767259	3.503389
6	3.835797	2.347522	2.940744
1	3.034152	1.995568	2.274988
1	3.823692	1.720883	3.847558
1	3.596042	3.378390	3.249630
1	7.928205	1.401413	-0.148633
1	7.539639	-0.302588	1.499775
1	-8.015265	-1.390073	0.153933
1	-7.597357	0.621384	1.397862

5-Singlet-Open-Shell-B3LYP $\langle S^2 \rangle = 0.0046$

7	5.883798	-1.091761	0.284034
6	5.049472	-0.000012	-0.000025
7	5.883789	1.091749	-0.284063
6	7.280799	-0.663882	0.372536
1	7.958905	-1.405772	-0.077774
6	7.280796	0.663893	-0.372554
6	3.651291	-0.000011	-0.000058
6	2.863784	1.217248	0.002469
6	2.863782	-1.217272	-0.002636
6	1.496852	1.210566	0.014226
6	1.496850	-1.210589	-0.014426
6	0.707249	-0.000010	-0.000094
1	3.365729	2.181651	0.029837
1	3.365723	-2.181672	-0.030036
1	0.992075	2.177137	0.048395
1	0.992074	-2.177158	-0.048643
1	7.583535	0.536650	-1.429634
6	5.537418	-2.254959	1.053073
6	5.179670	-2.146778	2.420505
6	5.641840	-3.520788	0.422924
6	4.893570	-3.326639	3.123376
6	5.359435	-4.668824	1.175851
6	4.980647	-4.576441	2.513696
1	4.603520	-3.264711	4.175112
1	5.430397	-5.652319	0.704478
1	4.755905	-5.481330	3.084369
6	5.537405	2.254944	-1.053103
6	5.641774	3.520755	-0.422911
6	5.179744	2.146796	-2.420558
6	5.359420	4.668810	-1.175821
6	4.893676	3.326679	-3.123413
6	4.980708	4.576462	-2.513693
1	5.430343	5.652291	-0.704410
1	4.603702	3.264779	-4.175171
1	4.755991	5.481364	-3.084353
6	6.037266	3.662224	1.044894
1	6.169488	2.644223	1.439762
6	4.930606	4.331184	1.879667
1	4.744455	5.368105	1.554904
1	3.981117	3.779289	1.804145
1	5.217627	4.364915	2.943598
6	7.375814	4.405573	1.206244
1	8.182602	3.920051	0.634114
1	7.304888	5.448275	0.854894
1	7.680168	4.433458	2.265539
6	5.108953	0.812388	-3.160923
1	5.353114	0.013461	-2.447650
6	6.146886	0.743449	-4.297361

1	6.128061	-0.249336	-4.776305
1	5.942605	1.491566	-5.080602
1	7.169571	0.923438	-3.928391
6	3.692258	0.519470	-3.686569
1	2.952479	0.541494	-2.873543
1	3.385020	1.253798	-4.449517
1	3.656107	-0.477890	-4.154484
6	6.037463	-3.662303	-1.044851
1	6.169603	-2.644307	-1.439763
6	4.930975	-4.331436	-1.879703
1	3.981396	-3.779688	-1.804229
1	5.218067	-4.365096	-2.943617
1	4.744966	-5.368396	-1.554990
6	7.376118	-4.405500	-1.206014
1	7.680604	-4.433422	-2.265273
1	8.182784	-3.919842	-0.633828
1	7.305280	-5.448181	-0.854583
6	5.108761	-0.812340	3.160805
1	5.352945	-0.013436	2.447515
6	6.146592	-0.743297	4.297330
1	7.169318	-0.923265	3.928464
1	6.127685	0.249510	4.776223
1	5.942268	-1.491382	5.080590
6	3.692007	-0.519462	3.686316
1	3.655775	0.477912	4.154194
1	2.952299	-0.541541	2.873226
1	3.384731	-1.253777	4.449261
6	-0.707277	-0.000008	-0.000087
6	-1.496879	1.210571	-0.014332
6	-1.496879	-1.210585	0.014163
6	-2.863810	1.217253	-0.002533
6	-2.863811	-1.217268	0.002420
6	-3.651319	-0.000009	-0.000027
1	-0.992103	2.177143	-0.048486
1	-0.992102	-2.177159	0.048266
1	-3.365754	2.181655	-0.029859
1	-3.365753	-2.181673	0.029729
6	-5.049498	0.000000	0.000029
7	-5.883801	1.091762	0.284106
6	-7.280807	0.663900	0.372654
6	-7.280844	-0.663869	-0.372449
7	-5.883841	-1.091745	-0.283995
1	-7.958930	-1.405760	0.077896
1	-7.583498	0.536635	1.429744
6	-5.537484	-2.254943	-1.053043
6	-5.641991	-3.520771	-0.422906
6	-5.179700	-2.146767	-2.420461
6	-5.359660	-4.668817	-1.175839
6	-4.893653	-3.326642	-3.123339
6	-4.980829	-4.576442	-2.513676
1	-5.430695	-5.652311	-0.704474
1	-4.603575	-3.264720	-4.175067
1	-4.756125	-5.481338	-3.084352
6	-5.537390	2.254961	1.053129
6	-5.179505	2.146779	2.420524
6	-5.641894	3.520789	0.422995
6	-4.893388	3.326644	3.123383
6	-5.359465	4.668829	1.175909
6	-4.980566	4.576448	2.513723
1	-4.603232	3.264714	4.175089
1	-5.430500	5.652325	0.704549
1	-4.755805	5.481339	3.084385
6	-6.037627	-3.662270	1.044856
1	-6.169787	-2.644275	1.439760
6	-5.108708	-0.812327	-3.160749
1	-5.352914	-0.013420	-2.447468
6	-6.146473	-0.743255	-4.297334
1	-6.127511	0.249554	-4.776221
1	-5.942130	-1.491344	-5.080584
1	-7.169224	-0.923194	-3.928521
6	-3.691916	-0.519480	-3.686171
1	-2.952259	-0.541599	-2.873035
1	-3.384616	-1.253789	-4.449112
1	-3.655625	0.477902	-4.154028
6	-4.931111	-4.331379	1.879700
1	-4.745028	-5.368302	1.554903
1	-3.981562	-3.779571	1.804298

1	-5.218237	-4.365136	2.943602
6	-7.376262	-4.405498	1.206047
1	-8.182950	-3.919870	0.633865
1	-7.305404	-5.448189	0.854651
1	-7.680718	-4.433405	2.265313
6	-5.108429	0.812328	3.160783
1	-5.352677	0.013430	2.447508
6	-6.146089	0.743205	4.297459
1	-7.168879	0.923132	3.928747
1	-6.127064	-0.249615	4.776321
1	-5.941688	1.491277	5.080710
6	-3.691580	0.519509	3.686074
1	-3.384225	1.253827	4.448985
1	-3.655228	-0.477872	4.153930
1	-2.951997	0.541638	2.872872
6	-6.037642	3.662296	-1.044747
1	-6.169852	2.644297	-1.439628
6	-7.376282	4.405536	-1.205814
1	-7.305377	5.448229	-0.854431
1	-7.680863	4.433427	-2.265046
1	-8.182914	3.919929	-0.633537
6	-4.931194	4.331369	-1.879705
1	-3.981628	3.779591	-1.804287
1	-5.218367	4.365010	-2.943598
1	-4.745126	5.368330	-1.555032
1	-7.958915	1.405805	-0.077629
1	-7.583613	-0.536618	-1.429518
1	7.958885	1.405792	0.077772
1	7.583521	-0.536622	1.429619

5-Singlet-BH&HLYP

7	-5.835024	1.084381	0.265864
6	-5.005307	0.000044	-0.000059
7	-5.835049	-1.084241	-0.266079
6	-7.222528	0.667675	0.355070
1	-7.888768	1.396346	-0.113272
6	-7.222508	-0.667451	-0.355539
6	-3.625499	0.000027	0.000006
6	-2.837055	-1.214084	0.001283
6	-2.837035	1.214128	-0.001214
6	-1.486358	-1.209466	0.013466
6	-1.486337	1.209485	-0.013311
6	-0.694459	0.000003	0.000087
1	-3.333982	-2.171383	0.027637
1	-3.333938	2.171439	-0.027595
1	-0.986057	-2.168414	0.046347
1	-0.986019	2.168426	-0.046153
1	-7.529062	-0.566381	-1.404646
6	-5.491165	2.251837	1.010133
6	-5.151276	2.163206	2.371048
6	-5.576667	3.496701	0.363054
6	-4.860333	3.341738	3.053516
6	-5.290210	4.646594	1.092579
6	-4.926568	4.572925	2.425747
1	-4.582172	3.294162	4.100462
1	-5.345850	5.614979	0.607515
1	-4.697900	5.477797	2.978520
6	-5.491115	-2.251789	-1.010169
6	-5.576765	-3.496580	-0.362980
6	-5.151046	-2.163306	-2.371045
6	-5.290274	-4.646563	-1.092352
6	-4.860098	-3.341921	-3.053368
6	-4.926464	-4.573042	-2.425483
1	-5.346044	-5.614897	-0.607204
1	-4.581806	-3.294465	-4.100285
1	-4.697781	-5.477981	-2.978140
6	-5.957071	-3.615427	1.101709
1	-6.099573	-2.601158	1.477526
6	-4.842716	-4.251955	1.931923
1	-4.648617	-5.283576	1.623590
1	-3.908365	-3.693209	1.839729
1	-5.119342	-4.271323	2.990348
6	-7.274325	-4.369851	1.282693
1	-8.084439	-3.907295	0.712205
1	-7.190223	-5.408673	0.949521
1	-7.568084	-4.384298	2.336591

6	-5.098559	-0.846101	-3.125490
1	-5.362230	-0.047711	-2.431901
6	-6.119687	-0.813898	-4.263860
1	-6.115730	0.165524	-4.751467
1	-5.893870	-1.562437	-5.028759
1	-7.133631	-1.008517	-3.903042
6	-3.692134	-0.539568	-3.639382
1	-2.965508	-0.534294	-2.825206
1	-3.368965	-1.277655	-4.379834
1	-3.670167	0.442321	-4.121344
6	-5.956696	3.615717	-1.101699
1	-6.099257	2.601500	-1.477628
6	-4.842104	4.252156	-1.931659
1	-3.907853	3.693266	-1.839332
1	-5.118536	4.271641	-2.990135
1	-4.647911	5.283726	-1.623217
6	-7.273825	4.370322	-1.282858
1	-7.567394	4.384923	-2.336808
1	-8.084102	3.907807	-0.712568
1	-7.189653	5.409093	-0.949552
6	-5.098945	0.845930	3.125380
1	-5.362708	0.047626	2.431730
6	-6.120084	0.813748	4.263742
1	-7.134002	1.008494	3.902923
1	-6.116229	-0.165712	4.751273
1	-5.894203	1.562204	5.028703
6	-3.692556	0.539203	3.639251
1	-3.670688	-0.442733	4.121121
1	-2.965927	0.533931	2.825076
1	-3.369317	1.277191	4.379773
6	0.694468	-0.000008	0.000093
6	1.486347	-1.209490	-0.013269
6	1.486365	1.209462	0.013449
6	2.837046	-1.214131	-0.001171
6	2.837063	1.214082	0.001271
6	3.625508	-0.000030	0.000016
1	0.986030	-2.168432	-0.046081
1	0.986063	2.168410	0.046314
1	3.333948	-2.171444	-0.027526
1	3.333987	2.171383	0.027614
6	5.005316	-0.000046	-0.000055
7	5.835036	-1.084383	0.265874
6	7.222528	-0.667649	0.355128
6	7.222509	0.667413	-0.355590
7	5.835064	1.084235	-0.266084
1	7.888921	1.396066	0.112530
1	7.529206	-0.566491	1.404192
6	5.491134	2.251793	-1.010158
6	5.576798	3.496575	-0.362955
6	5.151053	2.163329	-2.371034
6	5.290320	4.646570	-1.092312
6	4.860113	3.341956	-3.053340
6	4.926500	4.573070	-2.425441
1	5.346108	5.614898	-0.607154
1	4.581808	3.294515	-4.100254
1	4.697822	5.478017	-2.978086
6	5.491172	-2.251844	1.010134
6	5.151248	-2.163210	2.371039
6	5.576688	-3.496709	0.363058
6	4.860292	-3.341741	3.053504
6	5.290216	-4.646601	1.092579
6	4.926545	-4.572930	2.425739
1	4.582103	-3.294163	4.100443
1	5.345864	-5.614987	0.607518
1	4.697864	-5.477801	2.978508
6	5.957105	3.615400	1.101736
1	6.099610	2.601124	1.477535
6	5.098542	0.846133	-3.125491
1	5.362194	0.047730	-2.431909
6	6.119681	0.813918	-4.263851
1	6.115708	-0.165499	-4.751467
1	5.893891	1.562469	-5.028747
1	7.133623	1.008511	-3.903015
6	3.692115	0.539634	-3.639400
1	2.965477	0.534365	-2.825234
1	3.368972	1.277738	-4.379848
1	3.670133	-0.442248	-4.121375

6	4.842743	4.251903	1.931958
1	4.648636	5.283529	1.623650
1	3.908398	3.693150	1.839743
1	5.119365	4.271248	2.990386
6	7.274355	4.369829	1.282733
1	8.084471	3.907291	0.712234
1	7.190243	5.408656	0.949580
1	7.568116	4.384257	2.336631
6	5.098879	-0.845929	3.125359
1	5.362662	-0.047629	2.431712
6	6.119975	-0.813727	4.263758
1	7.133908	-1.008462	3.902973
1	6.116092	0.165737	4.751282
1	5.894077	-1.562181	5.028716
6	3.692465	-0.539207	3.639168
1	3.369198	-1.277192	4.379680
1	3.670569	0.442733	4.121029
1	2.965873	-0.533946	2.824959
6	5.956737	-3.615732	-1.101690
1	6.099332	-2.601518	-1.477614
6	7.273845	-4.370376	-1.282832
1	7.189639	-5.409144	-0.949525
1	7.567427	-4.384988	-2.336778
1	8.084129	-3.907884	-0.712533
6	4.842137	-4.252134	-1.931666
1	3.907899	-3.693220	-1.839343
1	5.118578	-4.271619	-2.990140
1	4.647913	-5.283702	-1.623233
1	7.888808	-1.396348	-0.113110
1	7.528996	0.566235	-1.404708
1	-7.888877	-1.396078	0.112686
1	-7.529272	0.566622	1.404123

5-Triplet-BH&HLYP

7	-5.886569	1.101960	0.246516
6	-5.057436	0.000057	-0.000180
7	-5.886656	-1.101780	-0.246917
6	-7.264820	0.660838	0.368024
1	-7.956563	1.386863	-0.066031
6	-7.264816	-0.660474	-0.368781
6	-3.644756	0.000019	-0.000140
6	-2.886499	-1.199102	0.106496
6	-2.886489	1.199132	-0.106767
6	-1.507849	-1.187219	0.105555
6	-1.507839	1.187253	-0.105772
6	-0.769950	0.000016	-0.000086
1	-3.389994	-2.149344	0.199297
1	-3.389987	2.149368	-0.199623
1	-0.982645	-2.133697	0.177248
1	-0.982635	2.133730	-0.177463
1	-7.541730	-0.527624	-1.423146
6	-5.531254	2.248888	1.017314
6	-5.152325	2.135605	2.367014
6	-5.634125	3.509413	0.401114
6	-4.842412	3.300481	3.064958
6	-5.328000	4.644558	1.145604
6	-4.926349	4.544594	2.465917
1	-4.536994	3.231623	4.103125
1	-5.399406	5.622671	0.682512
1	-4.683067	5.438033	3.030939
6	-5.531320	-2.248847	-1.017490
6	-5.634443	-3.509274	-0.401106
6	-5.152061	-2.135813	-2.367113
6	-5.328186	-4.644552	-1.145329
6	-4.842036	-3.300827	-3.064788
6	-4.926190	-4.544829	-2.465562
1	-5.399771	-5.622589	-0.682106
1	-4.536338	-3.232151	-4.102885
1	-4.682814	-5.438378	-3.030370
6	-6.065141	-3.660899	1.046699
1	-6.201724	-2.654202	1.443772
6	-4.993870	-4.342340	1.897327
1	-4.807830	-5.369125	1.568679
1	-4.045721	-3.800854	1.853934
1	-5.307915	-4.384279	2.944616

6	-7.401680	-4.394398	1.161430
1	-8.182448	-3.900333	0.576788
1	-7.325183	-5.424966	0.801579
1	-7.732544	-4.432236	2.203767
6	-5.089432	-0.807326	-3.101949
1	-5.343566	-0.016700	-2.396328
6	-6.114775	-0.751724	-4.235911
1	-6.104291	0.233442	-4.711899
1	-5.898890	-1.493910	-5.009934
1	-7.128685	-0.941483	-3.872877
6	-3.683792	-0.504661	-3.620468
1	-2.951378	-0.518254	-2.811552
1	-3.370537	-1.232165	-4.375367
1	-3.656479	0.484940	-4.086173
6	-6.064523	3.661336	-1.046749
1	-6.200545	2.654710	-1.444196
6	-4.993386	4.343582	-1.896890
1	-4.044982	3.802543	-1.853450
1	-5.307200	4.385710	-2.944240
1	-4.807924	5.370353	-1.567875
6	-7.401390	4.394247	-1.161492
1	-7.732051	4.432350	-2.203885
1	-8.182056	3.899577	-0.577222
1	-7.325459	5.424701	-0.801191
6	-5.089966	0.806985	3.101633
1	-5.343991	0.016505	2.395809
6	-6.115574	0.751236	4.235345
1	-7.129390	0.941138	3.872124
1	-6.105269	-0.234023	4.711144
1	-5.899814	1.493251	5.009568
6	-3.684466	0.504133	3.620430
1	-3.657326	-0.485556	4.085958
1	-2.951854	0.517832	2.811695
1	-3.371353	1.231480	4.375537
6	0.709755	0.000014	-0.000038
6	1.446008	-0.869160	0.817711
6	1.446072	0.869182	-0.817733
6	2.824507	-0.878777	0.824760
6	2.824574	0.878803	-0.824672
6	3.577833	0.000020	0.000082
1	0.918808	-1.541602	1.486093
1	0.918926	1.541619	-1.486162
1	3.335494	-1.559392	1.490126
1	3.335606	1.559418	-1.490003
6	4.988709	0.000005	0.000163
7	5.809166	1.015463	-0.493981
6	7.193337	0.567410	-0.507776
6	7.193196	-0.567373	0.508695
7	5.809040	-1.015472	0.494487
1	7.863744	-1.381736	0.231027
1	7.491611	0.209302	-1.501643
6	5.519590	-2.407975	0.471245
6	5.234916	-3.075889	-0.733674
6	5.569546	-3.113705	1.688577
6	4.982009	-4.445062	-0.686421
6	5.334770	-4.484931	1.679127
6	5.034597	-5.149211	0.502881
1	4.753964	-4.973647	-1.605418
1	5.376017	-5.040945	2.609151
1	4.843573	-6.216923	0.512696
6	5.519650	2.407954	-0.471069
6	5.569819	3.113468	-1.688513
6	5.234714	3.076063	0.733680
6	5.334983	4.484687	-1.679348
6	4.981766	4.445218	0.686142
6	5.034556	5.149159	-0.503276
1	5.376388	5.040541	-2.609460
1	4.753523	4.973956	1.605003
1	4.843492	6.216862	-0.513315
6	5.227628	-2.382229	-2.084654
1	5.424789	-1.323690	-1.922529
6	5.874169	-2.414634	3.001490
1	5.842346	-1.341700	2.804404
6	7.277375	-2.757903	3.503788
1	7.508406	-2.204454	4.419133
1	7.364870	-3.825301	3.729010
1	8.041442	-2.515858	2.760712

6	4.827980	-2.704441	4.076619
1	3.821989	-2.448302	3.735227
1	4.823363	-3.758085	4.369731
1	5.036310	-2.117131	4.975759
6	3.865576	-2.478444	-2.770559
1	3.607925	-3.514902	-3.008621
1	3.075720	-2.069200	-2.137671
1	3.871635	-1.917091	-3.709641
6	6.342052	-2.914575	-2.986808
1	7.322612	-2.816369	-2.512343
1	6.196979	-3.971990	-3.226492
1	6.366214	-2.362591	-3.931373
6	5.874732	2.414179	-3.001241
1	5.842949	1.341279	-2.803966
6	7.278008	2.757455	-3.503337
1	8.041947	2.515604	-2.760068
1	7.509253	2.203850	-4.418534
1	7.365470	3.824817	-3.728745
6	4.828716	2.703715	-4.076611
1	4.824062	3.757310	-4.369900
1	5.037261	2.116272	-4.975615
1	3.822685	2.447545	-3.735358
6	5.227190	2.382619	2.084768
1	5.424359	1.324050	1.922840
6	6.341473	2.915096	2.987018
1	6.365468	2.363273	3.931681
1	7.322111	2.816794	2.512734
1	6.196378	3.972556	3.226495
6	3.865027	2.478961	2.770437
1	3.075268	2.069633	2.137482
1	3.870927	1.917748	3.709603
1	3.607353	3.515457	3.008303
1	7.863773	1.381792	-0.229886
1	7.491143	-0.209238	1.502649
1	-7.956762	-1.386403	0.065111
1	-7.542010	0.528013	1.422319

5-Singlet-Open-Shell-BH&HLYP $<S^2>$= 0.4252

7	-5.835024	1.084381	0.265864
6	-5.005307	0.000044	-0.000059
7	-5.835049	-1.084241	-0.266079
6	-7.222528	0.667675	0.355070
1	-7.888768	1.396346	-0.113272
6	-7.222508	-0.667451	-0.355539
6	-3.625499	0.000027	0.000006
6	-2.837055	-1.214084	0.001283
6	-2.837035	1.214128	-0.001214
6	-1.486358	-1.209466	0.013466
6	-1.486337	1.209485	-0.013311
6	-0.694459	0.000003	0.000087
1	-3.333982	-2.171383	0.027637
1	-3.333938	2.171439	-0.027595
1	-0.986057	-2.168414	0.046347
1	-0.986019	2.168426	-0.046153
1	-7.529062	-0.566381	-1.404646
6	-5.491165	2.251837	1.010133
6	-5.151276	2.163206	2.371048
6	-5.576667	3.496701	0.363054
6	-4.860333	3.341738	3.053516
6	-5.290210	4.646594	1.092579
6	-4.926568	4.572925	2.425747
1	-4.582172	3.294162	4.100462
1	-5.345850	5.614979	0.607515
1	-4.697900	5.477797	2.978520
6	-5.491115	-2.251789	-1.010169
6	-5.576765	-3.496580	-0.362980
6	-5.151046	-2.163306	-2.371045
6	-5.290274	-4.646563	-1.092352
6	-4.860098	-3.341921	-3.053368
6	-4.926464	-4.573042	-2.425483
1	-5.346044	-5.614897	-0.607204
1	-4.581806	-3.294465	-4.100285
1	-4.697781	-5.477981	-2.978140
6	-5.957071	-3.615427	1.101709
1	-6.099573	-2.601158	1.477526
6	-4.842716	-4.251955	1.931923
1	-4.648617	-5.283576	1.623590

1	-3.908365	-3.693209	1.839729
1	-5.119342	-4.271323	2.990348
6	-7.274325	-4.369851	1.282693
1	-8.084439	-3.907295	0.712205
1	-7.190223	-5.408673	0.949521
1	-7.568084	-4.384298	2.336591
6	-5.098559	-0.846101	-3.125490
1	-5.362230	-0.047711	-2.431901
6	-6.119687	-0.813898	-4.263860
1	-6.115730	0.165524	-4.751467
1	-5.893870	-1.562437	-5.028759
1	-7.133631	-1.008517	-3.903042
6	-3.692134	-0.539568	-3.639382
1	-2.965508	-0.534294	-2.825206
1	-3.368965	-1.277655	-4.379834
1	-3.670167	0.442321	-4.121344
6	-5.956696	3.615717	-1.101699
1	-6.099257	2.601500	-1.477628
6	-4.842104	4.252156	-1.931659
1	-3.907853	3.693266	-1.839332
1	-5.118536	4.271641	-2.990135
1	-4.647911	5.283726	-1.623217
6	-7.273825	4.370322	-1.282858
1	-7.567394	4.384923	-2.336808
1	-8.084102	3.907807	-0.712568
1	-7.189653	5.409093	-0.949552
6	-5.098945	0.845930	3.125380
1	-5.362708	0.047626	2.431730
6	-6.120084	0.813748	4.263742
1	-7.134002	1.008494	3.902923
1	-6.116229	-0.165712	4.751273
1	-5.894203	1.562204	5.028703
6	-3.692556	0.539203	3.639251
1	-3.670688	-0.442733	4.121121
1	-2.965927	0.533931	2.825076
1	-3.369317	1.277191	4.379773
6	0.694468	-0.000008	0.000093
6	1.486347	-1.209490	-0.013269
6	1.486365	1.209462	0.013449
6	2.837046	-1.214131	-0.001171
6	2.837063	1.214082	0.001271
6	3.625508	-0.000030	0.000016
1	0.986030	-2.168432	-0.046081
1	0.986063	2.168410	0.046314
1	3.333948	-2.171444	-0.027526
1	3.333987	2.171383	0.027614
6	5.005316	-0.000046	-0.000055
7	5.835036	-1.084383	0.265874
6	7.222528	-0.667649	0.355128
6	7.222509	0.667413	-0.355590
7	5.835064	1.084235	-0.266084
1	7.888921	1.396066	0.112530
1	7.529206	-0.566491	1.404192
6	5.491134	2.251793	-1.010158
6	5.576798	3.496575	-0.362955
6	5.151053	2.163329	-2.371034
6	5.290320	4.646570	-1.092312
6	4.860113	3.341956	-3.053340
6	4.926500	4.573070	-2.425441
1	5.346108	5.614898	-0.607154
1	4.581808	3.294515	-4.100254
1	4.697822	5.478017	-2.978086
6	5.491172	-2.251844	1.010134
6	5.151248	-2.163210	2.371039
6	5.576688	-3.496709	0.363058
6	4.860292	-3.341741	3.053504
6	5.290216	-4.646601	1.092579
6	4.926545	-4.572930	2.425739
1	4.582103	-3.294163	4.100443
1	5.345864	-5.614987	0.607518
1	4.697864	-5.477801	2.978508
6	5.957105	3.615400	1.101736
1	6.099610	2.601124	1.477535
6	5.098542	0.846133	-3.125491
1	5.362194	0.047730	-2.431909
6	6.119681	0.813918	-4.263851
1	6.115708	-0.165499	-4.751467

1	5.893891	1.562469	-5.028747
1	7.133623	1.008511	-3.903015
6	3.692115	0.539634	-3.639400
1	2.965477	0.534365	-2.825234
1	3.368972	1.277738	-4.379848
1	3.670133	-0.442248	-4.121375
6	4.842743	4.251903	1.931958
1	4.648636	5.283529	1.623650
1	3.908398	3.693150	1.839743
1	5.119365	4.271248	2.990386
6	7.274355	4.369829	1.282733
1	8.084471	3.907291	0.712234
1	7.190243	5.408656	0.949580
1	7.568116	4.384257	2.336631
6	5.098879	-0.845929	3.125359
1	5.362662	-0.047629	2.431712
6	6.119975	-0.813727	4.263758
1	7.133908	-1.008462	3.902973
1	6.116092	0.165737	4.751282
1	5.894077	-1.562181	5.028716
6	3.692465	-0.539207	3.639168
1	3.369198	-1.277192	4.379680
1	3.670569	0.442733	4.121029
1	2.965873	-0.533946	2.824959
6	5.956737	-3.615732	-1.101690
1	6.099332	-2.601518	-1.477614
6	7.273845	-4.370376	-1.282832
1	7.189639	-5.409144	-0.949525
1	7.567427	-4.384988	-2.336778
1	8.084129	-3.907884	-0.712533
6	4.842137	-4.252134	-1.931666
1	3.907899	-3.693220	-1.839343
1	5.118578	-4.271619	-2.990140
1	4.647913	-5.283702	-1.623233
1	7.888808	-1.396348	-0.113110
1	7.528996	0.566235	-1.404708
1	-7.888877	-1.396078	0.112686
1	-7.529272	0.566622	1.404123

References

1. O. V. Dolomanov, L. J. Bourhis, R. J. Gildea, J. A. K. Howard and H. Puschmann, *J. Appl. Cryst.*, 2009, **42**, 339–341.
2. G. Sheldrick, *Acta Cryst. A*, 2015, **71**, 3–8.
3. G. Sheldrick, *Acta Cryst. C*, 2015, **71**, 3–8.
4. M. J. Frisch, G. W. Trucks, H. B. Schlegel, G. E. Scuseria, M. A. Robb, J. R. Cheeseman, G. Scalmani, V. Barone, B. Mennucci, G. A. Petersson, H. Nakatsuji, M. Caricato, X. Li, H. P. Hratchian, A. F. Izmaylov, J. Bloino, G. Zheng, J. L. Sonnenberg, M. Hada, M. Ehara, K. Toyota, R. Fukuda, J. Hasegawa, M. Ishida, T. Nakajima, Y. Honda, O. Kitao, H. Nakai, T. Vreven, J. J. A. Montgomery, J. E. Peralta, F. Ogliaro, M. Bearpark, J. J. Heyd, E. Brothers, K. N. Kudin, V. N. Staroverov, R. Kobayashi, J. Normand, K. Raghavachari, A. Rendell, J. C. Burant, S. S. Iyengar, J. Tomasi, M. Cossi, N. Rega, J. M. Millam, M. Klene, J. E. Knox, J. B. Cross, V. Bakken, C. Adamo, J. Jaramillo, R. Gomperts, R. E. Stratmann, O. Yazyev, A. J. Austin, R. Cammi, C. Pomelli, J. W. Ochterski, R. L. Martin, K. Morokuma, V. G. Zakrzewski, G. A. Voth, P. Salvador, J. J. Dannenberg, S. Dapprich, A. D. Daniels, O. Farkas, J. B. Foresman, J. V. Ortiz, J. Cioslowski and D. J. Fox, Gaussian 09, Revision C.01. Gaussian, Inc., Wallingford CT, 2009.
5. M. Bruschi, P. Fantucci, M. Pizzotti and C. Rovizzi, *J. Mol. Catal. A*, 2003, **204**, 793–803.
6. (a) A. D. Becke, *J. Chem. Phys.*, 1993, **98**, 5648–5652; (b) C. Lee, W. Yang and R. G. Parr, *Phys. Rev. B*, 1988, **37**, 785–789.
7. A. D. Becke, *J. Chem. Phys.*, 1993, **98**, 1372–1377.
8. F. Weigend and R. Ahlrichs, *Phys. Chem. Chem. Phys.*, 2005, **7**, 3297–3305.
9. C. Y. Peng, P. Y. Ayala, H. B. Schlegel and M. J. Frisch, *J. Comput. Chem.*, 1996, **17**, 49–56.
10. C. Adamo and V. Barone, *J. Chem. Phys.*, 1999, **110**, 6158–6170.
11. Y. Zhao and D. G. Truhlar, *Theor. Chem. Acc.*, 2008, **120**, 215–241.
12. F. Neese, Max-Planck institute for Bioinorganic Chemistry; Mulheim an der Ruhr, Germany, 4.0.1 edn., 2005.
13. A. Klamt and G. Schuurmann, *J. Chem. Soc.-Perkin Trans. 2*, 1993, 799–805.
14. D. Herebian, E. Bothe, F. Neese, T. Weyhermüller and K. Wieghardt, *J. Am. Chem. Soc.*, 2003, **125**, 9116–9128.
15. (a) V. Bachler, G. Olbrich, F. Neese and K. Wieghardt, *Inorg. Chem.*, 2002, **41**, 4179–4193; (b) F. Neese, *J. Phys. Chem. Solids*, 2004, **65**, 781–785; (c) D. Herebian, K. E. Wieghardt and F. Neese, *J. Am. Chem. Soc.*, 2003, **125**, 10997–11005.
